# Inhibition of melanization by serpin-5 and serpin-9 promotes baculovirus infection in cotton bollworm *Helicoverpa armigera*

**DOI:** 10.1371/journal.ppat.1006645

**Published:** 2017-09-27

**Authors:** Chuanfei Yuan, Longsheng Xing, Manli Wang, Xi Wang, Mengyi Yin, Qianran Wang, Zhihong Hu, Zhen Zou

**Affiliations:** 1 State Key Laboratory of Virology, Wuhan Institute of Virology, Chinese Academy of Sciences, Wuhan, China; 2 University of Chinese Academy of Sciences, Beijing, China; 3 State Key Laboratory of Integrated Management of Pest Insects and Rodents, Institute of Zoology, Chinese Academy of Sciences, Beijing, China; Iowa State University, UNITED STATES

## Abstract

Melanization, an important insect defense mechanism, is mediated by clip-domain serine protease (cSP) cascades and is regulated by serpins. Here we show that proteolytic activation of prophenoloxidase (PPO) and PO-catalyzed melanization kill the baculovirus *in vitro*. Our quantitative proteomics and biochemical experiments revealed that baculovirus infection of the cotton bollworm, *Helicoverpa armigera*, reduced levels of most cascade members in the host hemolymph and PO activity. By contrast, serpin-9 and serpin-5 were sequentially upregulated after the viral infection. The *H*. *armigera* serpin-5 and serpin-9 regulate melanization by directly inhibiting their target proteases cSP4 and cSP6, respectively and cSP6 activates PPO purified from hemolymph. Furthermore, serpin-5/9-depleted insects exhibited high PO activities and showed resistance to baculovirus infection. Together, our results characterize a part of the melanization cascade in *H*. *armigera*, and suggest that natural insect virus baculovirus has evolved a distinct strategy to suppress the host immune system.

## Author summary

Melanization is one of important modules in insect defense system. It consists of a cascade of clip-domain serine proteases (cSPs) that converts the zymogen prophenoloxidase (PPO) to active phenoloxidase (PO), which is negatively regulated by serpins. PO then catalyses the formation of melanin that physically encapsulates certain pathogens. Parasites and bacteria have evolved to produce specific proteins or antibiotic to suppress the melanization response of host insects for survival. However, the mechanisms by which virus persists in the face of the insect melanization are poorly understood. In this study, we show that a DNA virus baculovirus infection of the cotton bollworm, *Helicoverpa armigera*, reduced the levels of most cascade members in the host hemolymph and PO activity. By contrast, serpin-9 and serpin-5 were sequentially upregulated after the viral infection. Our results also reveal that melanization kills baculovirus *in vitro*. Serpin-5 and serpin-9 regulate melanization by directly inhibiting their target proteases cSP4 and cSP6, respectively and cSP6 activates PPO purified from hemolymph. Moreover, serpin-5/9-depleted insects show resistance to baculovirus infection. Our findings have enriched the understanding of molecular mechanisms by which pathogens suppress the melanization response of host insect for survival.

## Introduction

The cotton bollworm, *Helicoverpa armigera*, is a major pest of cotton and one of the most destructive and polyphagous pest species. It is widespread in central and southern Europe, temperate Asia, Africa, Australia and Oceania, and Brazil [1]. Moreover, the rapid spread of *H*. *armigera* through South America into Central America has threatened agriculture in North America and an immense quantity of crops are exposed to *H*. *armigera* each year [2]. Due to the broad-spectrum pesticide resistance developed in *H*. *armigera*, traditional chemical insecticides do not protect these crops from significant production impact [2] and transgenic crops also lead to resistance development in pests [3], highlighting the urgent need to develop effective biopesticide alternatives to control pests. H. armigera nucleopolyhedrovirus (HearNPV), a natural enemy of *H*. *armigera*, has increasingly been used as a biological insecticide for pest control in recent decades [4,5]. Before the baculovirus kills the pest insects, it must invade and overcome the host immune responses. However, molecular interactions between the virus and its host’s immune system are poorly understood.

Viruses are a major burden for invertebrate organisms. Besides being used as biological agents against pest insects, these obligate intracellular pathogens have an important impact on economically beneficial insects [6]. However, the immunity to viral infection is still poorly understood in invertebrates. An important defense against viruses in invertebrates is RNA interference (RNAi), which can efficiently degrade virally transcribed RNA [7]. On the other hand, two additional cellular mechanisms, apoptosis and autophagy restrict viral replication and dissemination [6]. Moreover, several studies based on invertebrate larvae, have suggested that melanization is involved in antiviral immunity. Melanization is an important defense mechanism in arthropods and plays an essential role in wound healing and innate immunity [8]. In the mosquito *Aedes aegypti*, phenoloxidase (PO) activity is required for defense against the Semliki Forest virus [9]. In the shrimp *Penaeus monodon*, the white spot syndrome virus (WSSV) has evolved a specific strategy to counteract the host’s melanization response [10].

Studies in a few insects revealed that melanization is initiated by the recognition of microbial elicitors such as β-1, 3-glucan, lipopolysaccharide, and peptidoglycan by pathogen recognition receptors [8]. Upon binding, a serine protease (SP) cascade is activated to finally cleave the PPO zymogen to active PO. PO catalyzes the formation of quinones and melanin, which kill and immobilize microbes [8,11]. Biochemical studies of *Manduca sexta*, *Tenebrio molitor* and *Holotrichia diomphalia* have indicated that SP cascades are mainly composed of clip-domain serine proteases (cSPs) that harbor regulatory clip domain(s) in their N- termini. In *M*. *sexta*, the biochemical model insect, two SP cascades for PPO activation were clarified [11,12]. One consists of HP21 and PAP2/3, while the other HP1, HP6, and PAP1. HP21 activates PAP2 or PAP3; PAP2/PAP3 then converts PPO into active PO. PPO activation can also be catalyzed by the other cascade composed by HP1, HP6, and PAP1. Furthermore, the genes for cSPs and cSP homologues (cSPHs) could be phylogenetically divided into 5 clades: A, B, C, D and E [11,13]. With the exception of CLIPA and CLIPE, most members of the other three clades are expected to have protease activity. Currently, some cSPs in CLIPB, including PPO-activating proteases (PAP1−3) in *M*. *sexta* and SPE in *T*. *molitor*, are thought to directly cleave PPO. In addition, CLIPCs have been shown to cleave CLIPBs, for instance, *M*. *sexta* HP6 activates *M*. *sexta* proPAP1 [11] and *T*. *molitor* SPE activates *T*. *molitor* proSPE [14,15]. However, functions of CLIPDs are poorly characterized, only one member, *M*. *sexta* HP1, was recently described to cleave a CLIPC, *M*. *sexta* proHP6 [12]. Apart from that, cSPs are strictly regulated by serpins [8] and serpins are a superfamily of proteins, most of which inhibit serine proteases. Containing 350–400 residues typically, serpins have a reactive center loop (RCL) near their C-termini, which acts as the binding site for its target protease. After binding, the serpin is cleaved between the P1 and P1’ residues within the RCL, undergoes a dramatically conformation change, and forms a covalent complex with the protease [16]. In *H*. *armigera*, our previous immunotranscriptome study revealed a repertoire of immunity-related genes well conserved in holometabolous insects [17]. Like *M*. *sexta*, two PPO transcripts were identified in the *H*. *armigera* transcriptome. We also found 11 cSPs and 22 serpins, similar to other lepidopteran insects. These results indicate that *H*. *armigera* contains a melanization regulatory system similar to other lepidopteran insects. This established the basis for further studies of serpins regulating the SP cascade in response to viral infection.

Baculoviruses are large DNA viruses that infect insects, primarily the lepidopteran insects. Studies on the interaction between baculoviruses and insect host innate immunity are mainly performed in cell lines. Two cellular mechanisms, RNAi [18] and apoptosis [19,20], are implicated in defense against baculoviruses. However, there are a few studies suggesting that melanization plays a role in defense against baculoviruses in larval insects. It has been demonstrated that melanization can inhibit baculovirus infection in the resistant strain of *H*. *zea* [21,22]. In addition, hemolymph of *Heliothis virescens* is virucidal to baculovirus [23]. More interestingly, the melanin precursor 5, 6-dihydroxyindole (DHI) shows antiviral activity against baculovirus [24]. However, the mechanism by which baculovirus overcomes the host immune system is unclear.

The complete genome of HearNPV was sequenced [25], and multiple viral genes contributing to viral infection have been characterized [26–28]. In our previous study, the HearNPV infection suppressed host defense response [29]. Here, we deciphered molecular mechanisms on how HearNPV modulates host immunity. Quantitative proteomics analyses revealed that the melanization pathway is suppressed in *H*. *armigera* infected with HearNPV. In addition, serpin-5 and serpin-9 induced by HearNPV infection downregulate melanization by inhibiting target proteases, cSP4 and cSP6. Our results suggest that the virus has evolved a novel strategy to suppress the host immune system.

## Results

### Quantitative proteomics identified hemolymph proteins in response to baculovirus infection

**Fig 1:**
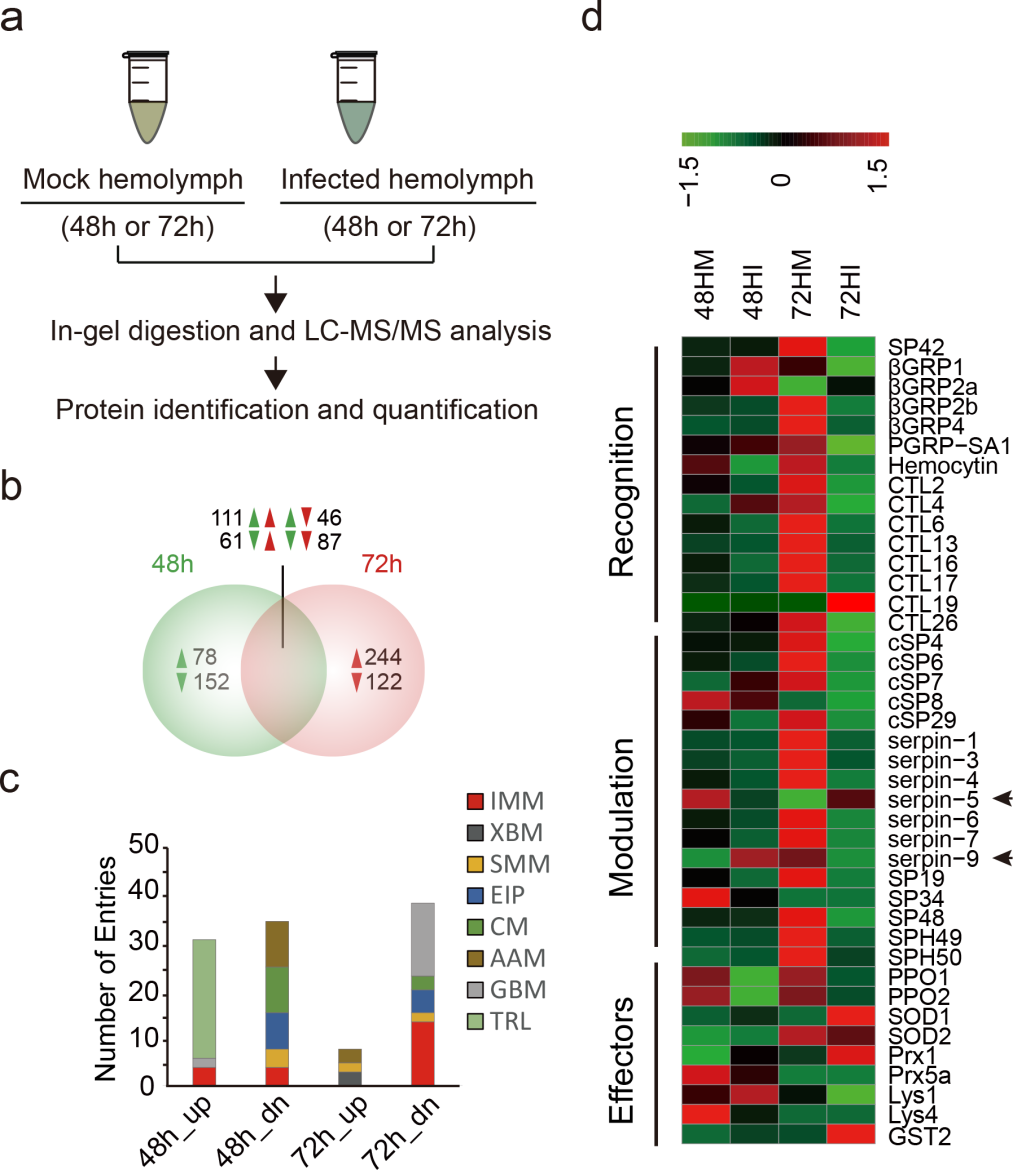
Quantitative proteomics for identification of baculovirus-regulated proteins in hemolymph. (**a**) Quantitative proteomics workflow. (**b**) Venn diagram analysis of identified differential proteins at two time points post infection (48 and 72 hpi). Induction was indicated by upward triangles refers to higher expression in infected versus uninfected hemolymph, and repression was indicated by downward triangles. (**c**) KEGG functional classification of baculovirus-regulated proteins. Functional group abbreviations: IMM, immunity-related; XBM, xenobiotic biodegradation and metabolism; SMM, small molecular metabolism; EIP, environment information processing; CM, carbohydrate metabolism; AAM, amino acids metabolism; GBM, glycan biosynthesis and metabolism; TRL, translation. (**d**) Hierarchical clustering of differentially expressed immunity-related proteins after viral infection.

Our previous study indicated that HearNPV infection dramatically regulated gene expression at 48 and 72 h post ingestion (hpi) in the fat body, the tissue responsible for the synthesis and secretion of most hemolymph polypeptides [29]. Here, we used a proteomic method to examine and quantify hemolymph proteins of *H*. *armigera* infected by HearNPV (Fig 1A). The hemolymph samples (48hM, 48hI, 72hM and 72hI) were collected from control and infected larvae at 48 and 72 hpi, separated by sodium dodecyl sulfate polyacrylamide gel electrophoresis (SDS-PAGE) (S1A Fig), and subjected to liquid chromatography tandem mass spectrometry (LC-MS/MS) analysis. A high quality, non-redundant protein database (HarmgeraNPV_001.fasta) was queried with MS/MS data for protein identification and quantification. We identified a total of 1,028 unique host proteins with the strict criteria that a protein must be identified at least twice, not only in technical repeats but also in biological repeats. We identified more unique proteins than in previous reports, for example, 654 in *M*. *sexta* [30], 752 in *Bombyx mori* [31], and 725 in *D*. *melanogaster* [32], indicating a significant increase in coverage of the insect hemolymph proteome. The pairwise Pearson coefficients of the 1,028 proteins demonstrate excellent data consistency within the control (0.94−1.00) or infected (0.94−0.99) (S1 Table). The control-infected coefficients were between 0.68 and 0.84 at 48 hpi and were between 0.41 and 0.59 at 72 hpi, suggesting that protein levels dramatically changed between the control and infected hemolymph and the differences in protein level increased over time. Consistent with the information revealed by the correlation analyses, principal component analysis (PCA) revealed a clear difference between control and infected hemolymph proteins, and the magnitude of difference at 72 hpi increased compared to 48 hpi (S1B Fig). This is also consistent with our transcriptomic analysis [29].

A total of 45 HearNPV proteins were identified in infected hemolymph (S2 Table), 18 of which belonged to late proteins, including P10, FP25K, and P26. By contrast, fewer early proteins (9) were identified such as GP37, ME53, and FGF. In addition, two viral proteins (HA91, HA133) have both early and late promoters. Analysis of the levels of these viral proteins revealed that two early proteins, ME53 and HA121, were restricted to infected hemolymph at 48 hpi. Conversely, in infected hemolymph at 72 hpi, 5 late proteins (ODV-E66, P24, FP25K, Calyx/PEP, and HA66) were exclusively identified and 10 late proteins including cathepsin were much more abundant. Notably, three late proteins, P26, EGT and cathepsin, represented three of the top four most abundant viral proteins in infected hemolymph not only at 48 hpi but also at 72 hpi. These results suggest that oral inoculation of HearNPV successfully established the systemic infection of host larvae.

Then, we performed pairwise comparisons between control and infected groups to identify the differential proteins (S3 Table). Venn diagram analysis revealed that 230 proteins were exclusively regulated in hemolymph at 48 hpi (78 upregulated and 152 downregulated), while another 366 proteins were specifically regulated in hemolymph at 72 hpi (244 upregulated and 122 downregulated) (Fig 1B). A total of 305 proteins were regulated at both 48 and 72 hpi. Considering the different stages of viral infection, we speculate that time-dependent differential regulation strategies might be adopted by the virus. Importantly, the data showed consistent downregulation of 87 proteins at both 48 and 72 hpi and some of these (11) were immunity-related, including pattern recognition receptors (PPRs), SPs, and PPOs. In addition, it should be noted that although many proteins (244) were exclusively increased at 72 hpi, most (200) had no putative signal peptide, while among the exclusively downregulated proteins (122) at 72 hpi, most (85) did have a putative signal peptide. Meanwhile, 78 proteins were exclusively increased at 48 hpi, 41 of them had no putative signal peptide, while among the exclusively downregulated proteins (152) at 48 hpi, 73 of them have no putative signal peptide. The percentage of proteins without putative signal peptide in increased protein at 72 hpi (82.0%) is much higher that at 48 hpi (52.5%). As baculoviruses are liberated from the nuclei of infected cells by lytic release at late stage of viral infection, increased cell lysis after virus challenge might be responsible for the bias.

To avoid bias, we only focused on differential proteins identified in both control and infected hemolymph. Functional classification indicated that a large proportion of downregulated proteins were involved in carbohydrate metabolism (48 hpi), glycan biosynthesis/metabolism (72 hpi), and immunity-related processes (72 hpi) (Fig 1C). Other notable functional groups were translation and amino acids (aa) metabolism (48 hpi). Importantly, most immunity-related proteins were downregulated at 72 hpi, as already implied by transcriptomic data, demonstrating the robustness of our approach.

### The immune response to baculovirus infection in hemolymph and inhibition of hemolymph melanization by viral infection

Immunity-related proteins were significantly enriched among differential proteins after viral infection. Therefore, we mainly focused on immunity-related proteins identified in hemolymph. Previously, 233 immunity-related transcripts in *H*. *armigera* were identified based on homology [17]. Here, 67 immune proteins were further identified in hemolymph of *H*. *armigera*, including recognition molecules (21), modulators (24), signaling proteins (3) and effectors (19) (S1C Fig). Forty members of these immune proteins were differently expressed in hemolymph during viral infection (S4 Table). Hierarchical clustering revealed that many more immune proteins exhibited more remarkable downregulation than upregulation at the protein level in hemolymph at 48 hpi (8 upregulated and 15 downregulated) and 72 hpi (6 up and 30 downregulated) (Fig 1D and S1D Fig), of which 11 members were consistently downregulated at both time points. Six of the 11 downregulated proteins were postulated to be involved in the melanization activation pathway, including PPO1 (9.5-fold at 48 hpi, 2.5-fold at 72 hpi), PPO2 (14.1-fold at 48 hpi, 2.4-fold at 72 hpi), cSP6 (1.4-fold at 48 hpi, 14.7-fold at 72 hpi), cSP8 (1.5-fold at 48 hpi, infinity at 72 hpi) and cSP29 (3.8-fold at 48 hpi, 22.7-fold at 72 hpi). Other putative melanization components also exhibited downregulation at either 48 hpi or 72 hpi, such as SP42 (2.6-fold at 72 hpi), cSP4 (16.9-fold at 72 hpi), and serpin-3 (2.2-fold at 72 hpi). Interestingly, two serpins, serpin-5 and serpin-9, exhibited an inverse regulation pattern. Serpin-9 was upregulated at 48 hpi (3.1-fold) and downregulated at 72 hpi (2.8-fold), whereas serpin-5 was completely different, being downregulated at 48 hpi (2.8-fold) and upregulated at 72 hpi (infinity). Serpin-5 is involved in regulating melanization [33,34], and the function of serpin-9 has not been previously reported. These results suggest that baculovirus infection suppresses hemolymph melanization in *H*. *armigera*.

**Fig 2:**
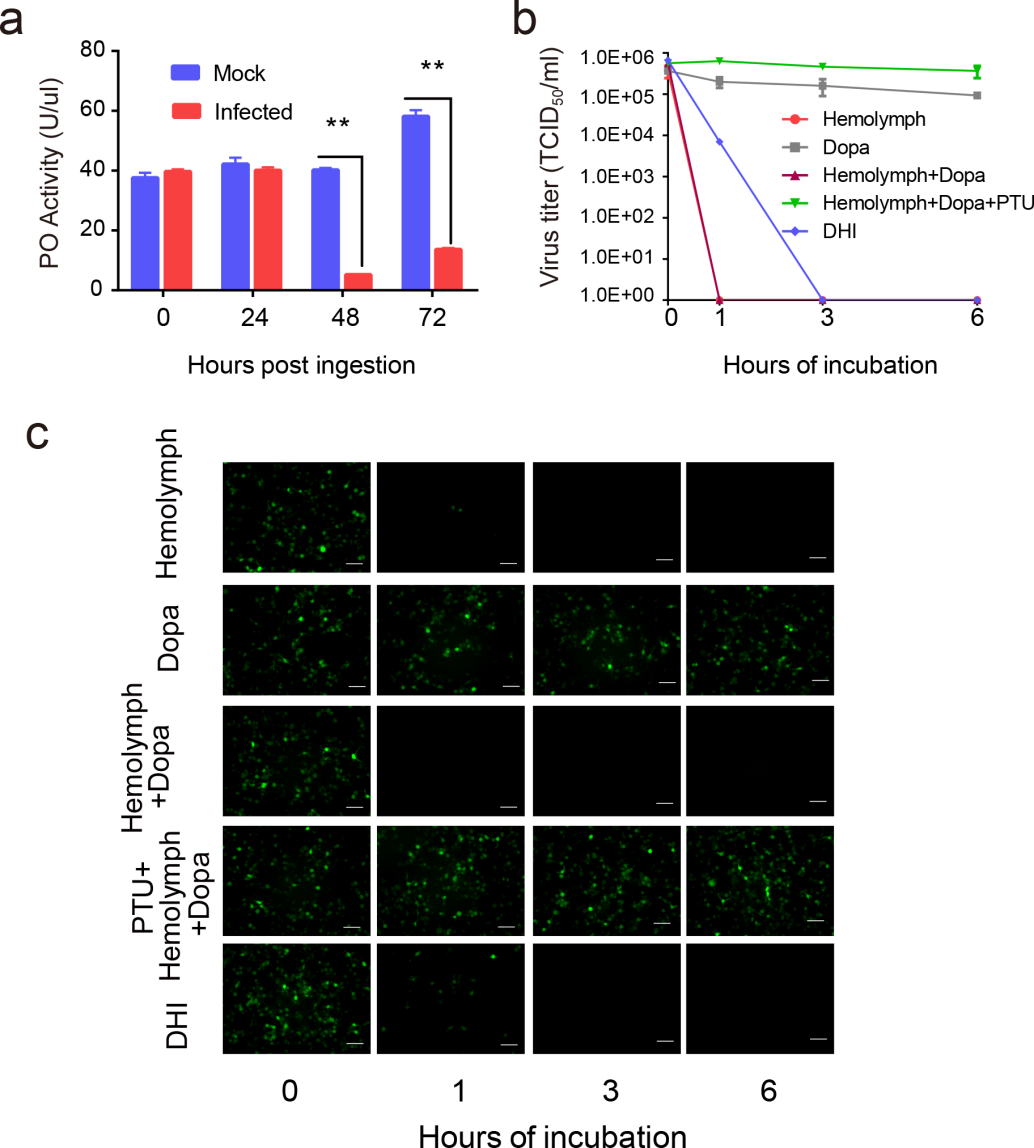
The relationship between baculovirus infection and melanization. (**a**) Comparison of PO activity of 1 μL hemolymph in mock-infected and infected *H*. *armigera* (n = 6) at four time points (0, 24, 48 and 72 hpi). The hemolymph were collected and exposed for 30 min to trigger spontaneous PPO activation. The PO activity levels of infected hemolymph significantly declined at 48 and 72 hpi. Values are expressed as mean ± s.e.m of six independent experiments. Statistical significance was determined using a two-tailed Student’s t-tests. * *p* ≤ 0.05, ** *p* ≤ 0.01. (**b**) The virus titer analysis of baculovirus with different treatments. HearNPV-*egfp* was mixed with either hemolymph, dopa, hemolymph and dopa, hemolymph and dopa with PTU, or DHI. These mixtures were incubated at room temperature for 0, 1, 3, or 6 h, and were quantified for production of infectious BV by end-point dilution assay. Data points represent average titers from triplicate infections. Error bars represent standard errors. (**c**) HearNPV-*egfp*, with different treatments as described above were directly used to infect HzAM1 cells. Fluorescence microscopy images were taken at 72 hpi to observe the viability of baculovirus. Scale bars represent 100 μm.

To test the resultant hypothesis from our quantitative proteomics analysis that baculovirus infection suppresses host melanization, a well-established PO activity assay using *H*. *armigera* larvae hemolymph was performed. Time-course analysis showed that PO activity in infected hemolymph did not exhibit any remarkable changes at 0 or 24 hpi. As infection progressed, PO activity decreased sharply at 48 hpi (7.4-fold) and 72 hpi (4.2-fold) compared to the controls (Fig 2A). The results demonstrated that at the early stage of baculovirus infection, hemolymph melanization was not affected. However, at the middle and late stages of the virus infection, the hemolymph melanization pathway was persistently downregulated.

### Melanization blocks baculovirus infection *in vitro*

Our results showed that baculovirus infection reduced not only PPO amount but also host PO activity. A recombinant variant of HearNPV, HearNPV-*egfp* that expresses green fluorescent protein (GFP) in infected cells, was used in the virus infection assay to evaluate the effects of hemolymph melanization. The HearNPV-*egfp* was incubated with *H*. *armigera* hemolymph and dopa, the substrate of melanization, for 1, 3, and 6 h. Hemolymph only and dopa only were set as controls. Virus titer was determined at different time points by end-point dilution assay (Fig 2B). After 1 h incubation, the virus titer of hemolymph only, hemolymph and dopa group both decreased to zero. However, when phenylthiourea (PTU), specific inhibitor of PO, was incubated together with hemolymph, dopa, and virus, the virus titer did not change even after 6 h incubation. Dopa incubated with virus also did not have significant decrease in virus titer. Moreover, when the HearNPV-*egfp* suspension was incubated with DHI, a reactive intermediate compound generated by PO, the virus titer exhibited 95-fold reduction after 1 h incubation and could hardly be detected after 3 h.

Next, the same samples of reaction mixture for 1, 3, and 6 h were added to HzAM1 cells, after which the fluorescence microscopy images were taken at 72 hpi. When the HearNPV-*egfp* suspension was incubated with DHI, fewer infected cells was observed for 1 h incubation and no infection was observed after 3 h. Consistently, no viral infection was observed in hemolymph and dopa treatment group after 1 h incubation (Fig 2C). However the addition of PTU rescues the infection. Infected cells didn’t decrease even after 6 h incubation. These results demonstrate that melanization can block viral infection *in vitro*.

### Alteration of melanization component protein levels during baculovirus infection

**Fig 3:**
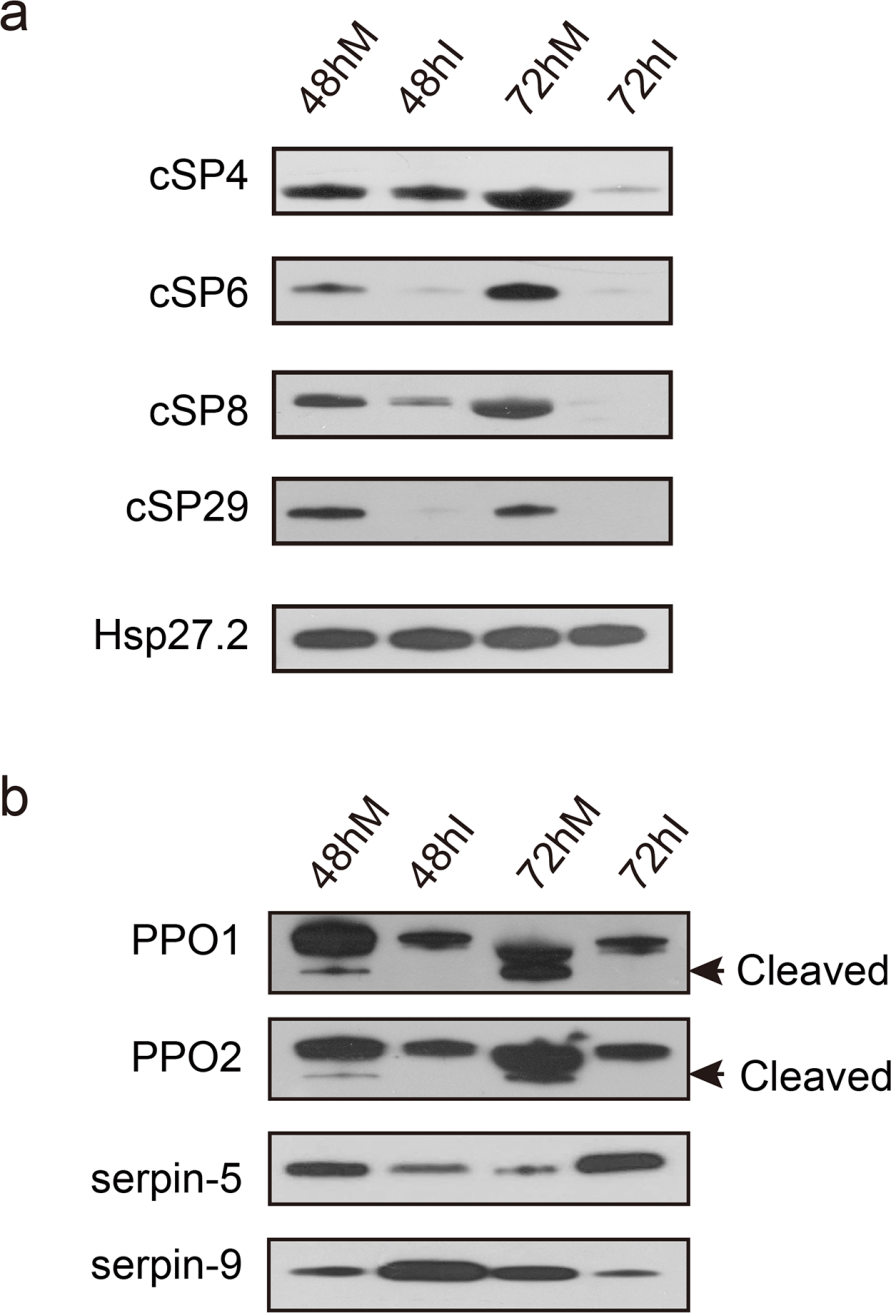
Baculovirus inhibits host melanization at the molecular level. Immunoblot analyses were performed to compare the protein levels of melanization-related components in mock-infected and infected cell-free hemolymph to validate quantitative proteomics data. *H*. *armigera* HSP27.2 (heat shock protein 27.2 kDa) was used as the loading control. (**a**) cSPs involved in the melanization pathway were downregulated in infected hemolymph. (**b**) Serpin-9 and serpin-5 were upregulated sequentially at 48 and 72 hpi, and PPO activation was suppressed.

The abovementioned experimental data suggest a critical role of hemolymph melanization in baculovirus infection. We developed eight antibodies against PPO, cSP, and serpins involved in the melanization pathway (S5 Table) and the specificity of the cSP antibodies were confirmed by a knockdown experiment (S2 Fig). We performed immunoblot analyses of hemolymph from control and infected larvae at 48 and 72 hpi using these antibodies. The samples were left for 30 min for PPO to undergo spontaneous activation. The results showed that the amounts of cSP4, cSP6, cSP8, and cSP29 in *H*. *armigera* hemolymph decreased over the time course in response to HearNPV infection (Fig 3A and S3A Fig). The protein level of cSP4 in infected hemolymph was almost the same as that in mock hemolymph at 48 hpi, but its level was drastically reduced at 72 hpi. Regarding cSP6, the protein bands in the infected group were much weaker than those in the mock group at both 48 and 72 hpi. Moreover, the protein level of cSP8 significantly decreased at 48 hpi, and was undetectable at 72 hpi. Likewise, cSP29 could barely be detected by immunoblot in infected hemolymph (48 and 72 hpi), suggesting the strong effects of viral infection on its expression. In addition, two key enzymes involved in melanization, PPO1 and 2, were significantly downregulated at the protein level upon viral infection. Apart from the changes in protein levels, the cleavage of PPO was inhibited, with no cleaved forms detected in infected hemolymph (Fig 3B), The same result was observed even when increasing 4–7 times higher loading amount (S3B Fig). Furthermore, serpin-9 and serpin-5 were sequentially increased in protein abundance (Fig 3B and S3A Fig); the former was induced at 48 hpi and the latter was induced at 72 hpi, which is consistent with proteomics data, implying that these two molecules play critical roles in the modulation of host melanization and their targets were likely distinct from each other. Taken together, the immunoblot results indicate systemic inhibition of melanization in *H*. *armigera* hemolymph after baculovirus infection.

### Serpin-5 and serpin-9 induced by baculovirus infection inhibit melanization *in vitro*

**Fig 4:**
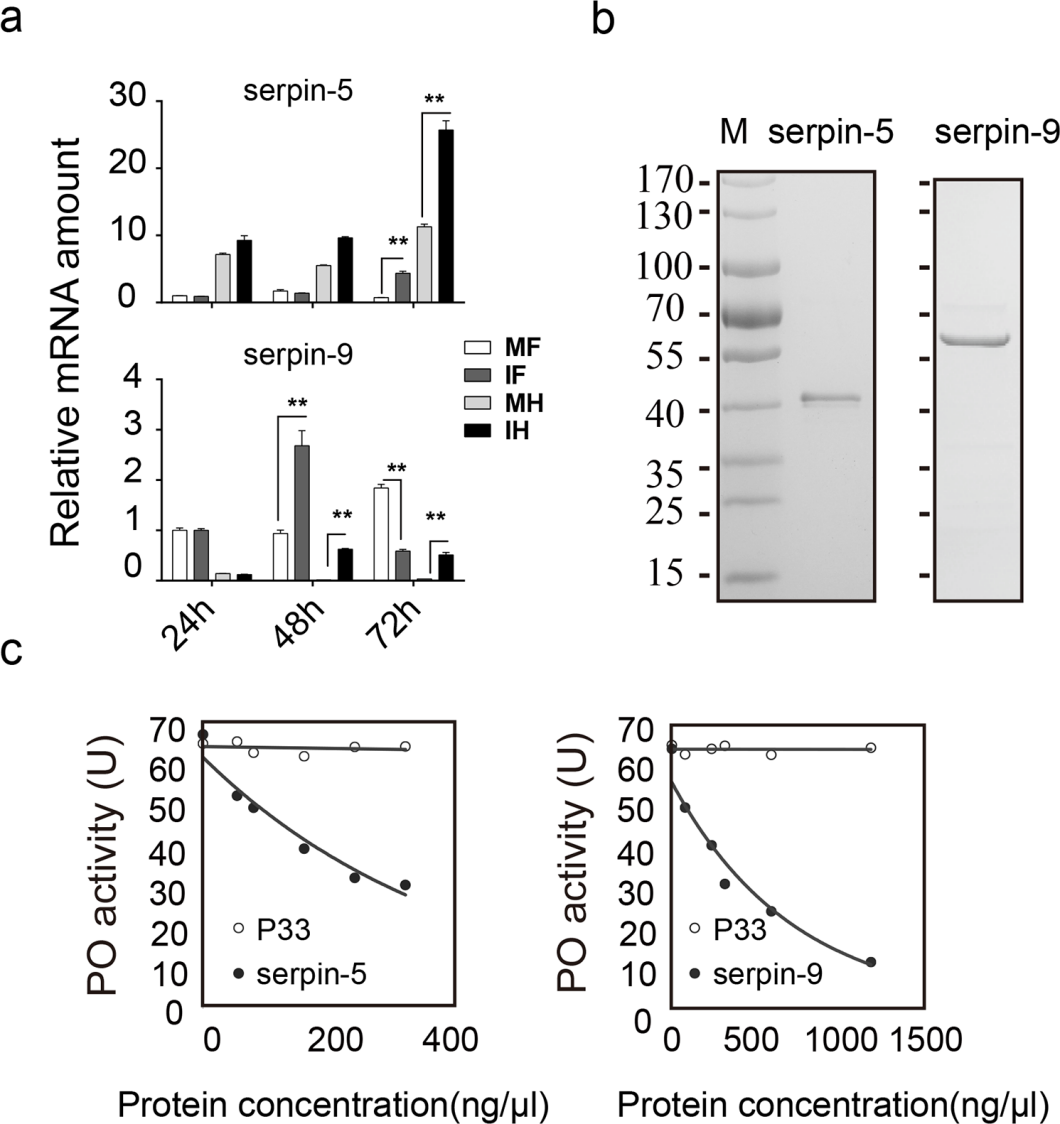
Serpin-5 and serpin-9 inhibit melanization *in vitro*. (**a**) The relative expression levels of serpin-5 and serpin-9 were validated by qPCR analysis at 24, 48 and 72 hpi. Data are present at least three independent experiments (mean and s.e.m). Statistical significance was determined using a two-tailed Student’s t-tests.* *p* ≤ 0.05, ** *p* ≤ 0.01. Sample abbreviations: MF, fat body from mock group; IF, fat body form infection group; MH, hemocytes from mock group; IF, hemocytes form infection group. (**b**) Purification of recombinant serpin-5 and serpin-9 proteins from *E*. *coli*. Purified proteins were resolved by SDS-PAGE. (**c**) Concentration-dependent inhibition of spontaneous melanization by serpin-5 and serpin-9. 1μL hemolymph from naïve 5^th^ instar larvae mixed with different amounts of serpin-5 (0–320 μg/mL) or serpin-9 (0–1200 μg/mL) was incubated at room temperature for 5 min before measuring PO activity. A recombinant baculovirus protein, P33, was used as negative control.

The quantitative proteomic and immunoblot analyses revealed that the protein levels of serpin-9 and serpin-5 were sequentially induced by viral infection, implying their roles in suppression of host melanization. Using quantitative real-time PCR (qPCR), we determined the relative transcriptional levels of *serpin-5* and *serpin-9* in fat body and hemocytes at three time points (24, 48, and 72 hpi), and found that the transcript levels of *serpin-5* and *serpin-9* were substantially induced by viral infection (Fig 4A). Notably, there was no significant difference in *serpin-5* mRNA levels between the control and infected groups at 24 and 48 hpi, but it was significantly induced at 72 hpi in both fat body (>5-fold) and hemocytes (>2-fold). However, *serpin-9* mRNA was strongly elevated at both 48 and 72 hpi (>10-fold) in hemocytes, while it was induced at 48hpi (>2-fold) and suppressed at 72 hpi (>2-fold) in fat body. These qPCR results were consistent with the induction of serpin-5 and serpin-9 at protein level in the infected hemolymph.

To explore the effects of serpin-5 and serpin-9 on PO activity, we performed a PO activity assay *in vitro* using recombinant serpins. First, we cloned the cDNA of serpin-5 and serpin-9 from *H*. *armigera*. *Ha*serpin-5 cDNA encoded a polypeptide of 400 aa residues with a predicted signal peptide of 21 residues. The calculated molecular weight of mature serpin-5 is 42 kDa. *Ha*serpin-9 cDNA encoded a polypeptide of 406 aa residues, with a predicted signal peptide of 20 residues. The calculated molecular weight of mature serpin-9 is 43 kDa. Sequence analysis indicated that *Ha*serpin-5 is orthologous to *M*. *sexta* serpin-5 [33,35] with 78% identity in aa sequence, suggesting that they might share similar regulatory functions. However, the ortholog gene of *Ha*serpin-9 was not found in *M*. *sexta*. Interestingly, phylogenetic analysis revealed that *Ha*serpin-5 and *Ha*serpin-9 form a group with *Ms*serpin-4, *Ms*serpin-5 and *Dm*serpin77Ba [36] (S4A Fig). In addition, comparison of the RCL regions also showed that these five serpins have similar predicated residues at the P1-P1’ positions of the scissile bond (S4B Fig), implying that *Ha*serpin-9 might also participate in melanization.

To explore their biochemical functions, serpin-5 and serpin-9 gene were cloned into pET-28a vector and pET-32a vector respectively, and expressed in an *Escherichia coli* expression system. Soluble serpin-5 and serpin-9 protein were further purified by nickel affinity chromatography. Recombinant serpin-5 and serpin-9 proteins migrated as 42.8-kDa and 61.5-kDa single bands on SDS-PAGE under reduced conditions (Fig 4B) and were confirmed by mass spectrometry.

As melanization was suppressed by HearNPV infection and serpin-5 and serpin-9 were upregulated in hemolymph from baculovirus infected larvae, it’s presumable that serpin-5 and serpin-9 might inhibit melanization in hemolymph. Therefore, we collected hemolymph from naïve larvae of *H*. *armigera* and added serpin-5 and serpin-9 at different concentrations. Before subject to PO activity assay, these samples were exposed to air at 27°C for 5 min to undergo spontaneous melanization. The results showed that PO activities decreased by up to 46% and 83%, respectively (Fig 4C), suggesting that serpin-5 and serpin-9 inhibit melanization by targeting one or more cSPs in the PPO activation cascade.

### Interactions between serpins and their target proteases

**Fig 5:**
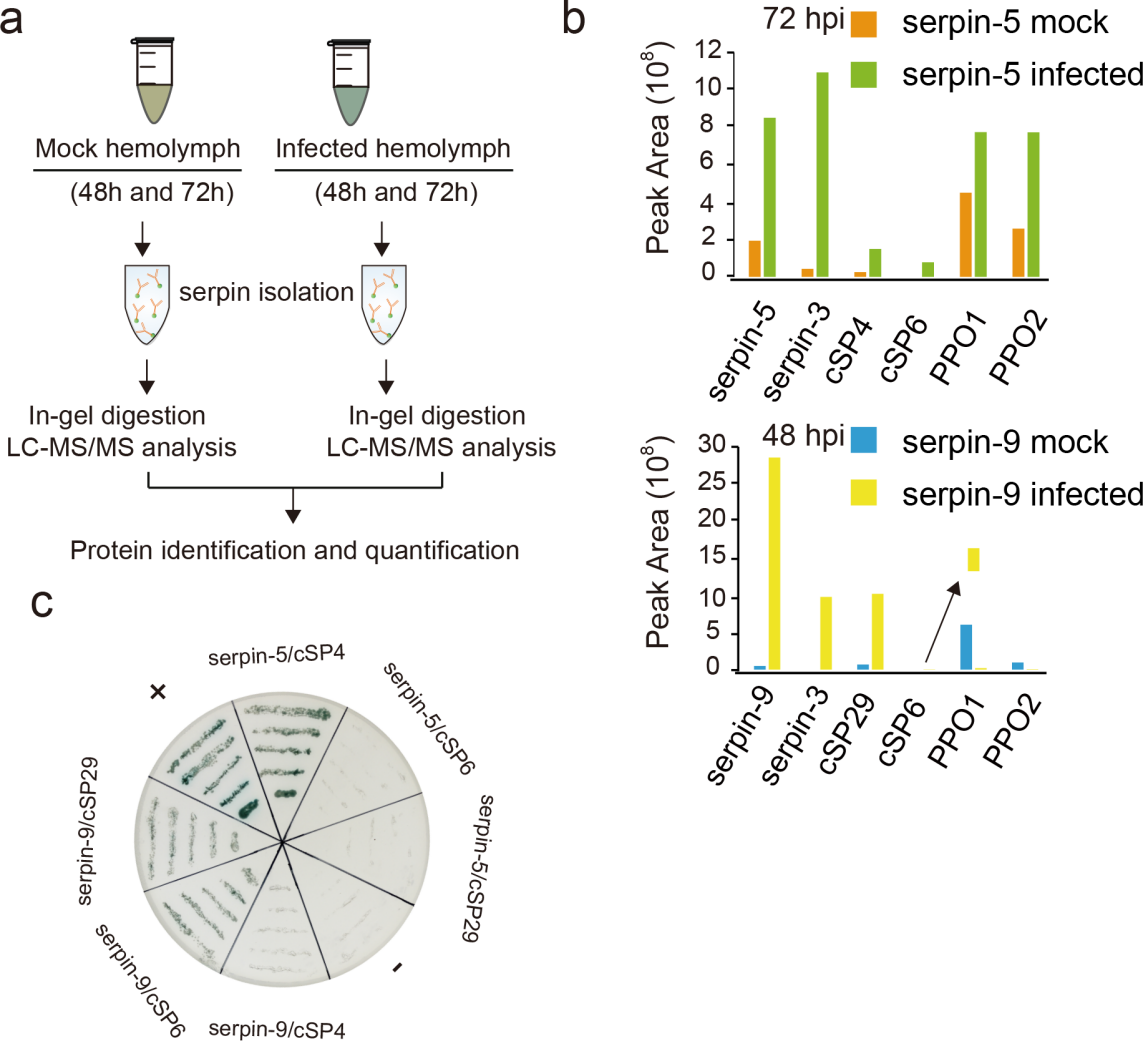
Proteomics analysis of serpin-associated proteins. (**a**) Schematic for isolation of serpin-associated proteins from cell-free hemolymph. (**b**) Proteins eluted from immunoaffinity columns were identified by in-gel trypsin digestion and LC-MS/MS analysis. The arrow point to magnified image of cSP6 peak area. Floating yellow bar indicate the magnified peak area of cSP6. (**c**) Yeast-two-hybrid assay validates the binding between the identified serpins and cSPs in SD-Leu-Trp-His medium with X-α-gal. +: positive control, -: negative control.

The above results showed that serpin-5 and serpin-9 function as inhibitors of PPO activation. To further identify their target cSPs, we tried to activate the infected larval hemolymph at the two time points by exposure to air, and captured serpin-associating cSPs in the hemolymph samples using a semi-quantitative proteomic approach (Fig 5A). Immunoaffinity chromatography using serpin-5 antibody allowed us to isolate complexes formed by serpin-5 and its associated proteins from the hemolymph at 72 hpi. Similarly, serpin-9 and its associated proteins from the hemolymph at 48 hpi were purified using serpin-9 antibody. Proteins eluted from the column were analyzed and quantified using tryptic peptide nano-LC-MS/MS analysis. The abundances of serpin-5 and serpin-9 increased in infected hemolymph (Fig 5B), as suggested by the quantitative proteomic (Fig 1D) and immunoblot analysis (Fig 3D). Among serpin-5 immunoprecipitated proteins (277), cSP4 and cSP6 were identified. Meanwhile cSP29 and cSP6 were identified among serpin-9 immunoprecipitated proteins (214). cSPs were remarkably increased in the infected hemolymph. Phylogenetic analysis revealed that cSP6 as well as cSP8 belong to the CLIPB group and are orthologous to PAP3 and PAP1 in *M*. *sexta*, respectively. Meanwhile cSP4 and its ortholog *M*. *sexta* HP6 belong to the CLIPC group (S5 Fig). Interestingly, cSP29 is orthologous to *M*. *sexta* HP1, which is reported to activate HP6 in *M*. *sexta* and is the only member of the CLIPD group with a known function. Apart from serpins and the target cSPs, serpin-3 and PPOs also tightly associated with the antibody columns. Serpin-3 had similar change patterns to serpin-5 at 72 hpi and serpin-9 at 48 hpi, respectively. The protein abundance of PPO associated with serpin-5 increased at 72 hpi. However, the protein level of PPO bound with serpin-9 substantially decreased. These results indicate that serpin-5 might be a key regulator of cSP4 and cSP6, while serpin-9 might be a critical modulator of cSP6 and cSP29 in the melanization cascade.

To further confirm the specific interaction between serpins and associated cSPs, we conducted a yeast two-hybrid assay. We constructed prey plasmids containing serpin-5 or serpin-9, and bait plasmid containing cSP4, cSP6, or cSP29. In selective growth media, we observed that serpin-5 bound to cSP4, whereas serpin-9 interacted with cSP6 and cSP29 (Fig 5C). The results suggest that cSP4 is likely the targeted protease of serpin-5 while cSP6 and cSP29 are the targeted proteases of serpin-9.

### Inhibition of cSP4 by serpin-5 and cSP6 by serpin-9

**Fig 6:**
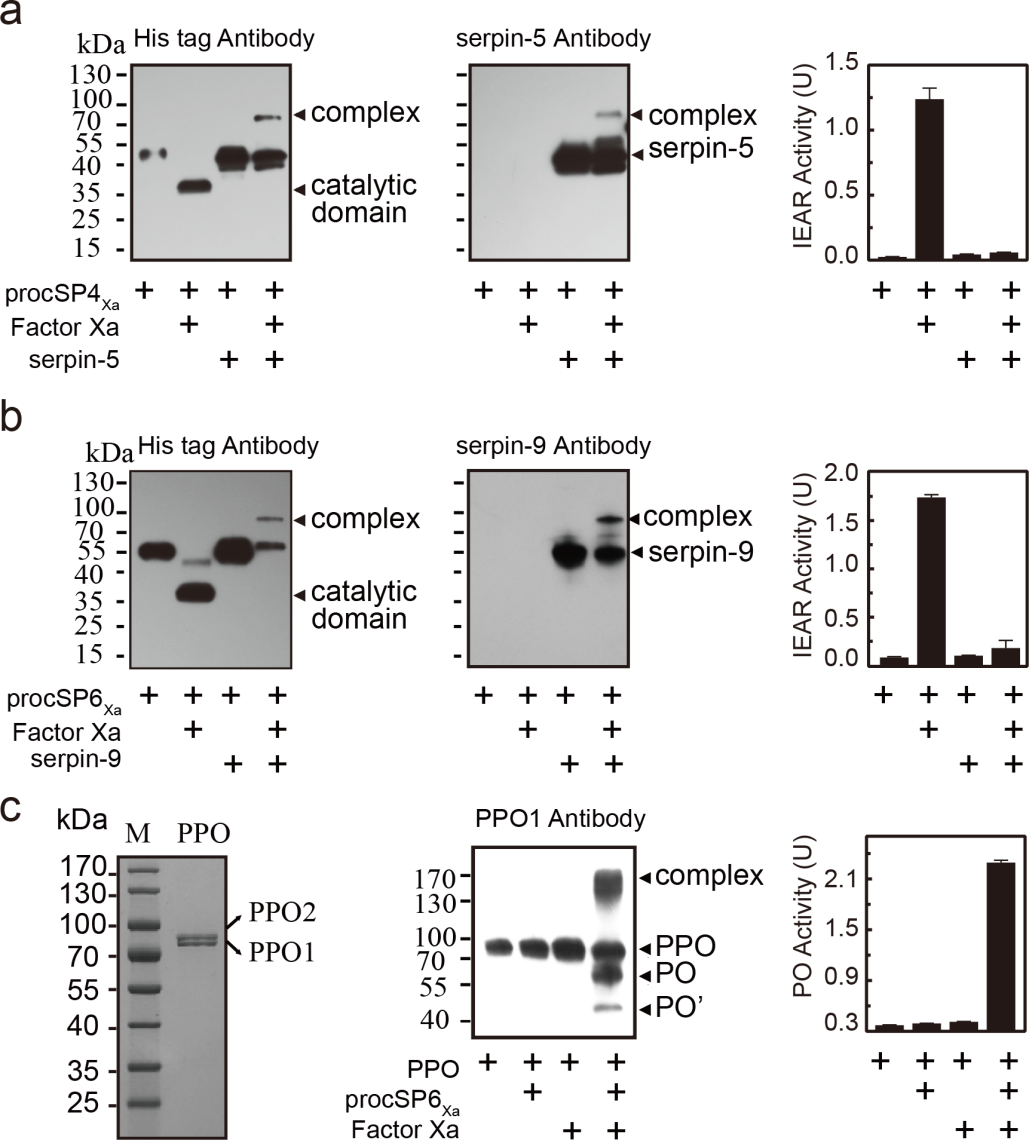
Serpin-5 and serpin-9 inhibit cSP4 and cSP6 activity to prevent PPO activation. (**a**) SDS-stable complex formation between serpin-5 and cSP4_Xa_. 50 ng procSP4_Xa_ was activated by 400 ng factor Xa and then incubated at room temperature for 10 min with serpin-5 at a molar ratio of 5:1 (serpin-5:cSP4_Xa_). In control reactions, cSP4_Xa_ or factor Xa were omitted. The samples were subjected to immunoblot analysis using antibody against His-tag (left panel) or serpin (middle panel). The serpin-cSP4_Xa_ complex bands are marked by arrowheads. In the immunoblot analysis using antibodies against His-tag, the amount of loading sample in the second lane was six times the expected amount to visualize the band. Amidase activity of the mixtures was measured using IEAR substrate at 405 nm (right panel). (**b**) SDS-stable complex formation between serpin-9 and cSP6_Xa_. (**c**) 5 μg purified hemolymph PPO from naïve 5^th^ instar larvae was subjected to SDS-PAGE (left panel) and then purified hemolymph PPO was activated by cSP6_Xa_. 50 ng procSP6_Xa_ was activated by factor Xa, and then incubated with 100 ng PPO at room temperature for 10 min. The mixture was subjected to immunoblot analysis using PPO1 antibody (middle panel) or used to measure PO activity (right panel). All data are represented as mean ± s.e.m of three independent experiments.

To further investigate the interaction of serpin-5 with cSP4 and serpin-9 with cSP6, we expressed cSP4 and cSP6 in their zymogen forms using the *Drosophila* S2 expression system. Predicted activation site of cSP4 and cSP6 was manually changed from LDLH^92^ to IEGR^92^ and VGNK^156^ to IEGR^156^ respectively. IEGR is the sequence of cleavage, which could be recognized by bovine coagulation factor Xa. They were named as procSP4_Xa_ and procSP6_Xa_. Expressed recombinant proteins were purified by Ni-NTA agarose column. The amidase activity was detected using the substrate acetyl-Ile-Glu-Ala-Arg-*p*-nitroanilide (IEAR). Serpin-5 that was incubated with factor Xa-activated cSP4_Xa_ was found to form a higher molecular weight complex by immunoblot (Fig 6A). Once being activated by factor Xa, the cSP4_Xa_ zymogen band disappeared, and the catalytic domain of cSP4_Xa_ band was detected by anti-His antibodies. When serpin-5 was mixed with active cSP4_Xa_, a novel band with high molecular weight appeared. IEAR activity of cSP4 was inhibited (Fig 6A). Similar results were also observed with serpin-9 and cSP6_Xa_ (Fig 6B), active cSP6_Xa_ was inhibited by serpin-9, as demonstrated by the formation of high molecular complex and decreased IEAR activity. These results confirm that serpin-5 and serpin-9 can inhibit cSP4 and cSP6 enzymatic activity by forming an SDS-stable complex respectively. Because cSP6 is predicted to function as PAP to cleave PPO directly, the inhibition of cSP6 by serpin-9 would lead to suppression of PPO activation. To test the hypothesis, hemolymph PPOs was purified from hemolymph of *H*. *armigera* and shown to be a heterodimer formed by PPO1 and 2, which is consistent with results revealed from *M*. *sexta* and *H*. *diomphalia* (Fig 6C and S7A Fig). It exhibited PO activity after activation by alcohol in native PAGE (S7B Fig). Then, we incubated purified PPO with cSP6_Xa_ with or without factor Xa. The results clearly showed that PPOs were enzymatically cleaved and activated by cSP6_Xa_ (Fig 6C and S7C Fig). Taken together, serpin-5 and serpin-9 suppress PPO activation by the inhibition of cSPs in melanization cascade.

### Serpin-5- and serpin-9-depleted *H*. *armigera* increased survival to baculovirus infection

**Fig 7:**
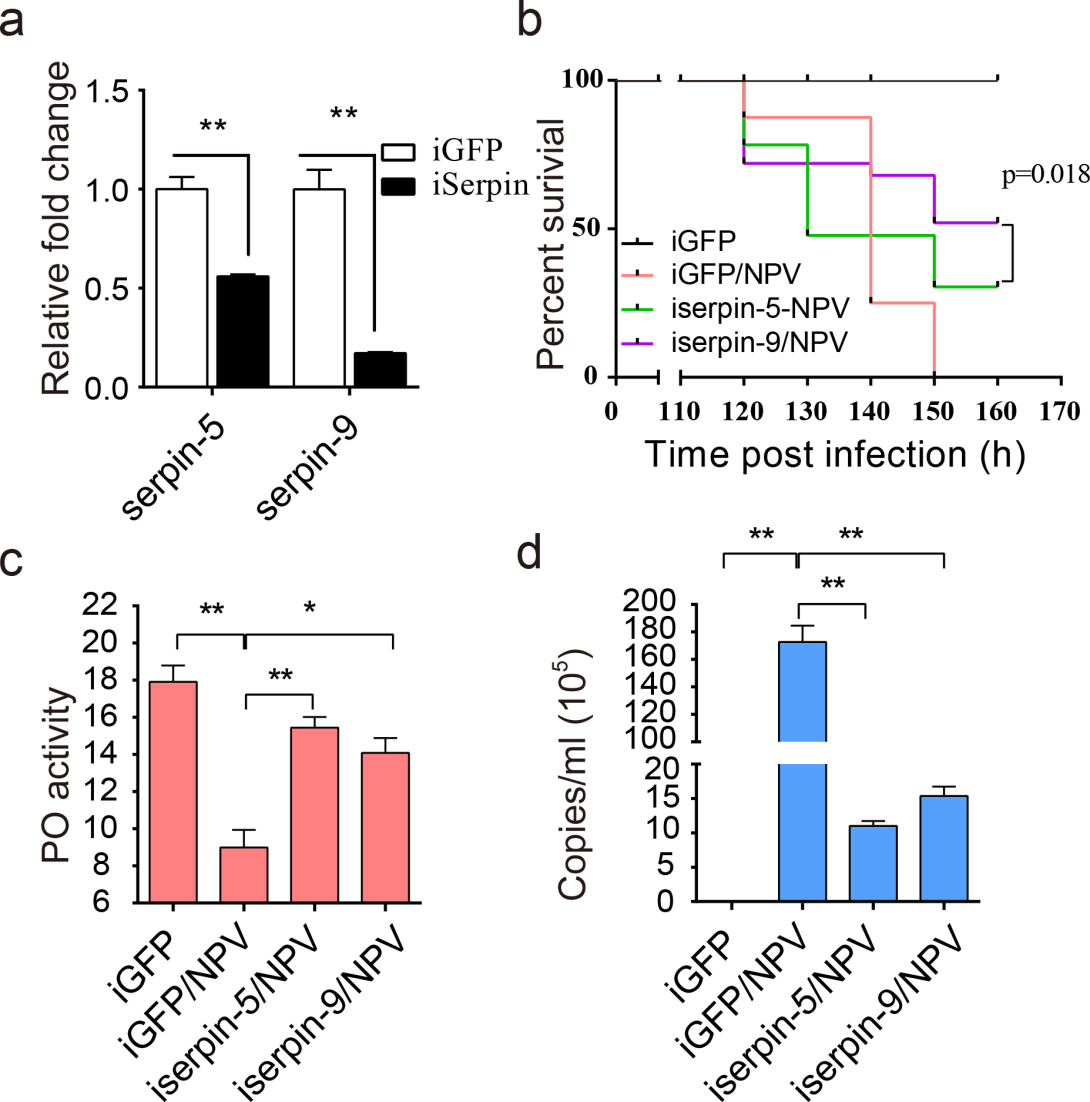
Effects of serpin-5 and serpin-9 knockdown on the mortality of *H*. *armigera* larvae. (**a**) The expression levels of serpin-5 or serpin-9 after RNAi was measured by qPCR. (**b**) The survival rate of *H*. *armigera* showed that the depletion of serpin-5 or serpin-9 enhanced the resistance of *H*. *armigera* to HearNPV infection. The survival rate of iserpin-5/NPV or iserpin-9/NPV *H*. *armigera* was significantly different from that of the iGFP and iGFP/NPV *H*. *armigera* (*p* ≤ 0.01). Each experiment was performed in three replicates. (**c**) The PO activity was upregulated in iserpin-5 and iserpin-9 larvae compared to iGFP larvae after HearNPV infection. (**d**) Viral DNAs from hemolymph were extracted, and qPCR was performed to determine genomic DNA copies based on a standard curve. The viral genomic DNA copies was downregulated in iserpin-5 and iserpin-9 larvae compared to iGFP larvae after HearNPV infection. Data were shown as mean ± s.e.m of three independent experiments. Statistical significance was determined using a two-tailed Student’s t-tests. * *p* ≤ 0.05, ** *p* ≤ 0.01.

To reveal the effect of serpin-5 and serpin-9 *in vivo* during baculovirus infection, we conducted dsRNA mediated knockdown by injecting double-stranded RNA (dsRNA) of serpin-5 or serpin-9 into 2^nd^ instar larvae of *H*. *armigera*. The relative mRNA expression levels were detected by qPCR in whole larvae, and the protein levels were detected by immunoblot in hemolymph. The results showed that the transcript level of serpin-5 and serpin-9 were decrease by 43% or 83% in larvae injected with dsserpin-5 or dsserpin-9 respectively (Fig 7A). Immunoblotting tests also confirmed efficiency of serpin-5 and serpin-9 RNAi (S6A Fig). When injected with dsserpin-5 or dsserpin-9 before HearNPV treatment, the survival of *H*. *armigera* increased due to the knockdown of serpin-5 or serpin-9 (Fig 7B). Then we collected hemolymph and measured PO activity. We found that depletion of serpin-5 and serpin-9 significantly increased PO activity compared to the control after HearNPV infection (Fig 7C). In addition, copies of the viral genomic DNA in hemolymph was measured by qPCR. The results showed that the number of viral DNA copies dramatically decreased by 93.6% or 91.1% in hemolymph of serpin-5 and serpin-9 depleted larvae compared to the control (iGFP-NPV) (Fig 7D). Consistently, immunoblot indicated that the amount of VP39, the structural protein of HearNPV, dramatically decreased in hemolymph of serpin-5 and serpin-9 depleted larvae in comparison to the control (iGFP-NPV). Conversely, PPO2, cSP6, cSP29 was apparently increased in hemolymph of serpin-5 and serpin-9 depleted larvae, while cSP4 increase in hemolymph of serpin-5 depleted larvae (S6B Fig). Clearly, the decline of serpin-5 or serpin-9 in *H*. *armigera* increased PO activity, resulting in elevated resistance against baculovirus infection.

## Discussion

After viral infection, it appeared that cathepsin, EGT and P26 represented three of the top four most abundant viral proteins in infected hemolymph not only at 48 hpi but also at 72 hpi. These are secreted proteins, and are not included in the top 20 most abundant viral proteins in infected fat body at 72 hpi [29]. Cathepsin [37], an enzyme involved in insect disintegration, increased over time during infection. EGT was reported to block insect molting and pupation [38], as observed in our previous study [29]. P26 was described as a budded virus (BV) specific structural protein, the high level of P26 suggested that a large number of BV virions were secreted into hemolymph of infected *H*. *armigera* at 48 and 72 hpi. There were high abundance of virus proteins in the hemolymph, however, our proteomic study also indicates that most immunity-related proteins were downregulated, including the melanization pathway, an essential component of insect immune response. Baculovirus encoded microRNA may be employed to regulate the host immunity-related genes [39,40], and increased serpin-5 and serpin-9 may also be involved in the host immunity-related gene expression (S6B Fig).

Because baculoviruses are natural enemies of insects, specific interactions between the virus and host likely influence virus survival. Our previous report revealed that RNAi and JAK-STAT pathways were not significantly regulated during baculovirus infection [29]. In this study, our proteomic analysis show that levels of most AMPs were not significantly changed. But most of melanization components were downregulated in hemolymph after viral infection. Further results show that melanization can inactivate baculoviruses and block their infection. The fact that hemolymph does not melanize during infection by baculovirus in permissive lepidopteran hosts but melanizes in resistant ones has been reported [21]. The resistance is attributed to the impact of melanization in hemolymph, however, the inhibition of melanization in permissive hosts is still elusive. In this study, our proteomics and immunoblot analyses demonstrated that the protein level of melanization-activating components exhibited a significant decline during the viral infection, while two putative negative regulators of melanization, serpin-5 and serpin-9, increased instead. Consistent with the change at the protein level of melanization genes, PPO activation was inhibited and PO activity in the infected hemolymph dramatically decreased. Hemolymph PO of the larval tobacco budworm, *H*. *virescens*, is virucidal to baculovirus [23], and DHI generated by PO has virucidal activity against baculovirus AcMNPV [24]. In this study, similar effects of melanization on HearNPV were observed in *H*. *armigera*, hence, we concluded that the permissiveness of baculovirus infection is attributable to the inhibition of melanization by regulation of protein levels in hemolymph.

**Fig 8:**
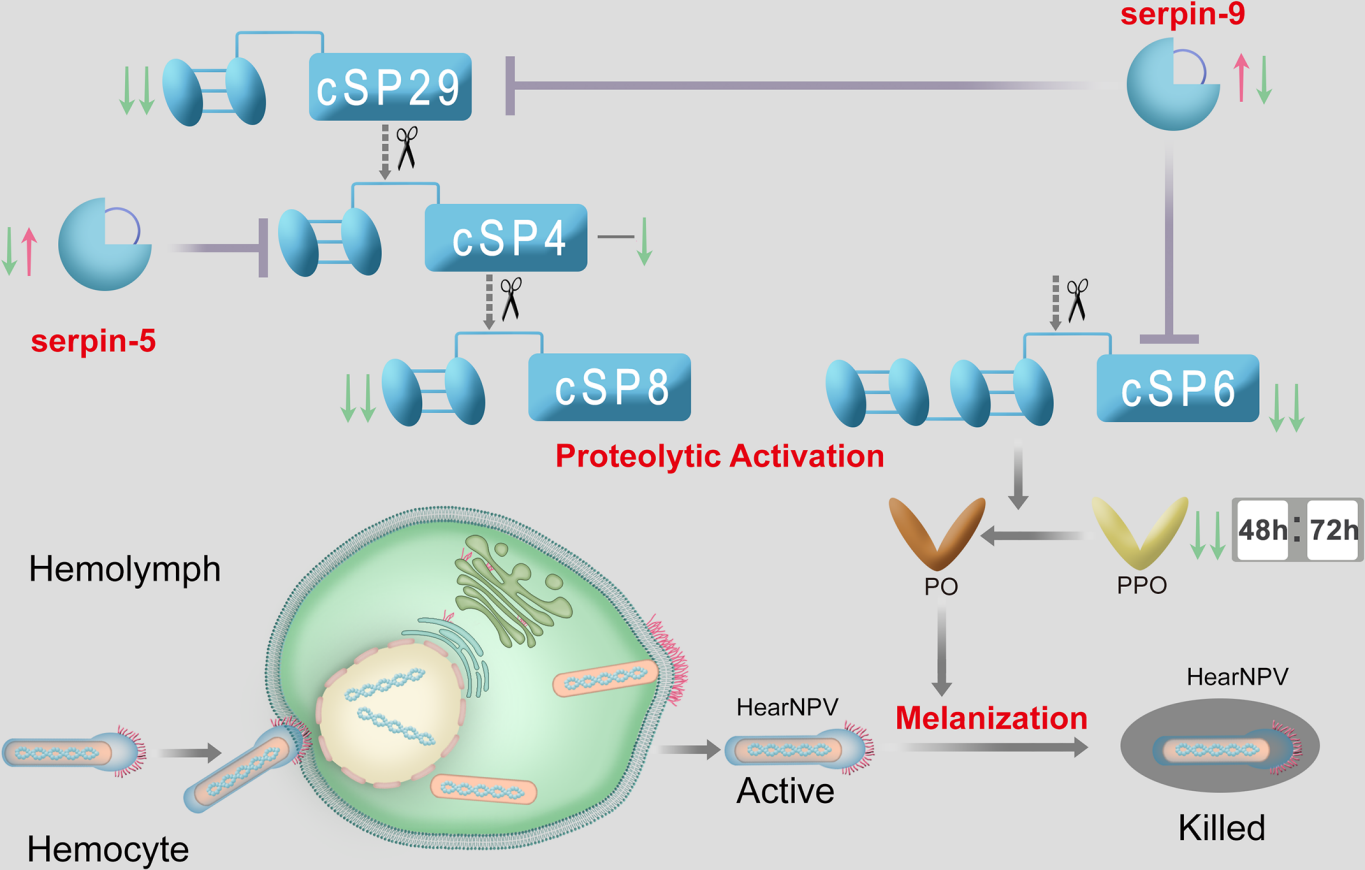
Proposed model for regulation of PPO activation cascade in *H*. *armigera* during baculovirus infection. HearNPV infection results in persistent suppression of melanization in *H*. *armigera* hemolymph, which helps baculovirus particles to modulate the virucidal capacity of melanization in hemolymph. In detail, serpin-5 inhibits cSP4, and serpin-9 inhibits cSP6 and cSP29. PPO can be proteolytically activated by cSP6. During viral infection, the expression of Sepin-9 and serpin-5 are induced sequentially, while among components of the melanization pathway, cSP4 decreases at 72 hpi, and cSP6, cSP29, and PPOs decrease continuously.

Melanization is an important invertebrate immune mechanism. The major components in the melanization pathway such as SPs and serpins have been characterized in some insects and other arthropod species [41]. However, melanization cascade in *H*. *armigera* remains unclear. In our previous work, transcripts for 46 SPs, 22 serpins, 7 SPHs and 2 PPOs were identified in *H*. *armigera* [17], providing fruitful ground for deciphering their roles in melanization. In this study, we identified the proteins encoded by 11 SPs, 10 serpins, 2 PPOs, and 3 SPHs in hemolymph using proteomics. Based on phylogenetic analysis, many were found to have orthologs in other insects, strongly suggesting the conservation of melanization-mediated immunity in the cotton bollworm. We deciphered the mechanism of HearNPV suppressing host melanization reaction using integrated approaches (Fig 8). Phylogenetic analysis reveals that *H*. *armigera* melanization reactions is homologous to those reported in *M*. *sexta*. In *H*. *armigera*, cSP29-cSP4-cSP8 was predicted to form a sequential activation cascade. Both cSP6 and cSP8 are PPO activating enzyme. The cleavage and activation of PPO by cSP6 are further demonstrated by *in vitro* reconstitution (Fig 6C). Moreover, the inhibition of cSP4 activity by serpin-5 was similar to the interaction of HP6 regulated by serpin-5 in *M*. *sexta* [33]. However, we did not find orthologue of *M*. *sexta* PAP-2 in *H*. *armigera* and the inhibition of PPO activation by serpin-9 has not been reported in *M*. *sexta* at present. To the best of our knowledge, this is the first time that melanization pathway is clarified in *H*. *armigera*, and it is shown to respond to viral infection, which further advances our knowledge of melanization in diverse insects and provides a theoretical basis for better controlling agricultural pests.

In our study, serpin-5 and serpin-9 were demonstrated to be involved in modulating the melanization reaction during viral infection. Serpin-5 was previously shown to be induced in hemolymph of *M*. *sexta* after bacterial or fungal infection and to inhibit melanization [35]. Similar results were also found in *B*. *mori* [34]. In this study, we demonstrated that the inhibition of melanization caused by serpin-5 is indispensable to viral infection. Strikingly, we identify a novel serpin, serpin-9, whose ortholog in the model insect *M*. *sexta* has not been found, but that also participates in the suppression of melanization for successful viral infection. The inhibitory mechanisms of these two serpins are different. Serpin-9 acts upstream and downstream to serpin-5, and its induction time is also earlier (48 vs. 72 hpi). In addition, it is also possible that serpin-5 and serpin-9 might inhibit melanization by regulating a signaling pathway (such as Toll pathway) that controls the expression of components of melanization cascade. As the increased expression of components of the melanization cascade in both iserpin-5/NPV and iserpin-9/NPV insects were observed (S6B Fig). Interestingly, a baculovirus encoded serpin has been characterized as an inhibitor of host melanization and its expression increased the virulence of the virus by four-fold in *Trichoplusia ni* larvae [42]. However, this molecule is not an ortholog of serpin-5 or serpin-9 in *H*. *armigera*, and its exact molecular mechanism remains unknown.

We have demonstrated that the polyphagous pest *H*. *armigera* retains very complex melanization against the invasion of microbes. However, in nature, pathogens have evolved distinct strategies to modulate host immunity by suppressing melanization. An antibiotic produced by the entomopathogenic bacterium *Photorhabdus luminescens* inhibits melanization by targeting PO activity in *M*. *sexta* [43]. Parasitoid wasps associated polydnavirus also expresses two viral proteins, Egf1.0 and Egf1.5, to suppress melanization via PAP inhibiton [44–46]. More recently, it was reported that a viral protein encoded by WSSV interacts with PAP to suppress melanization in the shrimp *Penaeus monodon* [10]. Different from these findings, HearNPVs exploit host serpins to inhibit SPs at different steps of the melanization cascade. The phylogenetic analysis comparing the SPs interacting with serpin-5 or serpin-9 to other insect SPs showed that they belong to three different clades. cSP6 belongs to the CLIPB clade and is an ortholog of *M*. *sexta* PAP3. Indeed, cSP6 is inhibited by serpin-9 and acts as a PAP in *H*. *armigera* and is found to cleave PPO directly. cSP4, which interacts with serpin-5, is predicted to cleave PAP. cSP4 belongs to the CLIPC clade and is the *Helicoverpa* ortholog of *Ms*HP6. *Ms*HP6 has been verified experimentally to cleave *Ms*PAP1 (a CLIPB member), while biochemical studies have shown that *Ms*PAP3 cleaved PPO directly in *M*. *sexta* [47]. Moreover, cSP29, targeted by serpin-9, is an SP belonging to the CLIPD subfamily, and its hypothesized function is to cleave cSP4. More recently, *Ms*HP1 was identified to be able to cleave *Ms*HP6 and its activation did not require proteolytic cleavage [12]. It is tempting to postulate that *Ha*cSP4, cSP6, and cSP29 also function as their orthologs in the melanization cascade. Among them, cSP6 and cSP8 are orthologous to *M*. *sexta* PAP3 and PAP1, respectively and may function as PAPs [47,48]. Likewise, cSP4 is postulated to cleave PAPs [49], while cSP29 is postulated to cleave cSP4.

Taken together, we envision a mechanism whereby baculovirus overcomes host melanization. The viral infection induces the level of serpin-5 and serpin-9 in hemolymph. Serpin-5 specifically inhibits cSP4, and serpin-9 inhibits cSP6 and cSP29, resulting in a dramatic decline of PO activity to suppress the virucidal capacity of host melanization (Fig 8). This mechanism, which involves baculovirus induced serpin-5 and serpin-9 and then inactivates the host immune system, suggests that these two negative regulators of immune response are important for baculovirus infection in insects. These findings improve our understanding of the interaction between HearNPV and its co-evolutionary host *H*. *armigera*.

## Materials and methods

### Insects, cell lines, and viruses

The colony of cotton bollworm *H*. *armigera* was raised in the laboratory as previously described [17]. The *H*. *zea* cell line HzAM1 was maintained at 27°C in Grace’s insect medium supplemented with 10% fetal bovine serum (FBS). The HearNPV G4 strain was used in this study [25]. Occlusion bodies (OBs) were amplified from infected larvae. HearNPV-*egfp* was constructed in a previous work [50].

### Viral infection of *H*. *armigera* larvae

The experiments were performed as described in our previous study [29]. Briefly, 96 day-2 3^rd^ instar *H*. *armigera* larvae were divided into two groups. After 16 h of starvation, each larva in the infection group was fed for 10 min on 2 μL 10% sucrose solution containing edible blue dye contaminated with HearNPV at a final concentration of approximately 6.7×10^7^ OBs/μL (99% lethal concentration, LC_99_), while each larva in the mock group was fed on 2 μL 10% sucrose solution containing edible blue dye alone.

### Sample preparation for mass spectrometry

The cell-free hemolymph samples were prepared as previously described [30]. After virus ingestion, the prolegs of larvae were cut at 48 and 72 hpi, and hemolymph was collected into clean tubes containing 1 mM PTU and 1 mM *p*-aminobenzamidine (pAB). Then the hemolymph was centrifuged at 4,000 ×g at 4°C for 5 min to remove hemocytes. Hemolymph from six infected larvae at 48 hpi was pooled as 48 h infected hemolymph (48hI) and hemolymph from six infected larvae at 72 hpi was used as 72 h infected hemolymph (72hI). Similarly, 48 h mock-infected hemolymph (48hM) and 72 h mock-infected hemolymph (72hM) were prepared from mock-infected larvae. In this study, the sample size of 6 was twice as many as reported in *M*. *sexta* [30]. The experiment was repeated twice to obtain biological replicates. The protein concentrations of the twelve samples were determined using the bicinchoninic acid assay (BCA) method.

Equal amounts of the samples (25 μg) were separated on 4–15% gradient SDS-polyacrylamide gels (Bio-Rad, Hercules, CA, USA) and stained with Coomassie Brilliant Blue G-250 for visualization. Subsequently, the gel lanes were excised into 12 or 6 slices for the 48 h samples and five slices for the 72 h samples. Gel slices were subjected to reduction, alkylation and in-gel digestion with mass spectrometry grade trypsin (Promega, Madison, WI, USA). After digestion, the resulting peptides were extracted by 1% trifluoroacetic acid in 60% acetonitrile, near-dried in a Speedvac, solubilized in 0.1% trifluoroacetic acid in 2% acetonitrile 0.1% trifluoroacetic acid, ultracentrifuged and the supernatant then subjected to LC-MS/MS analysis as previously described [29]. Three technical replicates were performed for each biological sample in the LC-MS/MS analysis.

### Bioinformatics analysis

The MS data analyses were performed using Proteome Discoverer software, supported by a protein database (HarmigeraNPV_001). The database was deduced from the putative open reading frames based on the transcriptome unigene dataset [17] and the HearNPV genome [25]. Protein identification and quantification were performed as previously described [29]. The relative abundance of protein was represented as the peak area of the target protein normalized by the average peak area of two internal reference proteins (angiotensin converting enzyme and laminin β-2 chain). The statistical differences across the samples were analyzed by Student’s t-test. A protein with a fold change ≥ 1.4 and p < 0.05 was considered differentially regulated.

Kyoto Encyclopedia of Genes and Genomes (KEGG) orthology (KO)-based annotation system (KOBAS) and PCA analyses were performed as previously described [29]. Based on the relative abundance of immune proteins, the heatmap package in the R environment was implemented to analyze immune protein abundance in hemolymph. Phylogenetic trees were constructed using MEGA6 with the neighbor-joining method.

### PO activity assay

Cell-free hemolymph samples were collected from mock-infected and infected *H*. *armigera* at 0, 24, 48 and 72 hpi as described above, but without adding PTU or pAB. After incubation at 27°C for 30 min, the PO activity assays were conducted in 96-well plates containing 1 μL hemolymph followed by addition of 200 μL substrate solution (2 mM dopamine in 50 mM sodium phosphate buffer [pH 6.5]). PO activity was determined at 470 nm using a plate reader. One unit of activity was defined as ΔA470 of 0.001 in one minute. To measure PO activity of hemolymph in serpin-5 or serpin-9 depleted larvae, hemolymph samples were incubated at 27°C for 5 min to observe the differences between samples.

Hemolymph from naïve 5^th^ instar larvae was collected into tubes containing recombinant serpin-5 (10 μL, at final concentrations of 0–320 μg/mL) or serpin-9 (10 μL, at final concentrations of 0–1200 μg/mL). A recombinant baculovirus protein, P33, was used as negative control. After incubation for 5 min at room temperature, the reaction mixtures were subjected to the PO activity assay.

### Antibody generation and immunoblot analyses

procSP4, procSP6, procSP8, procSP29, serpin-5, PPO1, or PPO2 cDNA encoding mature protein was amplified and cloned into pET-28a vectors, and P33 and serpin-9 cDNA encoding mature protein was cloned into a pET-32a vector. The cloning primers are presented in S6 Table. These recombinant proteins were expressed in *E*. *coli* BL21 and affinity purified on a Ni-NTA agarose column (Qiagen, Hilden, Germany). Polyclonal antibodies were produced in rabbits against the recombinant proteins (Beijing Protein Innovation, Beijing, China). Cell-free hemolymph samples were collected into clean tubes containing 1 mM PTU and incubated at 27°C for 30 min. Hemolymph samples were resolved on 4–15% gradient SDS-polyacrylamide gels (Bio-Rad) and electrotransferred onto a polyvinylidene difluoride (PVDF) membrane (Invitrogen, Carlsbad, CA, USA). Immunoblot analyses were performed using 1:5000 diluted polyclonal antibodies as the primary antibodies. *H*. *armigera* (heat shock protein 27.2 kDa (HSP27.2) was used as a control for loading and transfer. Band intensities of scanned blots were quantified using ImageJ. The integrated intensity of a fixed area was measured, and background levels were subtracted.

### RNA preparation and qPCR analysis

Total RNA samples were prepared from the fat body, hemocytes, or whole body of *H*. *armigera*. qPCR reactions were performed on the MX3000P system (Stratagene, San Diego, CA) using SYBR green PCR master Mix (Tiangen, Beijing, China). Thermal cycling conditions were: 94°C, for 5 s; 59°C, for 20 s; and 72°C, for 20 s. Quantitative measurements were performed in triplicate and normalized to the internal control ribosomal protein S7 for each sample. Primers used for qPCR are listed in S6 Table.

### Purification of soluble serpin-5 and serpin-9

The recombinant serpin and P33 proteins were induced in *E*. *coli* grown in LB medium (1.6 L) with 0.5 mM isopropyl β-D-1-thiogalactopyranoside at 16°C overnight. The *E*. *coli* cells were harvested by centrifugation at 4,500 rpm for 10 min, resuspended in 60 mL lysis buffer (50 mM Tris-HCl, pH 7.5, 0.3 M NaCl, 5 mM β-mercaptoethanol, 1 mM phenylmethane sulfonyl fluoride [PMSF]), and then incubated with lysozyme (1 mg/mL) for 20 min on ice. The suspensions were further incubated with DNase (10 U/mL) for 10 min on ice. After sonication, cell debris was removed by centrifugation at 16,000 rpm at 4°C for 40 min. Suspensions were loaded on a 4 mL Ni-NTA agarose column equilibrated with 50 mM Tris-HCl (pH 7.5) and 0.3 M NaCl, and washed with 10 mM imidazole. Concentration and buffer exchanges in the serpin fractions were performed in an Amicon Ultra 10K cartridge (Millipore, Billerica, MA, USA). The purified proteins were stored at –80°C in 20 mM Tris-HCl (pH 7.5).

### Immunoaffinity chromatography and LC-MS/MS

Rabbit antibody covalently coupled Protein A-agarose was prepared as previously described [51]. Cell-free hemolymph samples (0.1 mL) were collected from mock-infected or infected larvae. PTU (10 mM) was added to the hemolymph and incubated at room temperature for 30 min, after which PMSF (to a final concentration of 1 mM) and protease inhibitor cocktail (Pierce, Waltham, MA, USA) (1:100) were added to inhibit protease activity. The samples were incubated with 50 μL of Protein A-agarose beads at 4°C for 5 h to remove non-specific absorption. After centrifugation at 12,000 rpm for 4°C, the supernatants were added to 50 μL of the rabbit antibody coupled Protein A-agarose beads and incubated at 4°C overnight. *Ha*serpin-protease complexes were washed and eluted as described in the Protein A Immunoprecipitation Kit (Sigma, St. Louis, MO, USA). The affinity-purified proteins were subjected to SDS-PAGE and stained with Coomassie Brilliant Blue. Subsequently, the gel lanes were excised into slices for in-gel digestion and LC-MS/MS analysis as described above.

### Survival analysis

At 12 h after dsRNA injection (described below), 24 larvae were starved for 12 h and then challenged with HearNPV (3.0×10^5^ OBs/μL, LC_95_) as described above. The larvae were maintained in individual containers and fed continuously. The survival curves were compared using Kaplan-Meier, the *p*-value threshold was calculated with a log-rank or Mantel Cox test; *p* < 0.05 was considered statistically significant. Graphpad 6.0 software was used in all of the statistical analyses.

### Yeast two-hybrid analysis

The experiments were performed as previously described [52], with primers listed in S6 Table. Serpins were cloned into pGBK7 and cSPs were introduced into pGADT7 plasmid. For the validation of serpin-5 and serpin-9 binding proteins, the two types of plasmids were co-transformed into AH109 cells using YeastMaker Yeast Transformation System 2 (Clontech, Mountain View, CA, USA), and the binding was validated in synthetic dropout-Leu-Trp-His medium supplemented with X-gal.

### Serpin-proteinase complex and PPO cleavage

Recombinant procSP4_Xa_ and procSP6_Xa_ proteins and high-purity native PPO were prepared as described below. procSP4 (50 ng) activated by bovine clotting factor Xa was mixed with purified serpin-5 at a molar ratio of 1:5; While in control sample, procSP4 or factor Xa was omitted from the mixture. After incubation at room temperature for 10 min, the mixtures were resolved by SDS-PAGE and subjected to immunoblot analysis as described above, using diluted antibodies against His-tag (1:8000) or serpin-5 (1:5000) as primary antibodies. In the immunoblot analysis using antibodies against His-tag, the amount of loading sample in the second lane was six times the expected amount to visualize the band. Amidase activity of the reaction mixtures was also measured using 200 μL, 50 μM acetyl-Ile-Glu-Ala-Arg-*p*-nitroanilide (IEAR) [53].The same processing method was used for procSP6 and serpin-9. Activated cSP6 was mixed with native PPO at a molar ratio of 1:1; in the control samples, procSP6 or factor Xa was omitted from the mixture. After incubation at room temperature for 10 min, the mixtures were resolved by SDS-PAGE and subjected to immunoblot analysis as described above, using diluted antibody against PPO1 as the primary antibody. Amidase activity and PO activity in the reaction mixtures were also assayed as described above.

### Purification of PPO from larval hemolymph

PPO was purified from the hemolymph of *H*. *armigera* larvae using the previously described method [54] with modifications. Unless otherwise stated, all of the operations were conducted in a cold room (3–5°C) and centrifugations were performed at 12,000×*g* for 20 min. Hemolymph (10 mL) from 200 day-3 5^th^ instar larvae was collected (approximately 50 μL/Larva) directly into ice-cold saturated ammonium sulfate (AS) solution to prevent the activation of PPO. The final concentration of AS was adjusted to 35% saturation. After centrifugation, the supernatant was collected and brought to 50% AS saturation. The protein precipitate was collected by centrifugation and dissolved in 2 mL buffer A (10 mM potassium phosphate buffer, pH 6.8, containing 500 mM NaCl, and 0.5 mM glutathione). The protein solution was exchanged against buffer A three times and concentrated to 600 μL by filtration over an Amicon Ultra 30K cartridge (Millipore). The 600 μL protein solution was loaded on a 5 mL column prepacked with Ceramic Hydroxyapatite (Bio-Rad) equilibrated with buffer B (10 mM potassium phosphate buffer, pH 6.8, containing 500 mM NaCl) and washed with 15 mL buffer B. Bound proteins were eluted at 1 mL/min with a linear gradient of 10–100 mM potassium phosphate in buffer B (30 mL). Pooled PPO fractions were adjusted to 1 mM MgCl_2_ and passed through a 0.5 mL Concanavalin A (Con A) Sepharose column (Sigma) equilibrated with 20 mM Tris-HCl (pH 7.4), 0.5 M NaCl, and 1 mM MgCl_2_. The flow-through fraction was adjusted with saturated AS to a final concentration of 1 M and applied to a 1 mL Phenyl Sepharose 6 Fast Flow (low sub) column (GE healthcare) equilibrated with 0.1 M potassium phosphate buffer (pH 7.1) and 1.0 M AS and washed with the same buffer (3 mL). Bound proteins were eluted with a descending gradient of 1–0 M AS and 0.1–0.01 M potassium phosphate (20 mL). The PPO fractions were combined and concentrated to 0.5 mL and loaded onto a 25 mL Superdex 200 column (AKTApurifier System, GE Healthcare, Little Chalfont, UK) equilibrated with 10 mM Tris-HCl (pH 7.5) and 0.2 M NaCl. Fractions containing PPO were combined and protein concentration was determined by the BCA method. PPO was stored at –80°C. After each chromatographic step, the PO activity in column fractions was assayed by activation of PPO with cetylpyridinium chloride using the 2 mM dopamine solution.

### Recombinant protein expression in the *Drosophila* S2 cell system

The entire procSP4 and procSP6 coding region, excluding the signal peptide, were amplified by RT-PCR from the fat body of 5^th^ instar *H*. *armigera* larvae. The primers are listed in S6 Table. The PCR products were gel purified, digested with *Eco*RI and *Xba*I and ligated into the same site in pMT-BiP/V5-HisA vector (Invitrogen). After sequence confirmation, the resulting plasmids were used as templates to produce mutants in which the four residues at the predicted activation site were replaced with IEGR, a cleavage site of bovine coagulation factor Xa, using overlap extension PCR [55]. The primers are listed in S6 Table. These constructs were named procSP4_Xa_ and procSP6_Xa_. After sequence verification, the plasmids were used to transfect *Drosophilia* S2 cells in combination with hygromycin selection vector pCoHygro (Invitrogen), and stable cell lines for producing recombinant proteins were obtained following the manufacturer’s instructions.

The cell cultures were harvested at 6 days after induction of expression with 500 μM CuSO_4_. Cells were removed by centrifugation at 1,000 *g* at 4°C for 5 min, and the cell debris was removed by centrifugation at 14,000 *g* at 4°C for 1 h. The cell-free medium (400 mL) was loaded onto a 5 mL Ni-NTA agarose column equilibrated with phosphate buffered saline (PBS; pH 7.4) and washed with 10 mM imidazole. Bound proteins were eluted with 100 mM imidazole, exchanged against 20 mM Tris-HCl (pH 8.0) and 0.1 M NaCl, and concentrated by filtration over an Amicon Ultra 10K cartridge. The purified proteins were stored at –80°C.

### The effects of melanization on baculovirus

A recombinant stock of HearNPV-*egfp* [50] (10 μL, 5×10^5^ TCID_50_/mL) was mixed with either 5 μL hemolymph (*H*. *armigera* cell-free hemolymph from healthy 5^th^ instar larvae), 20 μL of 10 mM dopa, 5 μL hemolymph and 20 μL of 10 mM dopa, 5 μL hemolymph and 20 μL of 10 mM dopa with 10 μL of 10 mM PTU, or 10 μL of 1.25 mM DHI in 80μL phosphate buffer (PB; 50 mM sodium phosphate, pH 6.5), then all of the mixtures were adjusted to a final volume of 100 μL with PB and incubated at room temperature for 0, 1, 3, or 6 h. The virus titers of these mixtures were determined by end-point dilution assay using HzAM1 cells. Each virus infection was performed in triplicate. Moreover, the reaction mixtures (3 μL) were added to HzAM1 cells in Grace’s insect medium supplemented with 10% FBS in 24-well plates (1.25×10^5^ cells/well); after incubation for 2 h, cells were washed with the serum-free medium three times and then medium containing 1% low melting point agarose was added to each well. Each treatment was performed in triplicate. After incubation at 27°C for 72 h, plaque formation in HzAM1 cells infected with variously treated viruses were examined by fluorescence microscopy.

### Synthesis and micro-injection of dsRNA

dsRNA of target genes was generated *in vitro* using T7 RiboMAX Express RNAi kits (Promega) according to the manufacturer’s instructions. The GFP gene was used to generate GFP dsRNA. 2 μg dsRNA was injected into the abdomen of late 2^nd^ instar larvae using a microinjector (World Precision Instrument, Sarasota, CA, USA). Primers used to generate dsRNA are listed in S6 Table. The transcript levels of target genes at 3 days after dsRNA injection were confirmed by qPCR.

### Quantitative PCR analysis of genomic DNA copies in hemolymph

The virus titers in hemolymph of insects where serpins were knocked down were determined by qPCR analyses of viral genomic DNA, using primers of *ha39*. HearNPV Bacmid DNA was isolated according to Bac-to-Bac manual (In vitrogen) and quantified by spectrophotometry at OD_260_. A standard curve was generated with ten-fold dilutions of Bacmid DNA. The cell-free hemolymph (1 μL) from serpin-5 or serpin-9 depleted larvae infected with HearNPV, were collected into 19 μL H_2_O, 80 μL virus disruption buffer (10 mM Tris-HCl pH7.5, 10 mM EDTA, 0.25% SDS) and 5 μL proteinase K (20 mg/ml). Virions were lysed at 50°C for 1 h, and viral DNA was ethanol precipitated and resuspended in 30 μL H_2_O. The viral DNA was used as the PCR template.

### Data availability

The mass spectrometry data (PXD006126) have been deposited in the PRIDE repository (http://www.ebi.ac.uk/pride). All sequence data that support the findings of this study are available in GenBank with the following accession numbers: serpin-5 (KY680238), serpin-9 (KY680239), cSP4 (KY680240), cSP6 (KY680241), cSP8 (KY680243), cSP29 (KY680244), PPO1 (KY744277), PPO2 (KY744278).

## Supporting information

S1 Fig Quantitative proteomics analysis. (**a**) SDS polyacrylamide gel electrophoretic analysis of the mock-infected and infected larval hemolymph (n = 6) on 4–20% gradient gel. Each sample was normalized to 25 μg. (**b**) PCA of protein levels across the libraries at two time points. The first three components, PC1, PC2 and PC3, define the x-, y- and z-axes of the three-dimensional space, respectively, so the distance between two points reflects the variance in protein levels between them. (**c**) Distribution of identified immunity-related proteins in all groups. (**d**) Venn diagram analysis of identified differential immunity-related proteins at two time points post ingestion (48 and 72 hpi).
(TIF)

S2 Fig The specificity of cSP antibodies. (a) The qPCR analysis. The primers used in qPCR overlapped with the dsRNA corresponding region. * *p* ≤ 0.05, ** *p* ≤ 0.01. (b) Immunoblot analysis of the cSP RNAi silencing using cSP antibodies.
(TIF)

S3 Fig The protein levels of melanization components in hemolymph after baculovrius infection. (a) The protein levels of melanization components proteins were quantified by immuneJ software and HSP27.2 was used as a loading control. The average of protein level in each control litter was defined as 1. The average of protein level in each litter of the infected hemolymph on the same immunoblot was normalized to that of time-matched control. Results derived from three independent experiments were subjected to standard t test. * *p* ≤ 0.05, ** *p* ≤ 0.01. (b) Immunoblot analysis of PPOs in hemolymph after baculovirus infection. To compare the amount of cleaved PPOs between infected and control samples when the protein levels of the PPO zymogens in infected samples were comparable to or higher than those in control samples, the amounts of loading samples of 48hI and 72hI were five and seven times the expected amount respectively in immunoblot assay for antibodies to PPO1, and were four and six times the expected amount respectively in immunoblot assay for antibodies to PPO2.
(TIF)

S4 Fig Phylogenetic analysis and RCL region sequences comparison of identified serpins and serpins of from *Manduca sexta* (Ms) and *Drosophila melanogaster* (Dm). All the selected serpins were aligned and phylogenetic analysis by MEGA 6. Scale bar, 0.1 substitution per site (left panel). Alignment of the RCL sequences containing the scissile bond and activation sites (right panel). The P1-P1’ scissile bond is indicated by arrow.
(TIF)

S5 Fig Phylogenetic analysis of clip-domain serine protease-related proteins (CLIPs). The catalytic domain amino acid sequences of 11 *H*. *armigera* (Ha), 3 *D*. *melanogaster* (Dm), 8 *B*. *mori* (Bm), 5 *M*. *sexta* (Ms), 2 *A*. *gambiae* (Ag), 1 *T*. *molitor* (Tm) and 2 *T*. *tridentatus* (Tt) CLIPs are compared and divided into three groups (B~D) based on sequence similarity. Scale bar, 0.1 substitutions per site.
(TIF)

S6 Fig Confirmation of the serpin RNAi silencing efficiency at the protein level and immunoblot analysis of selected proteins. (a) The qPCR analysis. The primers used in qPCR overlapped with the dsRNA corresponding region. * *p* ≤ 0.05, ** *p* ≤ 0.01. (b) Immunoblot detecting the amounts of VP39, PPO2 cSP4, cSP6, and cSP29. iGFP, GFP dsRNA-treated larvae; iGFP/NPV, GFP dsRNA-treated larvae infected with NPV; iserpin-5/NPV, serpin-5 dsRNA-treated larvae infected with NPV; iserpin-9/NPV, serpin-9 dsRNA-treated larvae infected with NPV. *H*. *armigera* HSP27.2 (heat shock protein 27.2 kDa) was used as the loading control.
(TIF)

S7 Fig Ethanol active PPO purified from hemolymph. (**a**) Immunoblot analysis of 5 ng purified hemolymph PPO using PPO1 and PPO2 antibodies. (**b**) 2 μL hemolymph from naïve 5^th^ instar larvae and 100 ng purified hemolymph PPO were subjected to native gel and stained by dopamine, which was dissolved in ethanol. (**c**) 50 ng cSP6_Xa_ was activated by 400 ng factor Xa, and then incubated with 100 ng PPO at room temperature for 10 min. the mixture was subject to amidase activity assay. Values are expressed as mean ± s.e.m of three independent experiments.
(TIF)

S1 Table Pearson pairwise correlation among biological replicates. (PDF)

S2 Table Identified proteins encoded by baculovirus in infected hemolymph. (PDF)

S3 Table A whole list of differential proteins. (PDF)

S4 Table Repertoire of baculovirus-regulated immune protein in hemolymph. (PDF)

S5 Table List of antibodies produced in this study. (PDF)

S6 Table Primers used for qPCR, protein expression, dsRNA synthesis and yeast two-hybrid assay. (PDF)
