# Supplementary figures and images for: Inhibition of melanization by serpin-5 and serpin-9 promotes baculovirus infection in cotton bollworm Helicoverpa armigera
Source: PLoS Pathog. 2017 Sep 27;13(9):e1006645. doi: 10.1371/journal.ppat.1006645 (PMC5633200; doi:10.1371/journal.ppat.1006645)

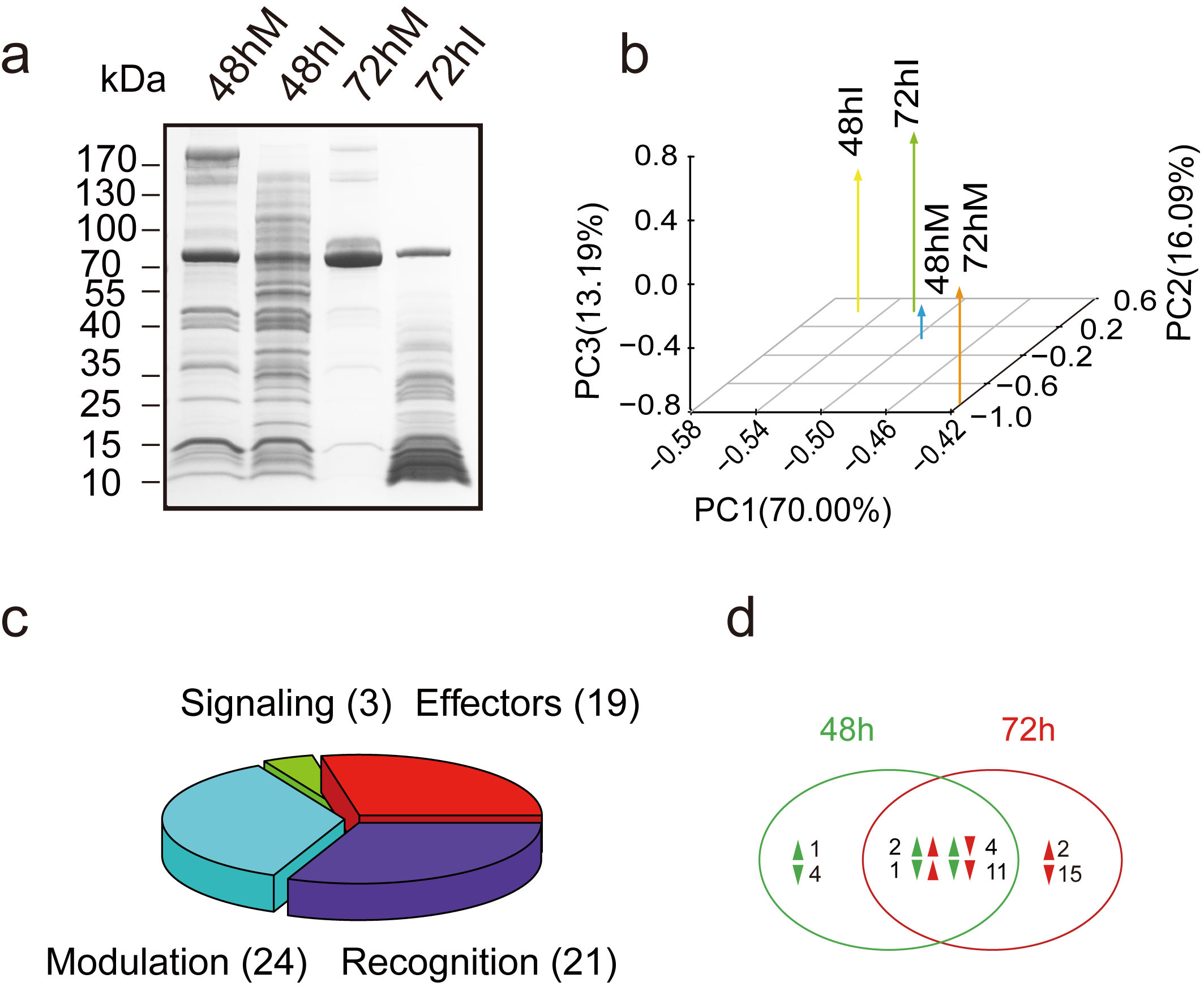

Supplement: S1 Fig — (a) SDS polyacrylamide gel electrophoretic analysis of the mock-infected and infected larval hemolymph (n = 6) on 4–20% gradient gel. Each sample was normalized to 25 μg. (b) PCA of protein levels across the libraries at two time points. The first three components, PC1, PC2 and PC3, define the x-, y- and z-axes of the three-dimensional space, respectively, so the distance between two points reflects the variance in protein levels between them. (c) Distribution of identified immunity-related proteins in all groups. (d) Venn diagram analysis of identified differential immunity-related proteins at two time points post ingestion (48 and 72 hpi). (TIF) [file ppat.1006645.s001.tif]

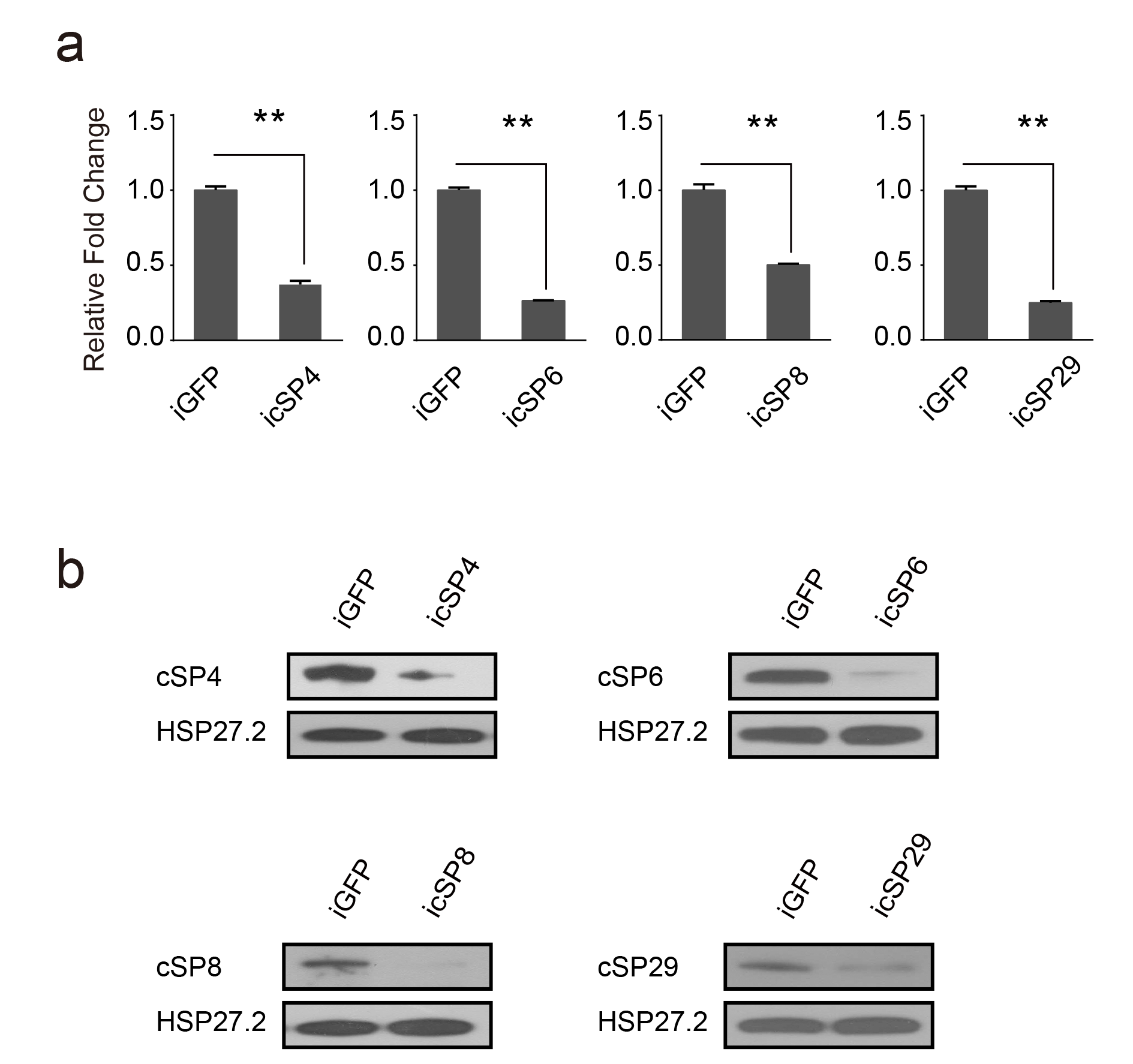

Supplement: S2 Fig — (a) The qPCR analysis. The primers used in qPCR overlapped with the dsRNA corresponding region. * p ≤ 0.05, ** p ≤ 0.01. (b) Immunoblot analysis of the cSP RNAi silencing using cSP antibodies. (TIF) [file ppat.1006645.s002.tif]

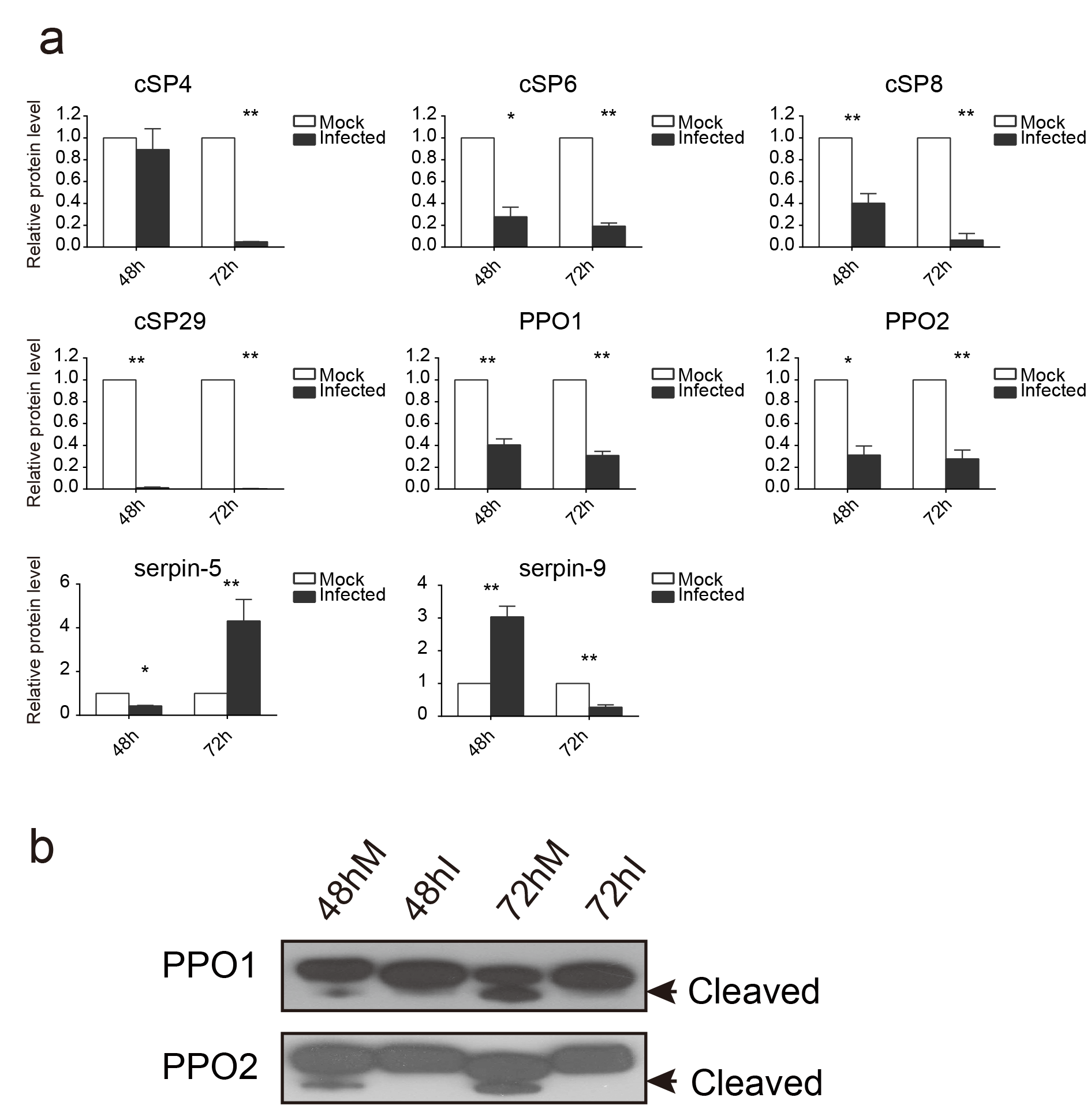

Supplement: S3 Fig — (a) The protein levels of melanization components proteins were quantified by immuneJ software and HSP27.2 was used as a loading control. The average of protein level in each control litter was defined as 1. The average of protein level in each litter of the infected hemolymph on the same immunoblot was normalized to that of time-matched control. Results derived from three independent experiments were subjected to standard t test. * p ≤ 0.05, ** p ≤ 0.01. (b) Immunoblot analysis of PPOs in hemolymph after baculovirus infection. To compare the amount of cleaved PPOs between infected and control samples when the protein levels of the PPO zymogens in infected samples were comparable to or higher than those in control samples, the amounts of loading samples of 48hI and 72hI were five and seven times the expected amount respectively in immunoblot assay for antibodies to PPO1, and were four and six times the expected amount respectively in immunoblot assay for antibodies to PPO2. (TIF) [file ppat.1006645.s003.tif]

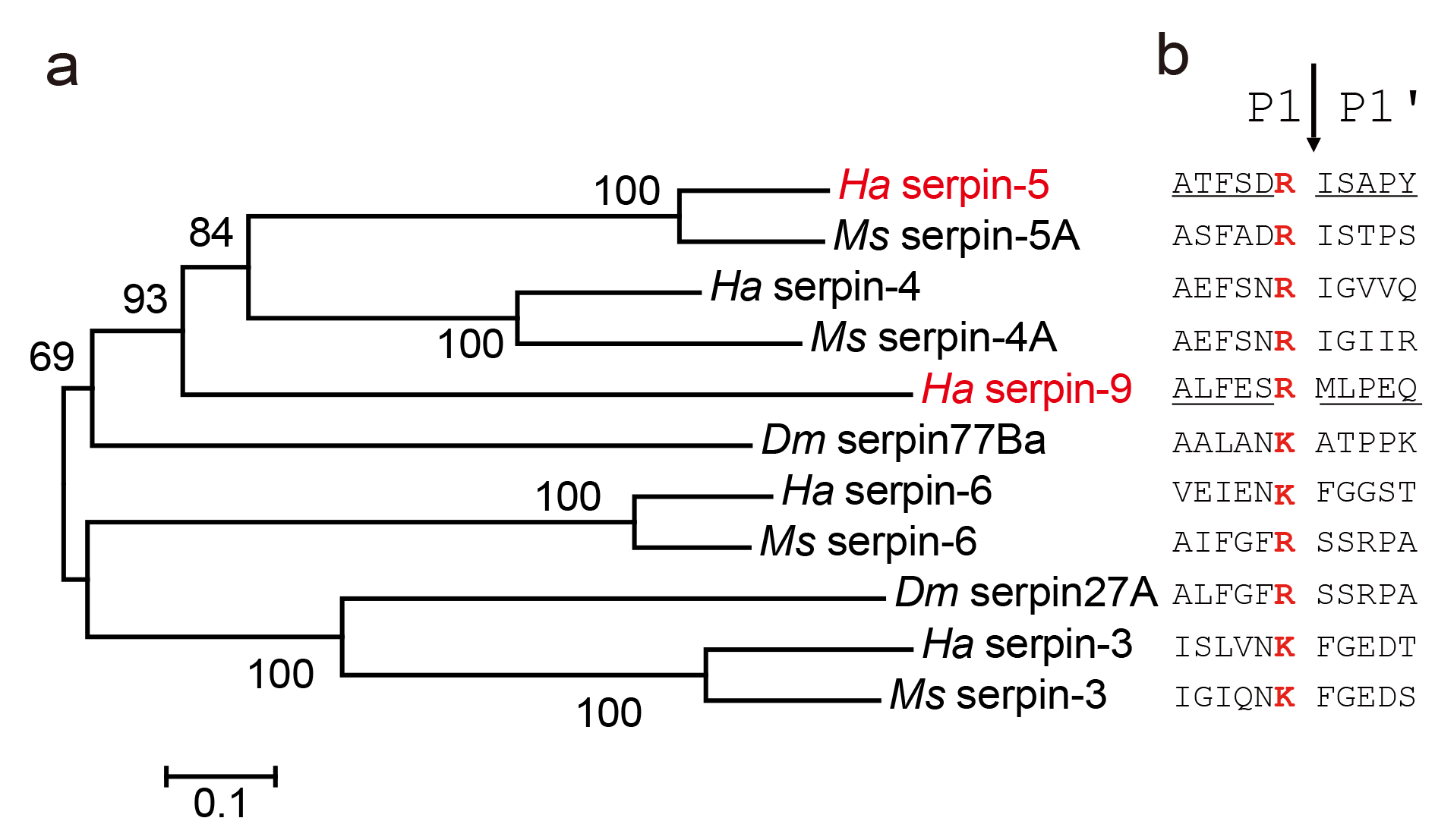

Supplement: S4 Fig — All the selected serpins were aligned and phylogenetic analysis by MEGA 6. Scale bar, 0.1 substitution per site (left panel). Alignment of the RCL sequences containing the scissile bond and activation sites (right panel). The P1-P1’ scissile bond is indicated by arrow. (TIF) [file ppat.1006645.s004.tif]

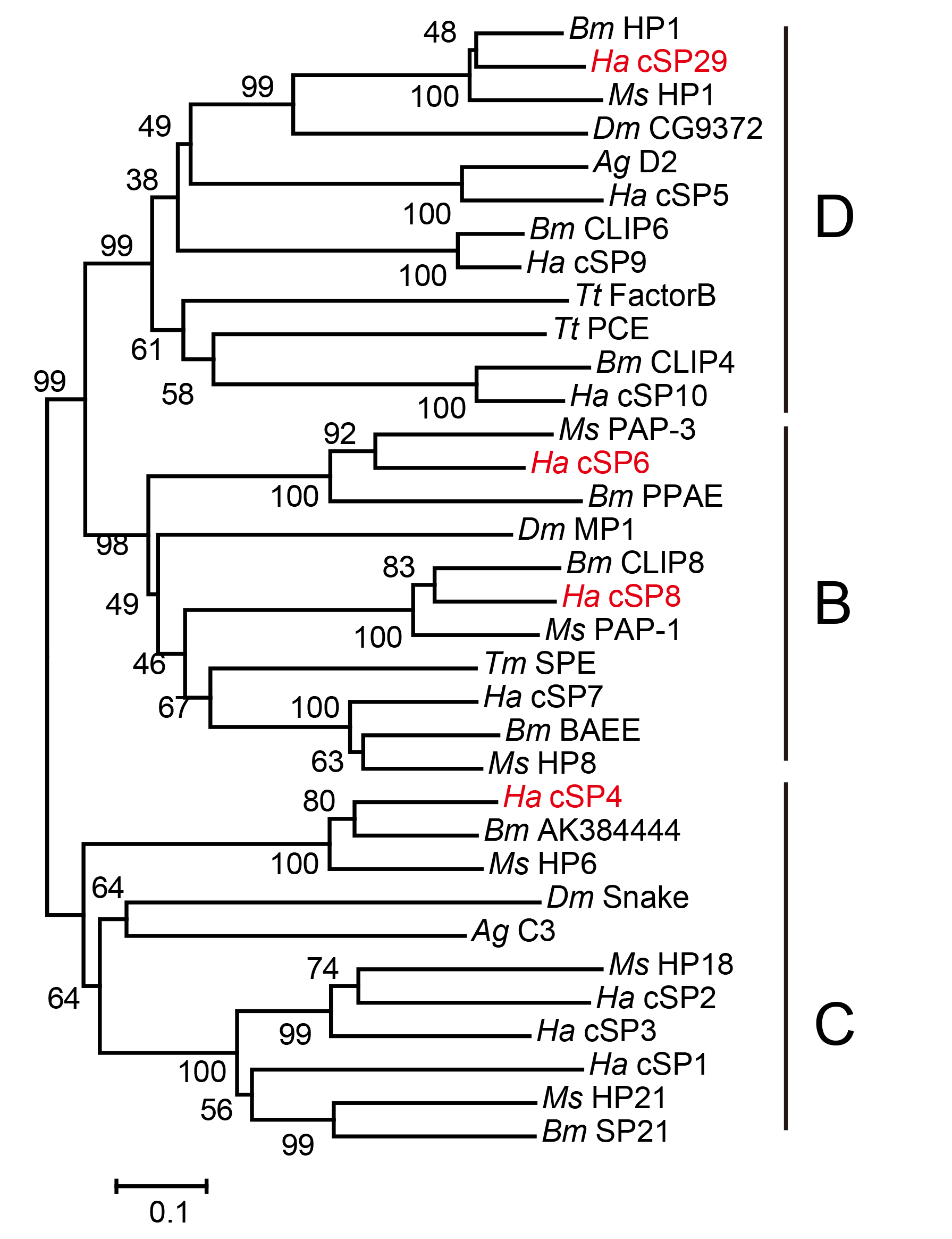

Supplement: S5 Fig — The catalytic domain amino acid sequences of 11 H. armigera (Ha), 3 D. melanogaster (Dm), 8 B. mori (Bm), 5 M. sexta (Ms), 2 A. gambiae (Ag), 1 T. molitor (Tm) and 2 T. tridentatus (Tt) CLIPs are compared and divided into three groups (B~D) based on sequence similarity. Scale bar, 0.1 substitutions per site. (TIF) [file ppat.1006645.s005.tif]

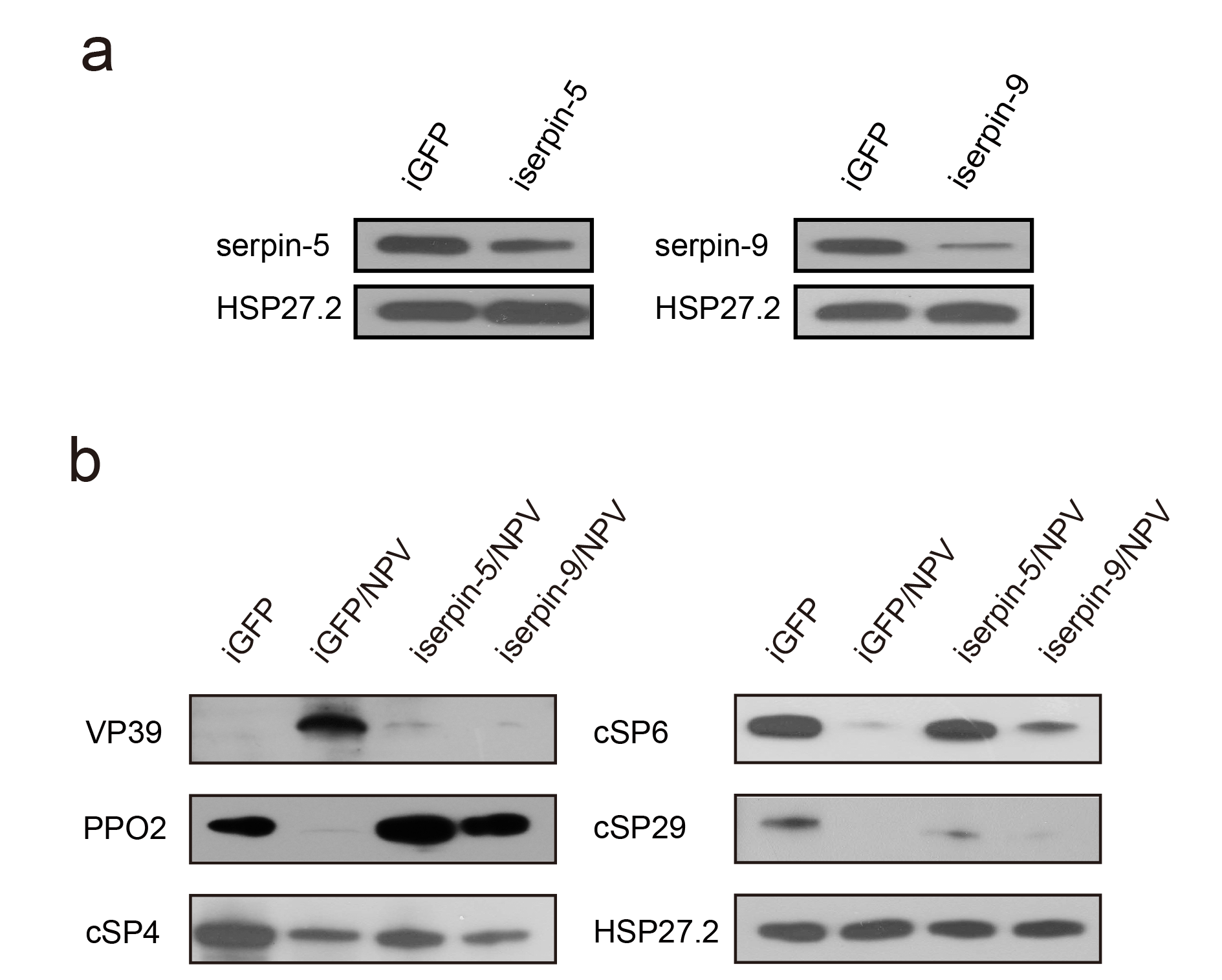

Supplement: S6 Fig — (a) The qPCR analysis. The primers used in qPCR overlapped with the dsRNA corresponding region. * p ≤ 0.05, ** p ≤ 0.01. (b) Immunoblot detecting the amounts of VP39, PPO2 cSP4, cSP6, and cSP29. iGFP, GFP dsRNA-treated larvae; iGFP/NPV, GFP dsRNA-treated larvae infected with NPV; iserpin-5/NPV, serpin-5 dsRNA-treated larvae infected with NPV; iserpin-9/NPV, serpin-9 dsRNA-treated larvae infected with NPV. H. armigera HSP27.2 (heat shock protein 27.2 kDa) was used as the loading control. (TIF) [file ppat.1006645.s006.tif]

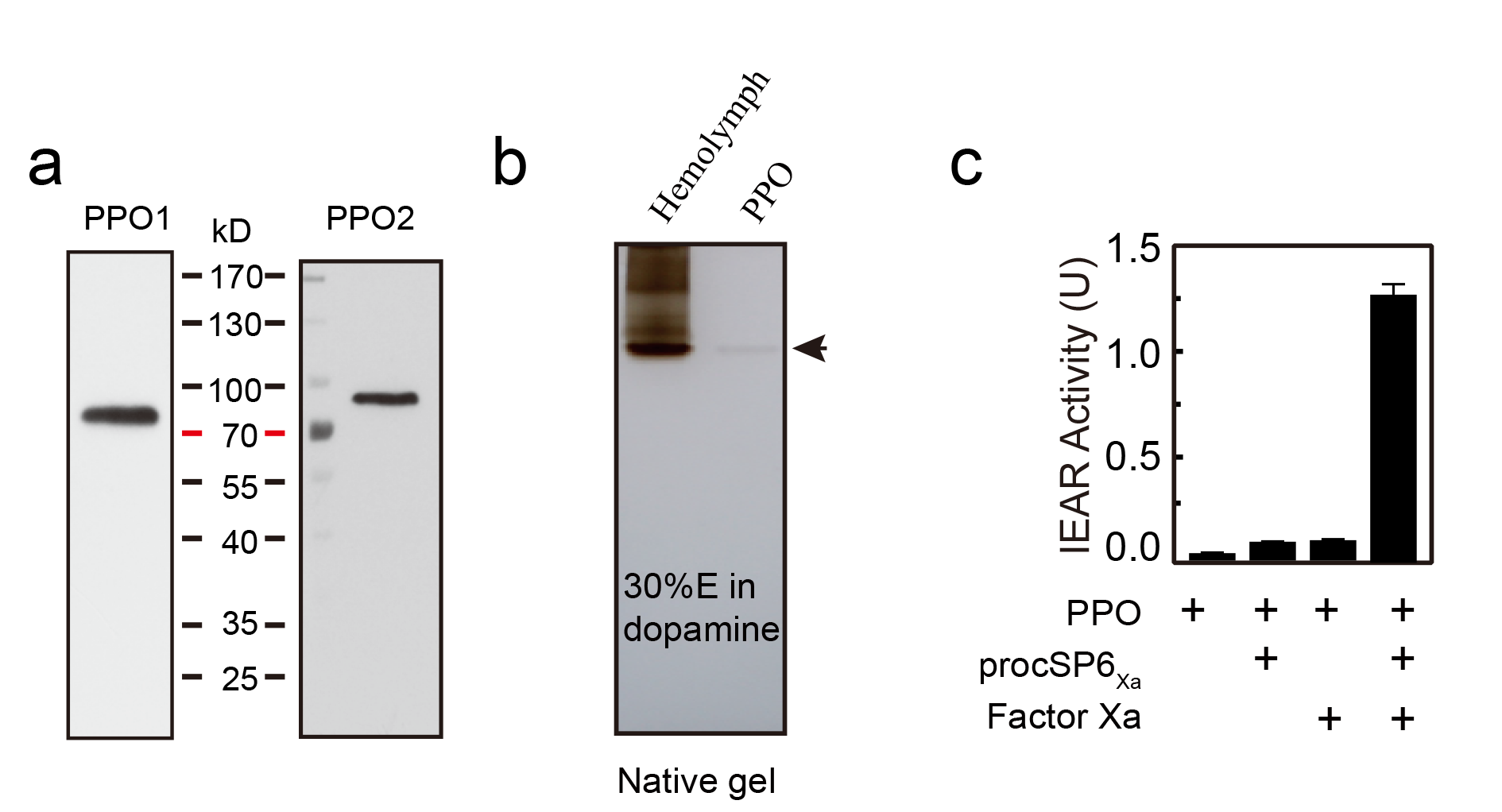

Supplement: S7 Fig — (a) Immunoblot analysis of 5 ng purified hemolymph PPO using PPO1 and PPO2 antibodies. (b) 2 μL hemolymph from naïve 5th instar larvae and 100 ng purified hemolymph PPO were subjected to native gel and stained by dopamine, which was dissolved in ethanol. (c) 50 ng cSP6Xa was activated by 400 ng factor Xa, and then incubated with 100 ng PPO at room temperature for 10 min. the mixture was subject to amidase activity assay. Values are expressed as mean ± s.e.m of three independent experiments. (TIF) [file ppat.1006645.s007.tif]
