# Supplementary material for: Inhibition of melanization by serpin-5 and serpin-9 promotes baculovirus infection in cotton bollworm Helicoverpa armigera
Source: PLoS Pathog. 2017 Sep 27;13(9):e1006645. doi: 10.1371/journal.ppat.1006645 (PMC5633200; doi:10.1371/journal.ppat.1006645)
Supplement: S1 Table — (PDF) [file ppat.1006645.s008.pdf]

Supplementary Data 1. Pearson pairwise correlation among biological replicates

	48hM1	48hM2	48hM3	48hI1	48hI2	48hI3
48hM1	1	0.94	0.94	0.83	0.78	0.72
48hM2		1	0.99	0.84	0.76	0.68
48hM3			1	0.83	0.76	0.69
48hI1				1	0.97	0.94
48hI2					1	0.99
48hI3						1
	72hM1	72hM2	72hM3	72hI1	72hI2	72hI3
72hM1	1	0.99	0.99	0.51	0.57	0.41
72hM2		1	1.00	0.53	0.58	0.42
72hM3			1	0.53	0.59	0.43
72hI1				1	0.99	0.98
72hI2					1	0.98
72hI3						1
