# Supplementary material for: Inhibition of melanization by serpin-5 and serpin-9 promotes baculovirus infection in cotton bollworm Helicoverpa armigera
Source: PLoS Pathog. 2017 Sep 27;13(9):e1006645. doi: 10.1371/journal.ppat.1006645 (PMC5633200; doi:10.1371/journal.ppat.1006645)
Supplement: S2 Table — (PDF) [file ppat.1006645.s009.pdf]

Supplementary Data 2. Identified proteins encoded by baculovirus in infected hemolymph

ORF	Protein	Cluster	Promoter	Area 48 hpi	Area 72 hpi	Relative abundance 48 hpi	Relative abundance 72 hpi	p-value	FC
ORF56	cathepsin	Auxiliary	L	6.49E+09	3.42E+10	5.703	38.967	< 0.0001	6.83
ORF21	P10	Structural	L	6.26E+07	1.32E+10	0.026	10.761	0.000542571	413.88
ORF126	EGT	Auxiliary	L	7.66E+09	4.84E+09	6.553	6.531	0.979538	1
ORF22	P26	Structural	L	5.04E+10	4.45E+09	43.367	6.028	< 0.0001	0.14
ORF57	HA57	Structural	E	5.42E+08	2.65E+09	0.411	3.614	< 0.0001	8.79
ORF41	chitinase	Auxiliary/Structu		3.78E+08	2.44E+09	0.308	3.289	< 0.0001	10.68
ORF120	Calyx/PEP	Structural	L	0.00E+00	2.97E+09	0	2.984	< 0.0001	Infinity
ORF68	HA68	Structural	L	9.14E+08	2.43E+09	0.808	2.937	< 0.0001	3.63
ORF10	HA10	Structural	L	5.00E+08	1.62E+09	0.409	2.129	< 0.0001	5.21
ORF38	LEF-8	Structural /Tran		0.00E+00	9.42E+08	0	1.32	0.0649441	Infinity
ORF82	ODV-E25	Structural		1.01E+08	8.71E+08	0.066	0.863	< 0.0001	13.08
ORF69	HA69	Structural	L	1.07E+08	2.91E+08	0.115	0.497	0.00140351	4.32
ORF53	FP25K	Structural	L	0.00E+00	3.99E+08	0	0.486	< 0.0001	Infinity
ORF78	VP39	Structural		4.88E+08	3.64E+08	0.4	0.473	0.29537	1.18
ORF106	ODV-E18	Auxiliary/Structu		1.28E+08	3.82E+08	0.127	0.472	< 0.0001	3.72
ORF133	HA133	Structural	L,E	4.42E+08	3.84E+08	0.371	0.469	0.13936	1.26
ORF91	HA91	Structural	E,L	1.20E+08	3.44E+08	0.107	0.451	< 0.0001	4.21
ORF1	POLH	Structural	L	1.55E+08	3.75E+08	0.122	0.401	< 0.0001	3.29
ORF26	HA26	Structural		1.38E+08	2.27E+08	0.105	0.283	< 0.0001	2.7
ORF113	FGF	Auxiliary	E	2.47E+09	2.30E+08	1.894	0.256	0.00181913	0.14
ORF100	HA100	Structural		4.97E+07	1.75E+08	0.026	0.237	< 0.0001	9.12
ORF118	P24	Structural	L	0.00E+00	2.26E+08	0	0.23	0.000107157	Infinity
ORF44	HA44	Structural	L	1.19E+08	1.76E+08	0.098	0.216	0.000187616	2.2
ORF31	39K/PP31			0.00E+00	1.58E+08	0	0.214	< 0.0001	Infinity

ORF9	P49	Structural	L	6.16E+07	1.41E+08	0.059	0.214	0.000127974	3.63
ORF24	LEF-6	Structural		0.00E+00	1.38E+08	0	0.206	< 0.0001	Infinity
ORF81	HA81		L	8.36E+07	1.41E+08	0.077	0.194	0.000948731	2.52
ORF27	HA27		E	9.11E+07	1.21E+08	0.07	0.177	0.00134674	2.53
ORF75	HA75		E	1.55E+08	1.56E+08	0.124	0.176	0.543726	1.42
ORF94	HA94	Structural		0.00E+00	1.37E+08	0	0.158	< 0.0001	Infinity
ORF130	PKIP-1	Structural		1.38E+08	1.35E+08	0.116	0.147	0.206599	1.27
ORF65	LEF-3	Structural /Repl		0.00E+00	1.19E+08	0	0.142	< 0.0001	Infinity
ORF28	ubiquitin	Structural	L	4.93E+07	5.88E+07	0.033	0.115	0.0284037	3.48
ORF36	HA36		E	5.00E+07	7.07E+07	0.043	0.098	0.047305	2.28
ORF73	GP41	Structural	L	5.82E+07	6.43E+07	0.054	0.082	0.00352955	1.52
ORF96	ODV-E66	Structural	L	0.00E+00	4.12E+07	0	0.055	< 0.0001	Infinity
ORF13	HA13			0.00E+00	2.64E+07	0	0.037	0.00224754	Infinity
ORF58	GP37	Structural	E	0.00E+00	5.27E+07	0	0.033	0.0104187	Infinity
ORF3	VP80			0.00E+00	2.98E+07	0	0.027	0.0166779	Infinity
ORF128	HA128	Structural		0.00E+00	4.55E+07	0	0.025	0.00878269	Infinity
ORF84	helicase	Structural /Repl		0.00E+00	5.08E+07	0	0.022	0.00818922	Infinity
ORF66	HA66	Structural	L	0.00E+00	2.42E+07	0	0.021	0.00630817	Infinity
ORF61	HE65		E	0.00E+00	1.83E+07	0	0.021	0.00870314	Infinity
ORF16-17	ME53	Structural /Repl	E	4.28E+07	0.00E+00	0.016	0	0.0215562	0
ORF121	HA121		E	3.49E+07	0.00E+00	0.015	0	0.0118542	0
Sum				7.20E+10	7.60E+10	61.623	86.058		

Identified proteins encoded by baculovirus in 72hM samples

ORF	Protein	Area	Relative abundance
ORF56	cathepsin	4.67E+07	0.247
ORF22	P26	1.23E+09	10.9778

ORF126	EGT	4.68E+08	3.441
