# Supplementary material for: Inhibition of melanization by serpin-5 and serpin-9 promotes baculovirus infection in cotton bollworm Helicoverpa armigera
Source: PLoS Pathog. 2017 Sep 27;13(9):e1006645. doi: 10.1371/journal.ppat.1006645 (PMC5633200; doi:10.1371/journal.ppat.1006645)
Supplement: S3 Table — (PDF) [file ppat.1006645.s010.pdf]

Supplementary Data 3. A whole list of differential proteins

Assembly No.	Description	48hM	48hI	72hM	72hI	48hI/48hM	48hI/48hM Ttes	72hI/72hM	72hI/72hM Tte
comp82973_c0_seq	arylphorin precursor	45.54445	47.22222	1926.667	94.66666	1.037	0.622	0.049	< 0.0001
comp82973_c0_seq	arylphorin precursor	30.96667	40.61111	1514.444	108.2	1.311	0.002	0.071	< 0.0001
comp82973_c0_seq	arylphorin precursor	14.55889	24.55556	802.5555	51.2	1.687	0.003	0.064	< 0.0001
comp83115_c0_seq	larval serum protein 2	0.182678	0.254689	455.3333	0.926489	1.394	0.282	0.002	< 0.0001
comp78480_c0_seq	juvenile hormone binding protein	57.64444	64.73334	392.8889	35.05556	1.123	0.586	0.089	< 0.0001
comp83924_c1_seq	methionine-rich storage protein	0.050461	0.036067	319.5555	0.240445	0.715	0.519	0.001	< 0.0001
comp91639_c0_seq	apolipophorin precursor protein	85.94444	88.23333	318.2222	113.3	1.027	0.782	0.356	< 0.0001
comp91683_c0_seq	imaginal disc growth factor	76.23334	103.9444	262	25.16667	1.364	0.174	0.096	< 0.0001
comp90179_c0_seq	vitellogenin	8.728889	82.1	248.9111	163.8667	9.406	< 0.0001	0.658	0.075
comp90255_c0_seq	vitellogenin	7.643333	45.55556	199.9111	80.21111	5.960	< 0.0001	0.401	0.003
comp87291_c1_seq	adhesion-like transmembrane prote	56.98889	74.71111	192.1111	13.49222	1.311	0.062	0.070	< 0.0001
comp88772_c0_seq	moderately methionine rich storage	0	0	174.5556	0.066469			0.000	< 0.0001
comp85106_c1_seq	af361483_1chymotrypsin inhibitor c	56.27778	77.68889	146.2111	27.83333	1.380	0.048	0.190	< 0.0001
comp85106_c1_seq	serpin 1	56.27778	77.68889	146.2111	27.83333	1.380	0.048	0.190	< 0.0001
comp73586_c0_seq	moderately methionine rich storage	0	0	130.2111	0.094926			0.001	< 0.0001
comp82850_c0_seq	vitellogenin	3.061111	7.777778	122.8667	7.762222	2.541	< 0.0001	0.063	< 0.0001
comp90171_c0_seq	vitellogenin	10.69222	158.4444	117.5667	260.8889	14.819	< 0.0001	2.219	< 0.0001
comp73970_c0_seq	moderately methionine rich storage	0	0	106.5333	0			0.000	< 0.0001
comp82184_c0_seq	cationic peptide cp8 precursor	22.09556	32.25555	76.96667	21.90444	1.460	0.119	0.285	0.008
comp88138_c0_seq	serine protease	2.387111	4.571111	50.6	0.819667	1.915	0.058	0.016	< 0.0001
comp84081_c0_seq	27 kda hemolymph protein	25.25767	51.55555	50.57778	44.93333	2.041	0.003	0.888	0.468
comp71771_c0_seq	vitellogenin	1.135556	2.625556	47.57778	2.214444	2.312	0.071	0.047	0.004
comp80209_c0_seq	scolexin b	10.10389	4.106667	32.4	0.824778	0.406	0.008	0.025	< 0.0001
comp81850_c0_seq	rna-binding protein 39	0	0	30.76667	0.399			0.013	0.011
comp87712_c1_seq	vitellogenin	0.541856	1.400333	26.07556	1.544889	2.584	0.004	0.059	0.004
comp86980_c1_seq	isoform a	10.01667	11.11444	22.95555	2.8	1.110	0.652	0.122	< 0.0001
comp80724_c0_seq	juvenile hormone binding protein	5.103611	8.992222	20.24444	5.167778	1.762	0.010	0.255	< 0.0001
comp86980_c0_seq	isoform a	7.6	8.741111	19.47667	2.577778	1.150	0.358	0.132	0.001
comp79980_c0_seq	odorant-binding protein	11.63	12.92111	19.07667	2.862222	1.111	0.696	0.150	< 0.0001
comp88521_c1_seq	transferrin	4.804444	12.29333	18.83333	7.611111	2.559	0.024	0.404	< 0.0001
comp85253_c0_seq	inter-alpha-trypsin inhibitor heavy ch	5.267778	10.96	18.44444	2.612222	2.081	0.002	0.142	< 0.0001

comp80301_c0_seq	vitellogenin	3.746667	42.24445	18.28889	56.47778	11.275	< 0.0001	3.088	< 0.0001
comp82224_c0_seq	mdl1	10.85222	13.31778	17.78556	6.433333	1.227	0.221	0.362	0.001
comp80997_c0_seq	PREDICTED: uncharacterized prote	6.654445	8.028889	16.43333	4.514444	1.207	0.295	0.275	< 0.0001
comp81655_c0_seq	odorant-binding protein	15.43	15.39778	15.83333	5.004445	0.998	0.990	0.316	0.000
comp90100_c0_seq	low quality protein: hemicentin-1	6.902222	5.96	15.04667	2.103333	0.863	0.492	0.140	< 0.0001
comp89974_c0_seq	low quality protein: neuroglian-like	8.833333	8.991111	14.84	2.312222	1.018	0.929	0.156	< 0.0001
comp84532_c0_seq	hypothetical protein KGM_13324	4.750778	5.426667	12.10444	1.423333	1.142	0.424	0.118	< 0.0001
comp85218_c0_seq	imp dehydrogenase gmp reductase	1.152	5.542222	12.03333	1.479889	4.811	0.000	0.123	< 0.0001
comp77695_c0_seq	MBF2	3.661889	8.624445	11.00333	1.621667	2.355	0.001	0.147	< 0.0001
comp86474_c0_seq	beta- -glucan recognition protein 2	2.901889	2.376667	10.86111	1.055556	0.819	0.395	0.097	< 0.0001
comp79150_c0_seq	REPAT31	6.683333	7.414444	10.77889	2.485556	1.109	0.609	0.231	0.000
comp71102_c0_seq	REPAT32	4.649	5.646667	10.74555	2.173333	1.215	0.205	0.202	0.000
comp88174_c0_seq	seminal fluid protein hacp058	0	0	9.988889	0			0.000	0.004
comp86024_c0_seq	serine protease inhibitor 5	3.555556	2.176667	9.974444	0.684222	0.612	0.170	0.069	< 0.0001
comp83709_c0_seq	c-type lectin	1.483167	0.545333	9.853333	1.604445	0.368	0.004	0.163	< 0.0001
comp88113_c0_seq	growth blocking peptide binding pro	3.778889	0.897444	9.762222	2.15	0.237	< 0.0001	0.220	0.001
comp88113_c0_seq	growth blocking peptide binding pro	3.723333	0.897444	9.762222	2.143889	0.241	< 0.0001	0.220	0.001
comp89077_c0_seq	prophenoloxidase subunit 2	10.43556	0.739111	9.503333	4.037778	0.071	< 0.0001	0.425	0.001
comp1652969_c0_s	seminal fluid protein hacp037	0.890445	0.695222	9.398889	0	0.781	0.667	0.000	0.006
comp88680_c0_seq	prophenoloxidase activating factor	1.3251	1.857778	9.383333	2.292222	1.402	0.195	0.244	< 0.0001
comp82961_c0_seq	serpin 1	3.323333	3.196667	8.83	2.905555	0.962	0.742	0.329	0.002
comp86039_c0_seq	vitellogenin	1.102667	2.828889	8.777778	2.212222	2.565	< 0.0001	0.252	< 0.0001
comp87656_c1_seq	protein cueball-like	1.920222	2.928889	8.701111	2.687778	1.525	0.178	0.309	0.000
comp88491_c0_seq	prophenoloxidase subunit 1	7	0.737111	7.587778	3.004444	0.105	< 0.0001	0.396	0.002
comp80862_c0_seq	imp dehydrogenase gmp reductase	2.739189	0.308444	7.552222	0.081711	0.113	< 0.0001	0.011	< 0.0001
comp80466_c0_seq	cysteine proteinase inhibitor precurs	4.883333	4.95	7.538889	2.844445	1.014	0.957	0.377	0.001
comp82330_c0_seq	carboxylesterase	2.751111	2.571111	7.477778	0.810778	0.935	0.739	0.108	0.000
comp80619_c0_seq	ly-6 neurotoxin superfamily membe	3.718667	0.962	7.231111	0.763889	0.259	0.001	0.106	0.000
comp89153_c0_seq	tpa: transposase domain-containing	0.704144	2.367	6.991111	3.264444	3.362	0.007	0.467	0.109
comp86871_c1_seq	serpin b3-like	3.385556	2.997778	6.971111	3.108889	0.885	0.153	0.446	0.010
comp88492_c0_seq	uncharacterized protein LOC77848	0.439889	1.591	6.725555	0.309556	3.617	0.009	0.046	< 0.0001
comp90400_c0_seq	amine oxidase	0	2.06	6.702222	0	Infinity	< 0.0001	0.000	0.006
comp84179_c0_seq	alkaline phosphatase	0.213444	0.146778	6.574	0	0.688	0.412	0.000	0.021
comp90133_c0_seq	serine protease inhibitor	2.595556	1.732222	6.451111	1.107333	0.667	0.014	0.172	< 0.0001

comp88651_c0_seq	glucose-1-phosphatase inositol pho	3.182555	4.051111	6.25	1.402778	1.273	0.254	0.224	0.003
comp88113_c0_seq	paralytic peptide binding protein 2	2.23	0.541222	6.071111	0.513444	0.243	< 0.0001	0.085	0.002
comp85451_c0_seq	serine protease	3.284556	0.854889	6.036666	0.2679	0.260	< 0.0001	0.044	0.001
comp81880_c1_seq	odorant-binding protein	4.517	4.168889	6.033333	0.324222	0.923	0.836	0.054	0.001
comp91253_c0_seq	myosin isoform c	0.5438	0.355778	5.77	0.797222	0.654	0.232	0.138	< 0.0001
comp81880_c1_seq	odorant-binding protein	3.235556	2.283333	5.453333	0.272889	0.706	0.048	0.050	0.001
comp90792_c1_seq	carboxypeptidase d-like	0	0	5.447778	0			0.000	0.007
comp89413_c0_seq	flotillin- isoform a	2.297333	2	5.328889	0.170144	0.871	0.486	0.032	0.002
comp87363_c0_seq	tetraspanin e118	2.35	0.857667	5.305555	0.89	0.365	0.002	0.168	0.006
comp81880_c1_seq	odorant-binding protein	4.689889	2.943111	5.248889	0	0.628	0.339	0.000	0.002
comp85242_c0_seq	c-type lectin	1.510733	0.408889	5.133333	0.339	0.271	0.001	0.066	< 0.0001
comp86608_c0_seq	abc transporter	2.867	0.664333	4.882222	0.551444	0.232	0.000	0.113	0.001
comp77265_c0_seq	mdl2	4.299667	5.94	4.841111	0.787444	1.382	0.118	0.163	0.009
comp86525_c0_seq	ly-6 neurotoxin superfamily membe	1.642333	0.604	4.476666	0.406	0.368	0.006	0.091	0.011
comp89216_c0_seq	juvenile hormone esterase	0.3643	0	4.418889	0	0.000	< 0.0001	0.000	< 0.0001
comp76631_c0_seq	heat shock protein	0.910922	1.799889	4.402222	0.981889	1.976	0.041	0.223	< 0.0001
comp84530_c0_seq	carboxylesterase 3	1.694444	0.676778	4.17	0.332556	0.399	< 0.0001	0.080	< 0.0001
comp85107_c0_seq	adhesion related transmembrane	1.925778	0.421667	4.142222	0.046767	0.219	0.002	0.011	< 0.0001
comp80381_c0_seq	proline-rich extensin-like protein epr	0.537644	4.850222	4	0.068344	9.021	0.077	0.017	0.022
comp88300_c0_seq	fat- isoform a	1.804444	1.128889	3.932222	0.055111	0.626	0.059	0.014	0.015
comp86618_c0_seq	nimrod b2	2.607889	3.094666	3.898889	0.542111	1.187	0.697	0.139	0.038
comp87712_c0_seq	vitellogenin	0.551222	1.585111	3.84	1.92	2.876	0.017	0.500	0.006
comp84808_c0_seq	copper-zinc superoxide dismutase	0.689889	1.004778	3.813333	2.966667	1.456	0.008	0.778	0.142
comp87025_c0_seq	ommochrome binding protein 2	0.952222	2.063333	3.735556	0.273333	2.167	0.048	0.073	< 0.0001
comp82509_c0_seq	follicular epithelium yolk protein sub	2.784967	2.312222	3.641111	0.617111	0.830	0.424	0.169	0.001
comp84605_c0_seq	unknown	2.167778	0.906111	3.618889	0.572556	0.418	0.005	0.158	0.000
comp83380_c4_seq	elongation factor 1 alpha	0.993333	1.145778	3.487778	1.717778	1.153	0.385	0.493	0.006
comp87656_c1_seq	low quality protein: protein cueball-li	1.587	1.372222	3.447778	0.844	0.865	0.487	0.245	0.007
comp82925_c0_seq	vitellogenin	1.034556	0.977	3.444656	0.9936	0.944	0.744	0.288	0.011
comp85861_c0_seq	gamma-interferon-inducible lysosor	2.321911	1.309556	3.261111	0.310878	0.564	0.048	0.095	0.068
comp89699_c0_seq	counting factor associated protein d	0.239644	0	3.258889	0	0.000	0.011	0.000	0.010
comp91604_c0_seq	isoform b	2.226667	0.174111	3.242556	0.389556	0.078	0.000	0.120	0.077
comp84067_c0_seq	odorant-binding protein	1.677667	3.637778	3.074556	7.062222	2.168	0.006	2.297	0.025
comp89651_c0_seq	fumarate mitochondrial	1.433222	0.231333	3.005556	0.250533	0.161	< 0.0001	0.083	0.000

comp88277_c0_seq	kinesin heavy chain	0	0.063456	2.893556	0.011052	Infinity	0.028	0.004	0.038
comp79036_c0_seq	apolipoprotein d-like	1.502889	4.802222	2.868889	0.992778	3.195	< 0.0001	0.346	0.003
comp89939_c0_seq	beta-galactosidase-like	1.808889	2.657778	2.847778	1.449667	1.469	0.002	0.509	0.008
comp81960_c0_seq	pyrimidine-specific ribonucleoside h	0.787994	0.902889	2.808889	0.238556	1.146	0.563	0.085	< 0.0001
comp87697_c0_seq	ferritin light chain	1.267533	6.384444	2.801667	10.71222	5.037	< 0.0001	3.824	< 0.0001
comp89030_c0_seq	serine proteinase stubble	0.259342	0.283444	2.696889	0.181933	1.093	0.761	0.067	0.001
comp85418_c0_seq	ester hydrolase c11orf54 homolog	0.626844	1.390556	2.665222	0.908889	2.218	0.045	0.341	0.002
comp83539_c0_seq	ejaculatory bulb-specific protein 3-li	0.7829	1.388444	2.627778	0.488511	1.773	0.037	0.186	< 0.0001
comp81495_c0_seq	actin	2.69	0.870222	2.612222	2.017778	0.324	0.014	0.772	0.177
comp61969_c1_seq	sparc	1.903333	0.039856	2.595111	0	0.021	< 0.0001	0.000	0.122
comp82636_c0_seq	plexin isoform a	0.948222	0.146467	2.537778	0.131111	0.154	< 0.0001	0.052	< 0.0001
comp61899_c0_seq	c-type lectin	0.160711	0.051756	2.532222	0	0.322	0.005	0.000	< 0.0001
comp76955_c0_seq	nadh:ubiquinone oxidoreductase co	1.263444	1.392444	2.522222	1.137889	1.102	0.703	0.451	0.005
comp87602_c0_seq	poly -specific endoribonuclease hor	1.437444	0.613444	2.513333	0.014017	0.427	0.002	0.006	< 0.0001
comp88228_c0_seq	arrestin domain containing 4	1.698378	0.436444	2.425555	0.308778	0.257	0.001	0.127	0.000
comp83305_c0_seq	serine protease inhibitor 010	1.442656	1.309889	2.42	0.172956	0.908	0.651	0.071	0.000
comp89275_c0_seq	integrator complex subunit 5	0.460445	0.560333	2.382222	0.622556	1.217	0.627	0.261	0.038
comp81880_c0_seq	odorant-binding protein	2.028478	0.331667	2.379	0	0.164	< 0.0001	0.000	0.158
comp90452_c0_seq	dystroglycan-like isoform x4	0.527778	0.054933	2.31	0.039756	0.104	< 0.0001	0.017	< 0.0001
comp80284_c0_seq	lysozyme	2.868	3.954444	2.290111	0.569778	1.379	0.518	0.249	0.024
comp87785_c0_seq	apolipoprotein d	0.410856	1.444111	2.218445	0.742556	3.515	0.000	0.335	0.001
comp84866_c1_seq	dimethylaniline monooxygenase	0.885556	0.655889	2.155555	0.211622	0.741	0.286	0.098	< 0.0001
comp85169_c0_seq	lachesin	1.242689	1.32	2.143333	0.668	1.062	0.837	0.312	0.000
comp88820_c1_seq	innexin 3	0.547989	0.110178	2.127778	0.0754	0.201	0.001	0.035	< 0.0001
comp74915_c0_seq	cathepsin d	0.245889	0.716222	2.056	0.1203	2.913	< 0.0001	0.059	0.000
comp88497_c0_seq	beaten path iia	0.992489	0.170689	2.006667	0.086033	0.172	0.001	0.043	< 0.0001
comp91206_c0_seq	integrin alpha-ps2-like	0.367444	0.301333	1.967444	0.097667	0.820	0.388	0.050	0.000
comp87279_c0_seq	dipeptidase 1-like	0.931111	0.902333	1.948889	0.190111	0.969	0.860	0.098	< 0.0001
comp89209_c1_seq	PREDICTED: uncharacterized prote	1.313556	0.658778	1.901111	0.884556	0.502	< 0.0001	0.465	0.001
comp79205_c0_seq	unknown secreted protein	1.085922	0.744622	1.900111	0.079544	0.686	0.206	0.042	< 0.0001
comp91641_c0_seq	apolipophorins-like isoform x2	0.677556	0.932778	1.873333	0.335667	1.377	0.007	0.179	< 0.0001
comp78334_c1_seq	c-type lectin	0.361089	0.160444	1.862222	0	0.444	0.020	0.000	< 0.0001
comp82165_c0_seq	niemann-pick type c-2a	0.031876	0.150444	1.776889	0	4.720	< 0.0001	0.000	< 0.0001
comp87223_c1_seq	pickpocket protein 28-like	1.015889	0.269778	1.737222	0.175889	0.266	< 0.0001	0.101	< 0.0001

comp91165_c0_seq	integrin beta-ps-like	0.488667	0.699667	1.714667	0.423111	1.432	0.193	0.247	< 0.0001
comp83793_c0_seq	atp-dependent -nad h-hydrate dehy	0.148846	0.300111	1.675678	0.0457	2.016	0.001	0.027	0.183
comp86273_c0_seq	prophenol oxidase activating enzym	0.57	0.397	1.666667	0.113289	0.696	0.005	0.068	< 0.0001
comp86890_c0_seq	yellow-fa precursor	1.086333	0.980889	1.663889	0.401778	0.903	0.541	0.241	0.002
comp87258_c0_seq	venom serine carboxypeptidase-like	1.148667	2.072222	1.641111	0.841556	1.804	< 0.0001	0.513	0.008
comp81572_c0_seq	innexin 2	0.412956	0.0956	1.616222	0	0.232	0.002	0.000	0.001
comp78735_c0_seq	bombyrin precursor	0.845	3.908889	1.610333	1.518889	4.626	< 0.0001	0.943	0.821
comp88454_c0_seq	paralytic peptide binding protein 2	0.469	0.082633	1.577333	0.081778	0.176	< 0.0001	0.052	0.001
comp87139_c0_seq	nadph:quinone reductase	0.546222	0.571111	1.567	0.259778	1.046	0.865	0.166	0.000
comp86896_c0_seq	protein cepu-1	0.849178	0.538889	1.539	0.251456	0.635	0.094	0.163	< 0.0001
comp83540_c0_seq	galectin 8	0.413967	0.128811	1.493556	0.1862	0.311	0.002	0.125	< 0.0001
comp79804_c0_seq	secreted peptide 30	0.380011	0.337333	1.488444	0	0.888	0.689	0.000	< 0.0001
comp83865_c0_seq	ommochrome binding protein 1	0.828	0.286222	1.446667	0	0.346	0.007	0.000	< 0.0001
comp88410_c0_seq	mitochondrial ribonuclease p protei	0	0	1.407778	0			0.000	0.048
comp89403_c0_seq	dna mismatch repair protein msh2	0.558556	1.412222	1.392889	0	2.528	0.029	0.000	< 0.0001
comp91491_c0_seq	laminin subunit alpha-like	1.401111	0.558889	1.365556	1.109444	0.399	0.000	0.812	0.168
comp91070_c0_seq	contactin	0.311111	0.117578	1.341222	0.0291	0.378	< 0.0001	0.022	< 0.0001
comp85916_c0_seq	transferase hexapeptide repeat pro	0.672667	0.908778	1.335111	0.206089	1.351	0.039	0.154	0.001
comp83833_c0_seq	small nuclear ribonucleoprotein	0	0.401667	1.325444	0	Infinity	0.005	0.000	0.000
comp83223_c0_seq	hypothetical protein KGM_12072	0	0	1.320556	0			0.000	0.000
comp84339_c0_seq	ommochrome binding protein 2	0.544089	0.0622	1.318667	0	0.114	0.000	0.000	< 0.0001
comp79487_c0_seq	c-type lectin 10	0.128122	0.044467	1.312222	0	0.347	0.008	0.000	< 0.0001
comp84521_c0_seq	hemolymph proteinase 8	0.253778	0.754444	1.279667	0.0735	2.973	< 0.0001	0.057	< 0.0001
comp91584_c0_seq	neurexin isoform b	0.300445	0.094144	1.277556	0.036678	0.313	< 0.0001	0.029	< 0.0001
comp87702_c0_seq	glucose-1-phosphatase inositol pho	1.034889	0.784111	1.259111	0.157211	0.758	0.107	0.125	0.000
comp80880_c0_seq	adenosine deaminase related grow	0.527056	1.637556	1.207	2.396667	3.107	0.007	1.986	0.001
comp82120_c0_seq	af250284_109 amv109	0.846267	0.625111	1.205889	0.256222	0.739	0.273	0.212	< 0.0001
comp71103_c0_seq	basement membrane-specific hepa	1.916667	0.894889	1.18	1.44	0.467	0.001	1.220	0.201
comp80377_c0_seq	c-type lectin	0.605268	0.699445	1.170667	0.234778	1.156	0.542	0.201	< 0.0001
comp79874_c0_seq	beta-hexosaminidase subunit beta-l	0	0	1.149889	0.067889			0.059	0.000
comp81117_c0_seq	pancreatic lipase-related protein 2-l	0.051556	0.07767	1.141678	0	1.507	0.326	0.000	< 0.0001
comp90525_c0_seq	leucine-rich repeat-containing prote	1.049444	0.0858	1.135	0.137667	0.082	< 0.0001	0.121	0.000
comp89947_c0_seq	angiotensin converting enzyme	1.200778	1.675556	1.104333	1.426667	1.395	< 0.0001	1.292	0.027
comp87695_c1_seq	hemolymph proteinase 19	0.368311	0.334889	1.094111	0.042311	0.909	0.839	0.039	< 0.0001

comp81495_c0_seq	actin beta subunit	0.690778	0.335	1.081111	1.14	0.485	0.018	1.054	0.849
comp88973_c0_seq	alkaline tissue-nonspecific isozyme-	0.435756	0.101878	1.08	0.050411	0.234	< 0.0001	0.047	< 0.0001
comp84512_c1_seq	plasma alpha-l-fucosidase	0.151708	0.080189	1.036778	0	0.529	0.071	0.000	0.000
comp87528_c1_seq	laminin subunit beta-1	1.213333	0.415333	1.029222	0.674778	0.342	< 0.0001	0.656	0.024
comp86229_c0_seq	carboxyl choline esterase	0.171117	1.366	1.021556	0	7.983	< 0.0001	0.000	< 0.0001
comp82713_c1_seq	odorant-binding protein 16	0.239944	0.295778	1.020444	0.333778	1.233	0.363	0.327	0.000
comp83370_c0_seq	hypothetical protein KGM_13675	0.303856	0.521778	1.012222	0.135911	1.717	0.013	0.134	0.002
comp85322_c0_seq	cg13532 cg13532-pa	0.624544	0.117267	0.997889	0.070233	0.188	0.001	0.070	0.007
comp83199_c0_seq	cathepsin b	0.088294	0.111322	0.971667	0.016296	1.261	0.610	0.017	0.000
comp90752_c0_seq	serine protease	0.423289	0.199444	0.968333	0.169022	0.471	0.006	0.175	0.010
comp82812_c1_seq	hypothetical protein TcasGA2_TC0	2.372555	7.734445	0.964111	4.442222	3.260	< 0.0001	4.608	< 0.0001
comp81078_c0_seq	spermine oxidase-like	0	0	0.961	0			0.000	0.009
comp82808_c0_seq	md-2-related lipid-recognition	0.203956	0.247311	0.914444	0	1.213	0.397	0.000	< 0.0001
comp87582_c0_seq	beta- -glucanase	0.0539	0.066978	0.905	0.0221	1.243	0.423	0.024	< 0.0001
comp28051_c0_seq	catalase	0.390445	0.202556	0.902556	0.597778	0.519	0.010	0.662	0.091
comp87528_c0_seq	laminin beta-2 chain	0.797556	0.324	0.892778	0.572556	0.406	< 0.0001	0.641	0.027
comp86750_c0_seq	syndecan binding protein	0.8726	0.294556	0.891222	0.141111	0.338	0.007	0.158	0.004
comp83827_c0_seq	carbonic anhydrase-related protein	0.253	0.114667	0.870778	0	0.453	< 0.0001	0.000	< 0.0001
comp81117_c0_seq	pancreatic lipase-related protein 2-l	0	0.129	0.869667	0	Infinity	0.003	0.000	0.025
comp73792_c0_seq	chemosensory protein 6	0.177976	0.105254	0.863111	0.346445	0.591	0.138	0.401	0.011
comp86678_c0_seq	serine protease inhibitor partial	0.311211	0.971444	0.859967	0.303222	3.121	< 0.0001	0.353	0.032
comp87436_c0_seq	leukotriene a-4 hydrolase	0.382945	0.569667	0.856556	0.653222	1.488	0.012	0.763	0.302
comp88698_c0_seq	PREDICTED: uncharacterized prote	0.410345	0.099244	0.846222	0.026322	0.242	0.000	0.031	< 0.0001
comp82272_c0_seq	c-type lectin	0.189286	0	0.839389	0	0.000	0.001	0.000	0.001
comp84724_c0_seq	beta- -glucan recognition protein 2	0.638444	1.152222	0.837667	0.271667	1.805	0.003	0.324	0.000
comp86718_c0_seq	serine protease	0.358211	0.697222	0.825	0.160916	1.946	0.017	0.195	0.000
comp90659_c0_seq	chitooligosaccharidolytic beta-n-ace	0.418389	0.1437	0.808	0	0.343	0.014	0.000	0.004
comp78977_c0_seq	imp dehydrogenase gmp reductase	0.093522	0.501222	0.784667	0	5.359	< 0.0001	0.000	< 0.0001
comp84167_c0_seq	beat- isoform d	0.233933	0.248111	0.782333	0	1.061	0.761	0.000	0.001
comp74161_c0_seq	deoxyribonuclease i	0.237333	0.357	0.771111	0	1.504	0.124	0.000	0.001
comp82743_c0_seq	lysosomal pro-x carboxypeptidase-l	0.134556	0.021544	0.764445	0	0.160	0.000	0.000	0.000
comp78587_c0_seq	cellular retinoic acid binding protein	0.302922	0.771778	0.761444	1.433333	2.548	0.028	1.882	< 0.0001
comp86206_c1_seq	aldo-keto reductase	0.0563	0.138833	0.754889	0	2.466	< 0.0001	0.000	0.000
comp92122_c0_seq	probable transcription-associated pr	0.166067	0.030567	0.751556	0	0.184	0.020	0.000	0.001

comp90171_c1_seq	reverse transcriptase	1.337667	2.116667	0.744667	0.50679	1.582	0.044	0.681	0.245
comp86764_c0_seq	probable atp-dependent rna helicas	0	0	0.721667	0			0.000	0.012
comp86725_c0_seq	methylenetetrahydrofolate dehydro	0	0	0.712333	0			0.000	< 0.0001
comp86020_c1_seq	chymotrypsin	0.15986	0.013356	0.709778	0	0.084	0.003	0.000	< 0.0001
comp90740_c0_seq	proactivator polypeptide	0.231889	0.075967	0.708333	0.0741	0.328	< 0.0001	0.105	< 0.0001
comp86727_c0_seq	beat protein	0.718733	0.149433	0.695445	0.087944	0.208	0.001	0.126	0.003
comp81666_c0_seq	hypothetical protein KGM_13675	0.073757	0.175222	0.689	0	2.376	< 0.0001	0.000	< 0.0001
comp89702_c0_seq	lanb2	1.208889	0.658667	0.684556	0.758222	0.545	0.001	1.108	0.556
comp84154_c0_seq	serine proteinase	0.070511	0	0.674444	0	0.000	0.002	0.000	0.004
comp87524_c1_seq	peptidoglycan recognition protein	0.479256	0.545	0.671778	0.110556	1.137	0.538	0.165	< 0.0001
comp88597_c0_seq	arylsulfatase j-like	0.375067	0.404889	0.670667	0.228889	1.080	0.818	0.341	0.007
comp89163_c0_seq	lysosomal alpha-mannosidase (ma	0.263022	0.222222	0.667333	0.036456	0.845	0.373	0.055	< 0.0001
comp84867_c0_seq	adhesion related transmembrane	0.215268	0.310889	0.656778	0	1.444	0.086	0.000	< 0.0001
comp90999_c0_seq	autophagy related protein atg3-like	0.735667	0.106933	0.656	0.0753	0.145	< 0.0001	0.115	< 0.0001
comp84192_c0_seq	cg14275 cg14275-pa	0.221567	0.621778	0.655333	0.367667	2.806	0.014	0.561	0.019
comp83499_c0_seq	isocitrate dehydrogenase	0.450111	0.234222	0.648778	0.169778	0.520	0.000	0.262	0.001
comp87870_c0_seq	alpha- partial	0.150284	0.141311	0.639556	0.077744	0.940	0.820	0.122	0.002
comp86490_c1_seq	malate dehydrogenase	1.52	0.246222	0.638556	0.261	0.162	0.000	0.409	0.011
comp89416_c1_seq	neutral alpha-glucosidase ab-like	0.453778	0.777445	0.637456	0.153956	1.713	0.211	0.242	0.029
comp86657_c0_seq	venom dipeptidyl peptidase 4-like	0.450778	0.710889	0.633556	0.398667	1.577	0.003	0.629	0.115
comp83236_c0_seq	aldo-keto reductase	0.828467	1.522889	0.627444	1.017444	1.838	0.115	1.622	0.002
comp82440_c0_seq	ubiquitin family protein	0.289956	0.164222	0.625667	0.201056	0.566	0.111	0.321	0.008
comp88368_c0_seq	ankyrin repeat domain protein	0	0.081956	0.622667	0.103111	Infinity	0.015	0.166	0.017
comp66611_c0_seq	hypothetical protein KGM_18556	0.640889	0.305222	0.614889	0.173333	0.476	0.081	0.282	0.033
comp80424_c0_seq	takeout jhbp like protein	0.0528	0.096156	0.611445	0.153222	1.821	0.140	0.251	< 0.0001
comp83396_c1_seq	hypothetical protein	0.626356	0.159456	0.588445	0.151233	0.255	0.001	0.257	< 0.0001
comp70799_c0_seq	14-3-3 zeta	0.116589	0.198333	0.587556	0.615889	1.701	0.044	1.048	0.881
comp88697_c0_seq	extradiol ring-cleavage dioxygenase	0.139359	0.050956	0.578278	0	0.366	0.019	0.000	0.001
comp82183_c0_seq	serine protease 33	0.155044	0	0.574111	0	0.000	< 0.0001	0.000	< 0.0001
comp83840_c0_seq	glutathione s-transferase omega-1-	0.126289	0.073011	0.573333	0.202511	0.578	0.033	0.353	0.001
comp86286_c0_seq	carboxypeptidase e-like	0.090878	0.128033	0.570333	0.048844	1.409	0.297	0.086	< 0.0001
comp87076_c0_seq	multiple inositol polyphosphate pho	0.715722	0.152778	0.563	0.125178	0.213	0.001	0.222	0.010
comp91245_c0_seq	fibulin 1	0.536556	0.484111	0.548111	0.145056	0.902	0.470	0.265	< 0.0001
comp86868_c0_seq	thrombospondin-4-like isoform x3	0.357	0.265778	0.547889	0.207	0.744	0.164	0.378	0.003

comp85649_c0_seq	c-type lectin 10	0.105373	0.381889	0.535222	0	3.624	0.039	0.000	< 0.0001
comp87309_c0_seq	phosphoglucose mutase	0.297589	0.173667	0.535222	0.223889	0.584	0.057	0.418	0.001
comp86182_c0_seq	venom acid phosphatase	0.303833	0	0.523111	0	0.000	< 0.0001	0.000	0.000
comp78334_c0_seq	c-type lectin	0.231033	0.105611	0.521667	0	0.457	0.053	0.000	0.014
comp88108_c0_seq	pattern recognition serine proteinas	0.296822	0.305778	0.511889	0.198778	1.030	0.888	0.388	0.005
comp90660_c0_seq	membrane-associated guanylate w	0.199122	0	0.505144	0	0.000	0.014	0.000	0.144
comp88977_c0_seq	isoform a	0.228333	0.427111	0.505111	0.110333	1.871	0.013	0.218	0.001
comp89010_c0_seq	heat shock protein 90	0.221556	0.431444	0.461889	0.929667	1.947	< 0.0001	2.013	0.023
comp86270_c0_seq	neuroserpin isoform 2	0.231544	0.153778	0.451556	0.123767	0.664	0.169	0.274	0.010
comp88907_c1_seq	o-glycosyl hydrolase family 30 prote	0	0	0.443333	0			0.000	0.049
comp80295_c0_seq	juvenile hormone binding protein	0	0	0.435333	0			0.000	0.007
comp82218_c0_seq	glutathione s-transferase	0.262222	0.404333	0.434111	1.443333	1.542	0.093	3.325	< 0.0001
comp80492_c0_seq	protein cueball-like	0.054487	0.204667	0.422778	0	3.756	0.000	0.000	0.013
comp91219_c0_seq	arylphorin subunit alpha	0	0	0.412333	0			0.000	0.001
comp82833_c0_seq	cg12811 cg12811-pa	0.087344	0.205944	0.409333	0	2.358	0.073	0.000	< 0.0001
comp84005_c0_seq	hemolymph proteinase 6	0.1743	0.167378	0.406311	0.023822	0.960	0.845	0.059	0.002
comp86258_c1_seq	uncharacterized oxidoreductase yrb	0.2445	0.360778	0.405922	1.369444	1.476	0.078	3.374	< 0.0001
comp82793_c0_seq	glutathione s-transferase	0.191233	0.602	0.404703	0.607778	3.148	0.002	1.502	0.145
comp84512_c0_seq	plasma alpha-l-fucosidase	0.105238	0.085156	0.395	0	0.809	0.264	0.000	0.000
comp88086_c0_seq	beat- isoform a	0.388344	0.063456	0.392767	0.007489	0.163	< 0.0001	0.019	0.003
comp87389_c0_seq	aminoacylase-1-like	0.250922	0.136567	0.364444	0.1211	0.544	0.024	0.332	0.036
comp90967_c0_seq	aminopeptidase n-like protein	0.337445	0.193667	0.362889	0.589667	0.574	0.006	1.625	0.039
comp80492_c1_seq	protein cueball-like	0.056231	0.149867	0.355222	0	2.665	0.001	0.000	< 0.0001
comp80563_c0_seq	plasma glutamate carboxypeptidase	0.156541	0.077233	0.353556	0.036559	0.493	0.034	0.103	0.004
comp83983_c2_seq	histone h2a	0.02619	0.070822	0.351111	0.517333	2.704	0.010	1.473	0.060
comp87423_c0_seq	cysteine proteinase inhibitor precurs	0.428778	0.082189	0.342456	0.012678	0.192	0.001	0.037	0.001
comp86854_c0_seq	isoform b	0.148833	0	0.335556	0	0.000	0.004	0.000	0.015
comp82573_c0_seq	peroxiredoxin 1	0.124122	0.414889	0.321222	0.788889	3.343	< 0.0001	2.456	< 0.0001
comp90473_c0_seq	unconventional myosin-ixa-like	0	0	0.318889	0			0.000	0.024
comp90203_c0_seq	calsyntenin-1 precursor	0.185389	0.040167	0.318222	0	0.217	0.000	0.000	0.009
comp91407_c0_seq	receptor tyrosine phosphatase type	0.0252	0	0.318167	0	0.000	< 0.0001	0.000	0.001
comp85815_c0_seq	fatty acid synthase	0.186667	0.0514	0.317333	1.576333	0.275	0.008	4.967	0.001
comp84657_c0_seq	serine protease 2	0.018733	0	0.314	0	0.000	0.007	0.000	< 0.0001
comp82633_c0_seq	PREDICTED: uncharacterized prote	1.044839	0.365556	0.313111	0.457111	0.350	0.047	1.460	0.291

comp90656_c0_seq	apyrase	0.365978	0.391222	0.301889	0.063211	1.069	0.816	0.209	0.012
comp83157_c1_seq	purple acid phosphatase	0	0	0.296067	0			0.000	0.001
comp86799_c0_seq	hypothetical protein KGM_06662	0.412111	0.049322	0.290711	0	0.120	< 0.0001	0.000	0.000
comp86427_c0_seq	palmitoyl-protein thioesterase 1	0.009051	0	0.277133	0	0.000	0.043	0.000	0.002
comp85443_c0_seq	superoxide dismutase	0.281333	0.357111	0.272245	0.727222	1.269	0.241	2.671	0.001
comp90259_c1_seq	collagen alpha-1 chain-like	1.734667	0.057123	0.271667	0.0843	0.033	< 0.0001	0.310	0.053
comp28127_c0_seq	profilin	0.137044	0.217222	0.27	0.417444	1.585	0.036	1.546	0.150
comp77144_c0_seq	odorant-binding protein	0.088366	0.310733	0.259211	0.552978	3.516	0.000	2.133	0.099
comp77886_c0_seq	hmct_bommo ame: full=hemocytin	0.513889	0.046122	0.249056	0.052074	0.090	0.070	0.209	0.037
comp84437_c0_seq	takeout jhbp like protein	0	0.041467	0.242556	0	Infinity	0.001	0.000	0.010
comp82847_c0_seq	PREDICTED: uncharacterized prote	0.286044	0.498556	0.238333	0.106933	1.743	0.052	0.449	0.020
comp93298_c0_seq	bilin binding protein 1	0.242911	0.460445	0.235856	0.523667	1.896	0.067	2.220	0.015
comp81818_c1_seq	tubulin alpha-1 chain-like	0	0	0.222222	0			0.000	0.026
comp86956_c0_seq	fructose -bisphosphate aldolase	0	0.043144	0.221711	0.086167	Infinity	0.003	0.389	0.136
comp74143_c0_seq	ommochrome binding protein 2	0.194444	0.374778	0.216289	0.312444	1.927	0.006	1.445	0.202
comp88391_c0_seq	venom serine carboxypeptidase-like	0.029139	0	0.215878	0	0.000	0.001	0.000	0.016
comp88591_c0_seq	transketolase-like protein 2	0.259711	0.137333	0.213333	0.735667	0.529	0.010	3.448	< 0.0001
comp84057_c0_seq	ecdysteroid regulated protein	0	0	0.211078	0			0.000	0.001
comp91561_c0_seq	isoform a	0.674333	0.089611	0.210667	0.070756	0.133	< 0.0001	0.336	0.027
comp82743_c1_seq	lysosomal pro-x carboxypeptidase	0	0	0.202778	0			0.000	0.007
comp90203_c0_seq	calsyntenin-1-like	0.167922	0	0.193778	0	0.000	< 0.0001	0.000	0.017
comp69917_c0_seq	neurexin domain-containing protein	0.404211	0.467222	0.186222	0.034867	1.156	0.744	0.187	0.037
comp86587_c0_seq	neural cell adhesion molecule 1-like	0.2291	0.114711	0.175111	0.022989	0.501	0.021	0.131	0.009
comp90517_c0_seq	fibrillin-2	0.335111	0	0.169	0.025644	0.000	0.000	0.152	0.023
comp86072_c0_seq	s-adenosyl-l-homocysteine hydrolas	0.156467	0.1451	0.166667	0.373333	0.927	0.648	2.240	0.044
comp91753_c0_seq	triosephosphate isomerase	0.069178	0.195778	0.166478	0.441	2.830	0.003	2.649	0.001
comp84037_c0_seq	phosphatidylethanolamine-binding p	0.129767	0.219222	0.165333	0.501	1.689	0.153	3.030	0.000
comp79817_c0_seq	40s ribosomal protein sa-like	0.076378	0.055844	0.162222	0.465	0.731	0.103	2.866	0.001
comp88303_c0_seq	transcriptional adapter 1-like	0.019606	0	0.161633	0	0.000	0.025	0.000	0.044
comp85042_c0_seq	ras-related protein rab-35-like	0.081444	0	0.148111	0	0.000	0.001	0.000	0.005
comp88260_c0_seq	PREDICTED: uncharacterized prote	0.065009	0.081367	0.144011	0	1.252	0.334	0.000	0.001
comp87440_c0_seq	c-1-tetrahydrofolate cytoplasmic-like	0.095189	0.078767	0.1419	0.232333	0.827	0.336	1.637	0.036
comp86901_c0_seq	serine hydroxymethyltransferase	0.040709	0.019867	0.136	0.136158	0.488	0.020	1.001	0.998
comp91427_c0_seq	latrophilin cirl-like isoform 1	0.681222	0.076789	0.128	0.040133	0.113	< 0.0001	0.314	0.078

comp85923_c0_seq	hypothetical protein KGM_03936	0.1276	0.0646	0.124333	0	0.506	0.012	0.000	0.007
comp83274_c0_seq	takeout jhbp like protein	0.090856	0.119078	0.1207	0.286667	1.311	0.384	2.375	0.009
comp90967_c0_seq	aminopeptidase n-like	0.214889	0.143889	0.115778	0.476444	0.670	0.008	4.115	< 0.0001
comp71103_c0_seq	low quality protein: basement meml	0.437778	0.438889	0.115222	0.778333	1.003	0.983	6.755	< 0.0001
comp88265_c0_seq	beta-glucosidase precursor	0.08497	0	0.113744	0	0.000	< 0.0001	0.000	0.123
comp79271_c0_seq	glutathione s-transferase 1	0.070822	0.114056	0.106556	0.450444	1.610	0.055	4.227	< 0.0001
comp90644_c0_seq	low density lipoprotein	0.189556	0.121767	0.105444	0.245667	0.642	0.000	2.330	< 0.0001
comp88615_c0_seq	alpha-n-acetyl glucosaminidase	0.335445	0.618556	0.103211	0.078779	1.844	< 0.0001	0.763	0.706
comp73258_c0_seq	arginine kinase	0.075211	0.438111	0.102033	0.367778	5.825	< 0.0001	3.604	< 0.0001
comp85240_c0_seq	serine protease	0.273278	0.111411	0.091311	0.105778	0.408	0.008	1.158	0.705
comp79814_c0_seq	fatbody protein 3rev-g1	0.098011	0.148333	0.086578	0.118556	1.513	0.009	1.369	0.479
comp90578_c0_seq	peroxidase-like isoform 2	0.014828	0	0.085222	0	0.000	< 0.0001	0.000	0.007
comp87769_c0_seq	probable phosphoserine aminotrans	0.080744	0.028611	0.080178	0.076511	0.354	0.002	0.954	0.917
comp81656_c1_seq	aldo-keto reductase	0.058347	0.164344	0.078956	0.195556	2.817	0.012	2.477	0.014
comp80008_c0_seq	hmg176 isoform a	0.042836	0.095233	0.077444	0.051	2.223	< 0.0001	0.659	0.422
comp81705_c0_seq	hmg176 isoform c	0.178867	0.039011	0.069389	0	0.218	0.002	0.000	0.001
comp86780_c0_seq	isoform a	0.371333	0.248667	0.060411	0	0.670	0.008	0.000	0.018
comp81720_c0_seq	ribosomal protein s3a	0.056334	0.100278	0.056533	0.461111	1.780	0.072	8.156	< 0.0001
comp81829_c0_seq	bifunctional purine biosynthesis pro	0.08653	0.034233	0.014556	0.186111	0.396	0.000	12.786	< 0.0001
comp87029_c0_seq	hrp65 protein	5.288889	0.024778	0.002011	0.304245	0.005	0.308	151.282	0.006
comp89223_c0_seq	protein npc2 homolog	0	0.109056	0	0	Infinity	0.018		
comp3229135_c0_s	hypothetical protein KGM_07884	0	0	0	2.497778			Infinity	0.002
comp78016_c0_seq	calponin transgelin	0	0.052058	0	0	Infinity	0.000		
comp72945_c0_seq	protease inhibitor 1	0.033444	0	0	0.018744	0.000	0.006	Infinity	0.068
comp83876_c0_seq	hypothetical protein KGM_12914	0.06979	0.209222	0	0.1084	2.998	0.007	Infinity	< 0.0001
comp88131_c0_seq	cytosolic non-specific dipeptidase-li	0	0	0	0.026847			Infinity	0.019
comp80465_c0_seq	thioredoxin-like protein	0.087464	0.092689	0	0.22108	1.060	0.848	Infinity	0.000
comp61966_c1_seq	REPAT34	0.163244	0.305544	0	0.071333	1.872	0.122	Infinity	0.002
comp83468_c0_seq	fk506-binding protein	0.052722	0	0	0	0.000	0.003		
comp84382_c0_seq	cytochrome c	0	0.0357	0	0.1043	Infinity	0.007	Infinity	< 0.0001
comp89480_c1_seq	selenoprotein m	0.119682	0.088089	0	0.024978	0.736	0.259	Infinity	0.010
comp81420_c0_seq	REPAT33	0.065332	0.152411	0	0	2.333	0.017		
comp69720_c0_seq	ribosomal protein p1	0.090423	0.121378	0	0.311444	1.342	0.151	Infinity	0.001
comp88444_c0_seq	peroxidase-like	0	0.029156	0	0	Infinity	0.001		

comp77537_c1_seq	60s acidic ribosomal protein p2	0	0	0	0.185589			Infinity	0.000
comp78399_c0_seq	cuticular protein rr-1 motif 46	0	0.342489	0	0.099589	Infinity	< 0.0001	Infinity	0.002
comp85056_c2_seq	ribosomal protein l30	0.024856	0	0	0.181678	0.000	< 0.0001	Infinity	< 0.0001
comp85802_c1_seq	histone 3	0	0	0	0.129333			Infinity	0.001
comp70756_c0_seq	ribosomal protein s26	0.015356	0.058278	0	0.216711	3.795	0.048	Infinity	< 0.0001
comp78179_c0_seq	macrophage migration inhibitory fac	0	0	0	0.081622			Infinity	0.012
comp82267_c0_seq	hypothetical protein KGM_01763	0.147898	0	0	0	0.000	0.000		
comp86752_c0_seq	aldo-keto reductase	0.029856	0	0	0	0.000	0.007		
comp27985_c0_seq	ribosomal protein l34	0	0.035489	0	0.080111	Infinity	0.038	Infinity	0.001
comp91655_c0_seq	ribosomal protein s25	0.061691	0.121111	0	0.371889	1.963	0.000	Infinity	< 0.0001
comp81167_c0_seq	hmg176 isoform a	0	0.035278	0	0.012689	Infinity	< 0.0001	Infinity	0.024
comp78411_c0_seq	ribosomal protein l36	0	0	0	0.104156			Infinity	0.000
comp81861_c0_seq	low quality protein: protein cueball-li	0	0	0	0.103			Infinity	0.011
comp61873_c0_seq	ribosomal protein l35	0	0	0	0.114412			Infinity	0.004
comp87482_c0_seq	amine oxidase	0.0203	0	0	0	0.000	0.001		
comp74713_c0_seq	chemosensory protein partial	0	0	0	0.329			Infinity	0.001
comp78446_c0_seq	ribosomal protein l31	0.022557	0.035033	0	0.428778	1.553	0.226	Infinity	< 0.0001
comp83626_c0_seq	fatty acid binding protein	0	0.147056	0	0	Infinity	< 0.0001		
comp61996_c0_seq	waprin-phi1-like isoform 2	0.312667	0.322111	0	0.042933	1.030	0.879	Infinity	0.026
comp81057_c0_seq	vacuolar atp synthase subunit f	0	0.009796	0	0.027622	Infinity	0.019	Infinity	0.007
comp91510_c0_seq	alpha-2-macroglobulin-like protein 1	0.038849	0	0	0	0.000	< 0.0001		
comp79515_c0_seq	chemosensory protein 1 partial	0.221956	0	0	0	0.000	0.002		
comp84659_c0_seq	tpa: cuticle protein	0	0	0	0.136444			Infinity	0.030
comp61773_c0_seq	cg12324 protein	0.039381	0.079922	0	0.351667	2.029	0.095	Infinity	< 0.0001
comp80274_c0_seq	ribosomal protein s30	0	0	0	0.174256			Infinity	< 0.0001
comp73243_c0_seq	thymosin isoform 2	0	0.016104	0	0	Infinity	0.033		
comp47514_c0_seq	ribosomal protein s24	0	0	0	0.1813			Infinity	< 0.0001
comp77463_c0_seq	neuroglian	0.064193	0	0	0	0.000	0.003		
comp78381_c0_seq	ribosomal protein s17	0.023301	0.072189	0	0.468222	3.098	< 0.0001	Infinity	< 0.0001
comp69686_c0_seq	ribosomal protein l27	0	0.036733	0	0.187444	Infinity	0.000	Infinity	< 0.0001
comp75679_c0_seq	ribosomal protein l32	0	0	0	0.049969			Infinity	0.015
comp82171_c0_seq	fatty acid binding protein	0	0.615667	0	0.543222	Infinity	< 0.0001	Infinity	< 0.0001
comp82171_c0_seq	fatty acid binding protein	0	0.411778	0	0.647444	Infinity	< 0.0001	Infinity	< 0.0001
comp83892_c0_seq	fatty acid binding protein	0.010786	0.387	0	0.517444	35.881	< 0.0001	Infinity	< 0.0001

comp83660_c1_seq	fatty acid-binding adipocyte-like	0	0	0	0.015367			Infinity	0.007
comp84033_c0_seq	antennal binding protein	0.137522	0.276778	0	0.327391	2.013	0.004	Infinity	< 0.0001
comp79384_c0_seq	hmg176 isoform c	0.071956	0	0	0	0.000	0.002		
comp81861_c0_seq	hypothetical protein KGM_13045	0.110818	0.425667	0	0	3.841	0.000		
comp73633_c0_seq	ribosomal protein s12	0	0	0	0.197322			Infinity	< 0.0001
comp79650_c0_seq	ribosomal protein l28	0	0	0	0.137778			Infinity	0.001
comp89409_c0_seq	centrosomal protein of 290 kda-like	0	0	0	0.226656			Infinity	0.002
comp89908_c0_seq	aldehyde dehydrogenase family 7 m	0	0	0	0.039633			Infinity	0.029
comp69454_c0_seq	ribosomal protein s23	0	0.039744	0	0.208433	Infinity	< 0.0001	Infinity	< 0.0001
comp82078_c0_seq	hmg176 isoform e	0.099122	0	0	0	0.000	0.001		
comp81213_c0_seq	cg31997 cg31997-pa	0.080344	0	0	0.067222	0.000	0.009	Infinity	0.001
comp85292_c0_seq	probable atp-dependent rna helicas	0.330233	0.0495	0	0	0.150	0.000		
comp70916_c0_seq	ribosomal protein s15	0	0	0	0.158111			Infinity	0.002
comp72945_c0_seq	protease inhibitor 1	0	0.029844	0	0	Infinity	0.024		
comp74087_c0_seq	ribosomal protein l22	0.009723	0.058133	0	0.250867	5.979	< 0.0001	Infinity	< 0.0001
comp82543_c0_seq	odorant-binding protein	0	0.164111	0	0.1256	Infinity	< 0.0001	Infinity	0.003
comp82543_c0_seq	odorant-binding protein	0	0.203922	0	0.115989	Infinity	0.003	Infinity	< 0.0001
comp83319_c0_seq	gloverin-like protein	0	0.049122	0	0	Infinity	0.002		
comp83691_c0_seq	farnesoic acid o-methyl transferase	0	0.022522	0	0	Infinity	0.008		
comp84067_c0_seq	odorant-binding protein	0.858333	1.932444	0	2.791111	2.251	0.042	Infinity	< 0.0001
comp61985_c0_seq	actin-depolymerizing factor 1	0.251827	0.346111	0	0.940778	1.374	0.125	Infinity	< 0.0001
comp70099_c0_seq	ribosomal protein l27a	0	0	0	0.215889			Infinity	0.006
comp73968_c0_seq	probable pterin-4-alpha-carbinolami	0.018918	0	0	0.079178	0.000	0.018	Infinity	< 0.0001
comp75709_c0_seq	ribosomal protein l26	0	0	0	0.079156			Infinity	< 0.0001
comp91705_c0_seq	repressor splicing factor 1	0	0	0	0.076856			Infinity	< 0.0001
comp47494_c0_seq	myosin light chain alkali-like isoform	0	0.103056	0	0	Infinity	< 0.0001		
comp73423_c0_seq	ribosomal protein s16	0.030262	0.053178	0	0.519667	1.757	0.008	Infinity	< 0.0001
comp79852_c0_seq	ribosomal protein s14	0.016609	0.053056	0	0.455445	3.194	0.014	Infinity	< 0.0001
comp80016_c1_seq	ribosomal protein s13	0.013822	0.088378	0	0.286245	6.394	0.000	Infinity	< 0.0001
comp47583_c0_seq	40s ribosomal protein s11	0	0.023741	0	0.204333	Infinity	0.021	Infinity	< 0.0001
comp69758_c0_seq	ribosomal protein s18	0.042669	0.079856	0	0.342778	1.872	0.001	Infinity	< 0.0001
comp70728_c0_seq	nonmuscle myosin essential light ch	0	0	0	0.022911			Infinity	0.001
comp78419_c0_seq	ribosomal protein s19	0	0.023364	0	0.215778	Infinity	0.003	Infinity	< 0.0001
comp88889_c0_seq	niemann-pick type c-	0.071388	0	0	0	0.000	0.002		

comp79260_c0_seq	14 kda phosphohistidine phosphata	0.006577	0	0	0.018189	0.000	0.024	Infinity	0.028
comp83496_c0_seq	lipopolysaccharide-induced tumor n	0.068622	0	0	0	0.000	< 0.0001		
comp47548_c0_seq	ribosomal protein l24	0	0	0	0.162667			Infinity	< 0.0001
comp73249_c0_seq	ribosomal protein s10	0.027525	0.071933	0	0.339444	2.613	0.000	Infinity	< 0.0001
comp90703_c0_seq	hemocyte-specific integrin alpha su	0	0	0	0.035978			Infinity	0.006
comp73794_c0_seq	ribosomal protein l21	0	0	0	0.211778			Infinity	0.000
comp78720_c0_seq	ribosomal protein l35a	0.006739	0.024322	0	0.093222	3.609	0.024	Infinity	0.001
comp78195_c0_seq	eukaryotic translation initiation facto	0	0.025067	0	0.073389	Infinity	0.017	Infinity	< 0.0001
comp87065_c0_seq	26s proteasome non-atpase regulat	0	0	0	0.051733			Infinity	0.001
comp77591_c0_seq	ribosomal protein l12	0.039249	0.0663	0	0.189	1.689	0.037	Infinity	0.001
comp79049_c0_seq	cyclic amp-regulated protein	0	0	0	0.043579			Infinity	0.000
comp4968_c0_seq1	kl-3 gamma dynein heavy chain	0	0	0	2.238778			Infinity	0.039
comp2530993_c0_s	hypothetical protein KGM_18714	0.210478	0.289067	0	0.181222	1.373	0.452	Infinity	0.002
comp77535_c0_seq	ribosomal protein l14	0.036144	0.087467	0	0.274445	2.420	0.053	Infinity	< 0.0001
comp80362_c0_seq	immune-related hdd11	0.074067	0	0	0	0.000	< 0.0001		
comp62814_c0_seq	actin-related protein 2 3 complex su	0	0.012589	0	0	Infinity	0.003		
comp83421_c0_seq	pseudouridine-5 -monophosphatase	0	0.0115	0	0.084433	Infinity	0.083	Infinity	0.013
comp83964_c0_seq	peritrophic membrane chitin binding	0.025022	0	0	0	0.000	0.013		
comp90800_c0_seq	thiosulfate sulfurtransferase rhodan	0.087481	0	0	0	0.000	< 0.0001		
comp781745_c0_se	defective proboscis extension respo	0.009367	0	0	0	0.000	0.024		
comp82746_c1_seq	glutathione s-transferase	0.05395	0.064267	0	0.216778	1.191	0.519	Infinity	< 0.0001
comp90425_c2_seq	immunophilin fkbp46	0	0	0	0.030556			Infinity	0.023
comp89057_c0_seq	lactoylglutathione lyase	0	0	0	0.051711			Infinity	0.001
comp78080_c0_seq	heat shock protein	0	0.084389	0	0.127367	Infinity	< 0.0001	Infinity	< 0.0001
comp80258_c0_seq	proteasome maturation	0	0	0	0.023486			Infinity	0.002
comp83319_c0_seq	antibacterial peptide	0	0.112978	0	0	Infinity	< 0.0001		
comp81117_c0_seq	pancreatic lipase-related protein 2-l	0.055633	0	0	0	0.000	0.001		
comp47657_c0_seq	ribosomal protein l18a	0.012222	0.080767	0	0.372222	6.608	0.001	Infinity	< 0.0001
comp82692_c2_seq	lysozyme-like protein 1	0.3344	0.078578	0	0	0.235	0.001		
comp74009_c0_seq	small heat shock protein	0	0.040811	0	0.1126	Infinity	< 0.0001	Infinity	< 0.0001
comp82233_c0_seq	juvenile hormone diol kinase	0	0.0953	0	0	Infinity	< 0.0001		
comp69644_c0_seq	adp-ribosylation factor 1	0.049132	0.060822	0	0.1217	1.238	0.208	Infinity	0.000
comp61973_c0_seq	ribosomal protein l18	0	0.055344	0	0.318444	Infinity	0.012	Infinity	< 0.0001
comp77455_c0_seq	ras-related protein rap-1b precursor	0.029469	0.029041	0	0.052989	0.985	0.959	Infinity	< 0.0001

comp84010_c0_seq	galectin partial	0	0.033889	0	0.092322	Infinity	0.008	Infinity	< 0.0001
comp85548_c0_seq	protein takeout-like	0.054996	0	0	0	0.000	0.001		
comp88408_c1_seq	dna supercoiling factor	0.053222	0	0	0	0.000	< 0.0001		
comp82597_c0_seq	hypothetical protein KGM_14540	0.009978	0	0	0	0.000	0.007		
comp73447_c0_seq	60s ribosomal protein l17	0.006788	0.047189	0	0.184222	6.952	< 0.0001	Infinity	< 0.0001
comp81640_c0_seq	peroxiredoxin 5	0.035744	0.0175	0	0	0.490	0.009		
comp86291_c0_seq	pacifastin-related peptide precursor	0.091778	0.317556	0	0.054589	3.460	0.013	Infinity	0.004
comp62070_c0_seq	small heat shock protein	0	0.053033	0	0.116556	Infinity	< 0.0001	Infinity	0.001
comp84871_c0_seq	calponin transgelin	0.032489	0.061278	0	0.2022	1.886	0.180	Infinity	0.001
comp61966_c0_seq	ribosomal protein l9	0.018637	0.064656	0	0.183689	3.469	0.005	Infinity	< 0.0001
comp79995_c2_seq	ribosomal protein s7	0.0121	0.048644	0	0.339445	4.020	0.000	Infinity	< 0.0001
comp85106_c1_seq	af361483_1chymotrypsin inhibitor c	0.186667	0.805778	0	0.071011	4.317	0.038	Infinity	< 0.0001
comp69576_c0_seq	ras-like gtp-binding protein rho1	0.070833	0.063744	0	0.066733	0.900	0.637	Infinity	0.001
comp84508_c0_seq	ras-related protein rac1	0.031833	0.029956	0	0.032111	0.941	0.907	Infinity	< 0.0001
comp84459_c0_seq	gtp-binding protein sar1	0	0.039467	0	0.103067	Infinity	< 0.0001	Infinity	< 0.0001
comp75402_c0_seq	ribosomal protein s9	0	0.068211	0	0.490333	Infinity	0.002	Infinity	< 0.0001
comp90582_c0_seq	apolipoprotein d-like	0.073511	0	0	0	0.000	0.000		
comp83895_c0_seq	glutathione peroxidase	0	0.2289	0	0.116278	Infinity	0.256	Infinity	0.001
comp79229_c0_seq	ribosomal protein l19	0	0.023111	0	0.395	Infinity	0.012	Infinity	0.001
comp90508_c0_seq	ras-like protein 2-like	0	0	0	0.008689			Infinity	0.028
comp81163_c1_seq	ras-related protein rab-1a-like	0.069767	0.034578	0	0.279	0.496	0.217	Infinity	< 0.0001
comp62115_c1_seq	ribosomal protein l13a	0	0.051467	0	0.2078	Infinity	0.012	Infinity	< 0.0001
comp73487_c0_seq	ribosomal protein l15	0	0.051844	0	0.2152	Infinity	0.001	Infinity	< 0.0001
comp85179_c2_seq	glutathione s-transferase	0	0	0	0.508778			Infinity	0.006
comp77814_c0_seq	proteasome subunit beta type-2-like	0	0	0	0.105178			Infinity	< 0.0001
comp80050_c0_seq	apolipoprotein d-like	0	0.051022	0	0.0507	Infinity	< 0.0001	Infinity	0.002
comp81852_c0_seq	glutathione s-transferase	0	0	0	0.034511			Infinity	0.013
comp84212_c0_seq	attacin	0	0.071122	0	0	Infinity	< 0.0001		
comp27984_c0_seq	ribosomal protein s8	0.023927	0.084933	0	0.332222	3.550	0.009	Infinity	< 0.0001
comp80069_c0_seq	ras-related protein rab-2a	0	0	0	0.057789			Infinity	0.001
comp81216_c0_seq	udp-glucose pyrophosphatase	0.019514	0	0	0	0.000	0.009		
comp84810_c0_seq	glutathione s-transferase	0.089372	0	0	0.221889	0.000	0.000	Infinity	0.024
comp86621_c0_seq	nascent polypeptide associated con	0	0	0	0.071111			Infinity	< 0.0001
comp72561_c0_seq	ras-related protein rab-11a-like	0.046356	0.073356	0	0.168378	1.582	0.082	Infinity	< 0.0001

comp76772_c0_seq	gtp-binding nuclear protein ran	0.0502	0.059533	0	0.084256	1.186	0.574	Infinity	0.001
comp84035_c0_seq	cytidylate kinase	0	0.061767	0	0.049478	Infinity	0.000	Infinity	0.000
comp84810_c1_seq	glutathione s-transferase	0	0	0	0.360889			Infinity	< 0.0001
comp86356_c1_seq	adenylyl cyclase-associated protein	0	0	0	0.014733			Infinity	0.001
comp79180_c0_seq	eukaryotic translation initiation facto	0	0	0	0.068667			Infinity	0.006
comp79945_c0_seq	fk506-binding protein	0.069344	0	0	0.112178	0.000	< 0.0001	Infinity	< 0.0001
comp82070_c0_seq	46 kda fk506-binding nuclear protei	0	0	0	0.016278			Infinity	0.024
comp88925_c0_seq	spondin-2-like isoform 1	0	0.040411	0	0	Infinity	0.024		
comp27991_c0_seq	rna-binding protein squid-like isofor	0	0.0116	0	0.176289	Infinity	0.010	Infinity	< 0.0001
comp73704_c0_seq	ribosomal protein l10a	0.006869	0.049089	0	0.244111	7.147	0.000	Infinity	< 0.0001
comp90604_c1_seq	heat shock 70 kda protein cognate	0	0	0	0.024802			Infinity	0.010
comp77551_c0_seq	ribosomal protein s5	0.042112	0.070967	0	0.534222	1.685	0.057	Infinity	< 0.0001
comp78328_c0_seq	ribosomal protein l10	0	0.0319	0	0.143411	Infinity	0.001	Infinity	0.002
comp81916_c0_seq	glutathione s-transferase	0	0	0	0.100144			Infinity	< 0.0001
comp83420_c0_seq	tetraspanin e118	0	0.097467	0	0	Infinity	< 0.0001		
comp84452_c1_seq	glutathione s-transferase	0	0.084944	0	0.207045	Infinity	< 0.0001	Infinity	0.001
comp73852_c0_seq	ribosomal protein l13	0.030317	0.074011	0	0.377778	2.441	0.008	Infinity	< 0.0001
comp91365_c0_seq	PREDICTED: uncharacterized prote	0.024023	0	0	0	0.000	0.022		
comp84128_c1_seq	glutathione s-transferase epsilon 2	0	0.036222	0	0.0465	Infinity	< 0.0001	Infinity	0.000
comp80737_c0_seq	peroxiredoxin 6	0	0.013242	0	0	Infinity	0.000		
comp62653_c0_seq	ribulose-phosphate 3-epimerase-lik	0.016871	0	0	0.0669	0.000	0.006	Infinity	< 0.0001
comp83162_c0_seq	trypsin-like serine proteinase t26	0.078736	0	0	0	0.000	0.001		
comp82514_c0_seq	molting carboxypeptidase a	0.120073	0	0	0	0.000	0.002		
comp87702_c0_seq	glucose-1-phosphatase	0.445333	0.420778	0	0.084278	0.945	0.856	Infinity	0.002
comp27960_c0_seq	proteasome subunit beta type-1	0.011728	0	0	0.121622	0.000	0.020	Infinity	< 0.0001
comp81166_c0_seq	chlorophyllide a binding protein prec	0	0.022178	0	0	Infinity	0.029		
comp82085_c0_seq	multisynthetase auxiliary	0	0	0	0.021433			Infinity	0.026
comp83050_c0_seq	apolipoprotein d-like	0.050933	0	0	0	0.000	< 0.0001		
comp85583_c1_seq	ubiquitin-like protein smt3	0.109578	0.117667	0	0.1353	1.074	0.864	Infinity	< 0.0001
comp79873_c0_seq	glutathione s-transferase 1-like	0	0	0	0.038344			Infinity	0.035
comp82081_c0_seq	proteasome subunit alpha type-2-lik	0.013094	0	0	0.052478	0.000	0.007	Infinity	0.002
comp83817_c0_seq	dihydropteridine reductase	0.090133	0.098467	0	0.304778	1.092	0.686	Infinity	< 0.0001
comp82162_c0_seq	calcyclin-binding protein	0	0.0183	0	0.079989	Infinity	< 0.0001	Infinity	0.000
comp90788_c0_seq	ribose-5-phosphate isomerase	0.013509	0	0	0.068078	0.000	0.005	Infinity	0.002

comp78261_c0_seq	elongation factor 1 delta	0	0.0285	0	0.089833	Infinity	0.001	Infinity	< 0.0001
comp81046_c0_seq	control protein hctl023	0.162	0.203489	0	0.305778	1.256	0.445	Infinity	< 0.0001
comp82036_c0_seq	gasp precursor	0.016189	0.024378	0	0.056156	1.506	0.358	Infinity	< 0.0001
comp88925_c0_seq	spon2b protein	0.197222	0	0	0	0.000	< 0.0001		
comp75881_c0_seq	osiris 9	0	0.020444	0	0	Infinity	0.011		
comp84437_c0_seq	takeout jhbp like protein	0.039089	0	0	0.111556	0.000	0.065	Infinity	0.001
comp78694_c0_seq	juvenile hormone binding protein	0	0	0	0.0453			Infinity	0.001
comp85190_c0_seq	takeout jhbp like protein	0	0	0	0.029044			Infinity	< 0.0001
comp72043_c0_seq	small glutamine-rich tetratricopeptic	0.003109	0	0	0.022178	0.000	0.127	Infinity	0.002
comp79223_c0_seq	ribosomal protein s3	0.069217	0.153233	0	0.420222	2.214	0.006	Infinity	< 0.0001
comp80236_c0_seq	protein takeout-like	0.025167	0	0	0	0.000	0.000		
comp85232_c0_seq	proteasome subunit alpha type-5	0.003868	0.0301	0	0.177511	7.782	0.030	Infinity	0.000
comp87445_c0_seq	proteasome activator complex subu	0.011353	0	0	0.057122	0.000	0.014	Infinity	0.001
comp75805_c0_seq	proteasome subunit alpha type-6	0	0.030867	0	0.154467	Infinity	0.000	Infinity	< 0.0001
comp83397_c0_seq	v-type proton atpase subunit d 1-like	0	0.036044	0	0.076756	Infinity	< 0.0001	Infinity	< 0.0001
comp88494_c0_seq	2-cys peroxiredoxin	0.059267	0.108356	0	0.272333	1.828	0.094	Infinity	< 0.0001
comp80760_c0_seq	takeout jhbp like protein	0.026889	0	0	0	0.000	0.005		
comp80881_c0_seq	proteasome beta-subunit	0.01443	0.049478	0	0.231444	3.429	< 0.0001	Infinity	< 0.0001
comp78012_c0_seq	15-hydroxyprostaglandin dehydroge	0.01257	0	0	0.115278	0.000	0.005	Infinity	< 0.0001
comp82890_c0_seq	takeout jhbp like protein	0.073948	0.022457	0	0	0.304	0.005		
comp84904_c0_seq	chondroitin proteoglycan-2-like	0	0.039589	0	0	Infinity	< 0.0001		
comp83878_c0_seq	hydroxybutyrate dehydrogenase	0	0.15	0	0	Infinity	0.004		
comp83998_c0_seq	circadian clock-controlled	0.1408	0	0	0.026	0.000	0.000	Infinity	0.007
comp88010_c0_seq	isoform b	0	0.0598	0	0	Infinity	0.002		
comp69359_c0_seq	ribosomal protein s6	0.031144	0.066889	0	0.426333	2.148	0.001	Infinity	< 0.0001
comp80386_c0_seq	3-hydroxyacyl- dehydrogenase	0	0	0	0.013839			Infinity	0.005
comp84603_c0_seq	protein creg1-like	0.066057	0	0	0	0.000	0.000		
comp84896_c0_seq	proteasome subunit alpha type-3	0	0.029	0	0.352333	Infinity	0.001	Infinity	0.113
comp84578_c0_seq	flavin reductase-like	0.015278	0	0	0.052689	0.000	0.031	Infinity	< 0.0001
comp74020_c0_seq	ribosomal protein l8	0.016	0.056567	0	0.371667	3.535	0.002	Infinity	< 0.0001
comp82945_c0_seq	proteasome alpha 4 subunit	0	0	0	0.093311			Infinity	< 0.0001
comp85967_c0_seq	frizzled isoform a	0.078236	0	0	0	0.000	0.000		
comp85400_c1_seq	protein takeout-like	0.014244	0	0	0	0.000	0.002		
comp85143_c0_seq	hydroxypyruvate isomerase	0.144433	0.128322	0	0.148556	0.888	0.689	Infinity	< 0.0001

comp79231_c0_seq	ribosomal protein s2	0	0.045344	0	0.245444	Infinity	0.001	Infinity	< 0.0001
comp83126_c0_seq	juvenile hormone binding protein	0.007751	0	0	0	0.000	0.034		
comp83259_c0_seq	fibroin light chain	0.048343	0	0	0	0.000	0.041		
comp28383_c0_seq	14-3-3 protein epsilon-like	0.08	0.190678	0	0.284889	2.383	0.012	Infinity	< 0.0001
comp62089_c0_seq	ribosomal protein l7	0.02621	0.099244	0	0.565	3.787	< 0.0001	Infinity	< 0.0001
comp81502_c0_seq	short-chain dehydrogenease reduct	0.019822	0.0309	0	0.062936	1.559	0.117	Infinity	0.001
comp27988_c0_seq	ribosomal protein s4e	0.024223	0.0595	0	0.513111	2.456	< 0.0001	Infinity	< 0.0001
comp86696_c0_seq	proteasome subunit beta type-4-like	0	0	0	0.081567			Infinity	< 0.0001
comp88993_c0_seq	tetraspanin d107	0.142567	0.093878	0	0.136944	0.658	0.131	Infinity	0.000
comp83745_c0_seq	trypsin-like serine protease	0.012856	0	0	0	0.000	0.027		
comp83400_c0_seq	zinc finger protein 420-like	0.073111	0	0	0	0.000	0.006		
comp86499_c0_seq	sepiapterin reductase-like	0	0.065044	0	0.222022	Infinity	< 0.0001	Infinity	< 0.0001
comp90640_c1_seq	probable g-protein coupled receptor	0.076167	0	0	0	0.000	< 0.0001		
comp80269_c0_seq	ribosomal protein l7a	0.006762	0.028944	0	0.177556	4.280	0.094	Infinity	0.000
comp80777_c0_seq	26s proteasome non-atpase regulat	0	0	0	0.0493			Infinity	< 0.0001
comp77232_c0_seq	isoform a	0.055054	0	0	0.056032	0.000	< 0.0001	Infinity	0.002
comp82914_c0_seq	ommochrome binding protein 1	0.04302	0	0	0	0.000	< 0.0001		
comp88907_c1_seq	glucosylceramidase-like	0.072377	0	0	0	0.000	0.020		
comp79210_c0_seq	control protein hctl025	0	0	0	0.302556			Infinity	< 0.0001
comp81977_c0_seq	beat protein	0.228122	0.064267	0	0	0.282	0.000		
comp83316_c0_seq	phytanoyl- dioxygenase	0	0	0	0.111456			Infinity	< 0.0001
comp88862_c0_seq	pyrroline-5-carboxylate reductase	0.108289	0.080911	0	0.235444	0.747	0.279	Infinity	< 0.0001
comp77206_c0_seq	serine protease 2	0.017044	0	0	0	0.000	0.015		
comp84147_c0_seq	somatomedin-b and thrombospondi	0.122622	0.131433	0	0.066233	1.072	0.842	Infinity	< 0.0001
comp87853_c0_seq	proteasome subunit alpha type-1-lik	0.024308	0.027778	0	0.066	1.143	0.637	Infinity	< 0.0001
comp88430_c0_seq	isoform c	0	0.013078	0	0	Infinity	0.009		
comp28016_c0_seq	eukaryotic translation initiation facto	0	0	0	0.033633			Infinity	0.001
comp87234_c0_seq	sodium potassium-transporting atpa	0.010213	0	0	0	0.000	0.001		
comp77940_c0_seq	proteasome subunit beta 7	0	0	0	0.111411			Infinity	< 0.0001
comp81977_c0_seq	beat protein	0.123556	0	0	0	0.000	0.023		
comp85532_c0_seq	beat protein	0.196078	0.045289	0	0.021289	0.231	0.001	Infinity	0.000
comp86394_c0_seq	glyoxalase domain-containing prote	0.015626	0	0	0	0.000	0.008		
comp87627_c0_seq	low quality protein: neuroglian-like	0.141378	0.075833	0	0.023111	0.536	0.010	Infinity	0.003
comp81695_c0_seq	c3 and pzp-like alpha-2-macroglobu	0.041589	0.088756	0	0.247778	2.134	0.025	Infinity	0.001

comp82662_c0_seq	spermidine synthase	0.0223	0	0	0.025156	0.000	0.016	Infinity	0.007
comp84349_c0_seq	carbonyl reductase	0.011165	0.008267	0	0.036944	0.740	0.577	Infinity	< 0.0001
comp87343_c0_seq	chymotrypsinogen-like protein	0	0.050089	0	0	Infinity	< 0.0001		
comp78495_c0_seq	chymotrypsin-like serine protease	0.018733	0	0	0	0.000	0.008		
comp83320_c0_seq	gtp cyclohydrolase i isoform b	0	0.0543	0	0.324122	Infinity	< 0.0001	Infinity	< 0.0001
comp86069_c0_seq	glycine n-methyltransferase-like	0.022304	0	0	0.073133	0.000	0.001	Infinity	< 0.0001
comp80037_c0_seq	chymotrypsin-like serine protease	0.041311	0	0	0	0.000	0.006		
comp84735_c0_seq	chymotrypsin	0.087957	0.028689	0	0	0.326	0.047		
comp88050_c0_seq	27 kda hemolymph protein	0.081311	0.143144	0	0.093767	1.760	0.068	Infinity	0.002
comp90682_c0_seq	pancreatic triacylglycerol lipase	0.040367	0	0	0.067	0.000	0.002	Infinity	0.023
comp91510_c0_seq	alpha-2-macroglobulin-like protein 1	0.016499	0	0	0	0.000	0.007		
comp78566_c0_seq	ribosomal protein l23a	0	0	0	0.219111			Infinity	< 0.0001
comp81218_c0_seq	c-type lectin 17	0	0.090511	0	0	Infinity	0.000		
comp87445_c0_seq	carboxy-terminal domain rna polym	0.064156	0.0202	0	0	0.315	0.008		
comp78635_c0_seq	ribosomal protein l5	0.016398	0.088067	0	0.215778	5.371	0.001	Infinity	< 0.0001
comp82763_c0_seq	luciferin regenerating enzyme	0	0	0	0.0454			Infinity	0.000
comp83506_c0_seq	n-acetylneuraminate lyase	0.02171	0.045422	0	0.138011	2.092	0.011	Infinity	< 0.0001
comp88131_c1_seq	cytosolic non-specific dipeptidase-li	0	0	0	0.044256			Infinity	0.002
comp81120_c0_seq	c-type lectin	0.0355	0	0	0	0.000	< 0.0001		
comp82889_c0_seq	ubiquinone biosynthesis protein mit	0	0.051011	0	0.047322	Infinity	0.116	Infinity	0.002
comp77458_c0_seq	protein sec13 homolog	0	0	0	0.016356			Infinity	0.023
comp81417_c1_seq	ribosomal protein p0	0.049832	0.133378	0	0.264111	2.677	< 0.0001	Infinity	< 0.0001
comp85800_c0_seq	aldo-keto reductase	0.185667	0.257444	0	0.275456	1.387	0.149	Infinity	< 0.0001
comp91235_c0_seq	carbohydrate sulfotransferase 11-lik	0.072752	0	0	0	0.000	< 0.0001		
comp84194_c0_seq	receptor for activated protein kinase	0.045141	0.064011	0	0.178333	1.418	0.237	Infinity	< 0.0001
comp83460_c0_seq	nad-dependent epimerase dehydrat	0.015328	0	0	0	0.000	0.006		
comp83716_c0_seq	aldo-keto reductase	0.405354	0.266889	0	0.111467	0.658	0.168	Infinity	< 0.0001
comp86297_c0_seq	lambda-crystallin homolog	0.049367	0.034844	0	0.130189	0.706	0.266	Infinity	0.000
comp87955_c0_seq	annexin ix-c	0.0265	0.061111	0	0.041322	2.306	0.002	Infinity	0.004
comp83350_c0_seq	purine nucleoside phosphorylase-lik	0.039839	0	0	0	0.000	0.000		
comp84747_c2_seq	fumarylacetoacetate hydrolase dom	0.024896	0	0	0.023111	0.000	< 0.0001	Infinity	0.008
comp89042_c0_seq	lysosome-associated membrane gly	0.196933	0.043133	0	0.103358	0.219	0.001	Infinity	0.003
comp83601_c0_seq	s-formylglutathione hydrolase	0.039051	0.038778	0	0.061967	0.993	0.985	Infinity	0.002
comp83522_c0_seq	eukaryotic translation initiation facto	0	0	0	0.033856			Infinity	0.011

comp85056_c2_seq	d-3-phosphoglycerate dehydrogena	0.110274	0.060333	0	0.274222	0.547	0.077	Infinity	< 0.0001
comp87164_c0_seq	c-type lectin	1.091111	1.413667	0	0.956667	1.296	0.531	Infinity	0.006
comp86908_c0_seq	neutral lipase	0.058922	0	0	0	0.000	0.000		
comp84096_c1_seq	cytosolic malate dehydrogenase	0.124951	0.072911	0	0.149667	0.584	0.101	Infinity	< 0.0001
comp83520_c1_seq	alcohol dehydrogenase	0.016533	0	0	0.067211	0.000	0.011	Infinity	0.001
comp87134_c0_seq	26s proteasome non-atpase regulat	0	0	0	0.067011			Infinity	0.002
comp87403_c1_seq	transaldolase	0.06306	0.071967	0	0.217667	1.141	0.620	Infinity	< 0.0001
comp88174_c0_seq	seminal fluid protein hacp058	2.67	6.672222	0	3.304444	2.499	0.016	Infinity	< 0.0001
comp84693_c0_seq	eukaryotic translation initiation facto	0	0	0	0.029708			Infinity	0.000
comp71428_c0_seq	inorganic pyrophosphatase	0.065093	0.065922	0	0.123533	1.013	0.969	Infinity	< 0.0001
comp86752_c0_seq	aldo-keto reductase	0	0.133911	0	0.227333	Infinity	0.001	Infinity	< 0.0001
comp72963_c0_seq	activator of 90 kda heat shock prote	0.005611	0	0	0	0.000	0.023		
comp86487_c0_seq	proteasome partial	0	0	0	0.052333			Infinity	< 0.0001
comp86519_c0_seq	phosphoribosyl-pyrophosphate synt	0.015178	0	0	0.125656	0.000	0.023	Infinity	< 0.0001
comp83080_c0_seq	zinc-containing alcohol dehydrogen	0	0	0	0.048156			Infinity	< 0.0001
comp86067_c0_seq	mutant cadherin	0	0.034644	0	0	Infinity	0.031		
comp88392_c0_seq	torso-like protein	0.010711	0	0	0	0.000	0.008		
comp78310_c0_seq	isoform a	0	0.004391	0	0.102756	Infinity	0.040	Infinity	< 0.0001
comp82459_c0_seq	phosphoribosyl pyrophosphate synt	0	0	0	0.015121			Infinity	0.017
comp89059_c1_seq	aggrecan core	0	0.0738	0	0	Infinity	0.004		
comp89233_c1_seq	glycerol-3-phosphate dehydrogenas	0.039427	0.103233	0	0.032856	2.618	0.293	Infinity	0.000
comp87106_c0_seq	cuticle protein cpr149	0	0.008633	0	0	Infinity	0.006		
comp84056_c0_seq	sorbitol dehydrogenase	0.0308	0	0	0	0.000	0.007		
comp84517_c0_seq	trypsin-like serine proteinase t26	0.039967	0	0	0	0.000	< 0.0001		
comp90400_c0_seq	amine oxidase	0.379889	0	0	0.191444	0.000	0.023	Infinity	0.006
comp90408_c0_seq	h aca ribonucleoprotein complex su	0	0	0	0.035678			Infinity	< 0.0001
comp61989_c0_seq	heterogeneous nuclear ribonucleop	0	0	0	0.0186			Infinity	0.042
comp86956_c0_seq	fructose -bisphosphate aldolase	0.117344	0	0	0	0.000	0.001		
comp82782_c0_seq	lipoic acid synthase	0	0.196111	0	0	Infinity	0.000		
comp79690_c0_seq	gamma-glutamyl hydrolase	0.103033	0.138722	0	0.073178	1.346	0.321	Infinity	0.000
comp84887_c0_seq	fibril-forming collagen alpha chain-li	0.013936	0.090078	0	0.147222	6.464	0.001	Infinity	< 0.0001
comp84510_c0_seq	serpin 2	0.100356	0.145556	0	0.0847	1.450	0.242	Infinity	0.000
comp86703_c0_seq	l-allo-threonine aldolase	0	0.018911	0	0.042533	Infinity	0.009	Infinity	0.000
comp84513_c0_seq	glycerophosphoryl diester periplasm	0.317378	0.303333	0	0.194989	0.956	0.789	Infinity	0.001

comp83772_c0_seq	proliferation-associated protein 2g4	0.041344	0	0	0.063933	0.000	< 0.0001	Infinity	< 0.0001
comp79703_c0_seq	nucleosome assembly protein isofo	0	0	0	0.089611			Infinity	< 0.0001
comp76992_c0_seq	chaperonin subunit 4 delta	0	0.014002	0	0.068644	Infinity	0.008	Infinity	< 0.0001
comp85527_c0_seq	eukaryotic translation initiation facto	0	0	0	0.082833			Infinity	< 0.0001
comp87174_c0_seq	26s proteasome non-atpase regulat	0	0	0	0.048044			Infinity	0.000
comp87963_c0_seq	antennal esterase cxe10	0	0.047011	0	0	Infinity	0.000		
comp87396_c0_seq	aliphatic nitrilase	0.097044	0.043267	0	1.002556	0.446	0.001	Infinity	< 0.0001
comp83964_c0_seq	peritrophic membrane chitin binding	0.116529	0.161822	0	0.019222	1.389	0.173	Infinity	0.023
comp86444_c0_seq	26s protease regulatory subunit s10	0	0	0	0.066268			Infinity	0.000
comp88570_c0_seq	vacuolar h	0.092696	0	0	0	0.000	0.007		
comp87837_c1_seq	ribonuclease l inhibitor homolog	0	0	0	0.016756			Infinity	0.031
comp84269_c0_seq	calreticulin	0	0	0	0.107467			Infinity	< 0.0001
comp86728_c0_seq	serine protease inhibitor partial	0.049411	0.017233	0	0.036544	0.349	0.001	Infinity	0.003
comp86107_c0_seq	26s protease regulatory subunit 8-li	0	0	0	0.083567			Infinity	< 0.0001
comp88696_c0_seq	mannose-6-phosphate isomerase-li	0.037713	0	0	0	0.000	0.000		
comp89559_c0_seq	yellow-c	0.013192	0	0	0	0.000	0.021		
comp82649_c0_seq	glutamine-oxaloacetic transaminase	0.024831	0	0	0.035811	0.000	0.002	Infinity	< 0.0001
comp89394_c0_seq	alpha-galactosidase alpha-n-acetylg	0.015811	0	0	0	0.000	0.025		
comp87505_c0_seq	ornithine mitochondrial-like	0.039922	0	0	0.025644	0.000	< 0.0001	Infinity	0.008
comp91357_c2_seq	disintegrin and metalloproteinase de	0.031267	0.005811	0	0	0.186	0.004		
comp82105_c0_seq	potassium sodium hyperpolarization	0	0.0116	0	0	Infinity	0.023		
comp82634_c2_seq	ribosomal protein l3	0.043933	0.058778	0	0.313556	1.338	0.451	Infinity	< 0.0001
comp89780_c1_seq	c1a cysteine protease precursor	1.117333	0.379556	0	0	0.340	< 0.0001		
comp81820_c0_seq	26s protease regulatory subunit 6b	0	0	0	0.041922			Infinity	0.000
comp83089_c0_seq	atp synthase subunit mitochondrial-	0	0	0	0.0103			Infinity	0.026
comp84275_c0_seq	phosphoglycerate kinase	0.1017	0.103522	0	0.197778	1.018	0.939	Infinity	< 0.0001
comp88616_c0_seq	alpha-amylase	0.092067	0.142667	0	0.165044	1.550	0.171	Infinity	0.000
comp80026_c2_seq	eukaryotic initiation factor 4a	0.069044	0.040611	0	0.101011	0.588	0.104	Infinity	< 0.0001
comp79326_c0_seq	t-complex protein 1 subunit delta-lik	0.007626	0.037111	0	0.1919	4.867	0.000	Infinity	< 0.0001
comp86250_c0_seq	PREDICTED: uncharacterized prote	0.053411	0	0	0	0.000	0.022		
comp79910_c0_seq	carboxypeptidase b precursor	0.0089	0	0	0	0.000	0.006		
comp85000_c1_seq	synaptic vesicle membrane protein	0	0	0	0.042067			Infinity	0.001
comp87155_c0_seq	yellow- isoform a	0	0	0	0.017656			Infinity	0.015
comp87212_c0_seq	synaptotagmin 1	0.00945	0	0	0	0.000	0.041		

comp81026_c0_seq	ribosomal protein l4	0.010477	0.082411	0	0.359778	7.866	0.012	Infinity	< 0.0001
comp86676_c0_seq	protein yellow-like	0.018878	0	0	0	0.000	< 0.0001		
comp88513_c2_seq	lachesin-like isoform 2	0.074189	0	0	0	0.000	< 0.0001		
comp85033_c0_seq	26s protease regulatory subunit 6a	0	0	0	0.090722			Infinity	< 0.0001
comp84570_c0_seq	sucrose-6-phosphate hydrolase	0.065212	0	0	0	0.000	< 0.0001		
comp85867_c0_seq	aminoacylase-1a-like isoform x1	0.026127	0	0	0	0.000	0.042		
comp88337_c0_seq	26s protease regulatory subunit 7	0	0	0	0.028389			Infinity	0.000
comp85813_c0_seq	isoform a	0.075161	0	0	0.027178	0.000	< 0.0001	Infinity	0.009
comp88730_c0_seq	iron zinc purple acid phosphatase-li	0.034667	0	0	0	0.000	0.009		
comp79297_c0_seq	26s protease regulatory subunit 4-li	0	0	0	0.0476			Infinity	< 0.0001
comp89783_c0_seq	leucine-rich repeat-containing prote	0.029989	0	0	0	0.000	0.002		
comp78851_c0_seq	rab gdp-dissociation inhibitor	0.0837	0.066956	0	0.105644	0.800	0.420	Infinity	< 0.0001
comp87524_c2_seq	argininosuccinate synthase	0.044333	0	0	0.258022	0.000	0.005	Infinity	0.042
comp90955_c0_seq	cop9 signalosome complex subunit	0	0	0	0.286667			Infinity	0.002
comp86462_c0_seq	eukaryotic translation initiation facto	0	0	0	0.081556			Infinity	0.003
comp82307_c0_seq	yellow-x	0.048693	0.045967	0	0.025056	0.944	0.855	Infinity	0.009
comp84197_c0_seq	pwwp domain-containing protein 2b	0	0	0	0.021344			Infinity	0.022
comp89329_c0_seq	protein notum homolog	0.085778	0	0	0	0.000	< 0.0001		
comp82478_c0_seq	yellow-d	0	0	0	0.1176			Infinity	< 0.0001
comp90604_c0_seq	heat shock protein 70	0.100367	0	0	0.059356	0.000	0.012	Infinity	0.000
comp80293_c2_seq	tubulin alpha-1 chain	0.261633	0.273444	0	0.527111	1.045	0.816	Infinity	< 0.0001
comp85149_c0_seq	26s proteasome non-atpase regulat	0	0	0	0.061967			Infinity	< 0.0001
comp83577_c0_seq	PREDICTED: lachesin-like	0	0.047856	0	0	Infinity	< 0.0001		
comp80120_c0_seq	protein split ends-like	0.055111	0	0	0	0.000	0.023		
comp81744_c0_seq	nadh:ubiquinone na(+)- c subunit	0.305833	0.262522	0	0.138456	0.858	0.670	Infinity	< 0.0001
comp88508_c0_seq	protein unzipped-like	0.049296	0	0	0	0.000	0.000		
comp89425_c0_seq	yellow-f	0.041666	0	0	0	0.000	0.000		
comp90945_c0_seq	fibulin 1	0.074356	0	0	0.063622	0.000	0.002	Infinity	0.008
comp84135_c1_seq	g-protein coupled receptor mth2	0.234833	0.031144	0	0	0.133	< 0.0001		
comp84624_c0_seq	isoform a	0	0	0	0.021949			Infinity	0.012
comp90963_c0_seq	fk506-binding protein 59	0.019532	0	0	0.032278	0.000	0.016	Infinity	0.001
comp83784_c0_seq	vacuolar atp synthase subunit h	0	0	0	0.024422			Infinity	0.007
comp90340_c0_seq	maltose phosphorylase	0	0	0	0.012803			Infinity	0.023
comp85542_c0_seq	selenium-binding protein	0.055388	0.067733	0	0.167456	1.223	0.589	Infinity	< 0.0001

comp90005_c0_seq	equilibrative nucleoside transporter	0.068956	0	0	0	0.000	0.003		
comp91525_c0_seq	cadherin partial	0.039156	0	0	0	0.000	0.000		
comp84894_c1_seq	hexokinase type 2	0.033837	0	0	0	0.000	0.007		
comp87301_c0_seq	6-phosphogluconate dehydrogenas	0.109222	0.105878	0	0.340267	0.969	0.868	Infinity	< 0.0001
comp85334_c0_seq	bleomycin hydrolase	0.236911	0	0	0.049011	0.000	< 0.0001	Infinity	< 0.0001
comp83310_c0_seq	isoform d	0.120444	0.042867	0	0.028389	0.356	0.039	Infinity	< 0.0001
comp82470_c0_seq	protein disulfide-isomerase like prot	0	0	0	0.0483			Infinity	< 0.0001
comp83696_c0_seq	beta- -glucan recognition protein 2	0.135033	0.297111	0	0.131789	2.200	0.006	Infinity	< 0.0001
comp90640_c0_seq	probable g-protein coupled recepto	0.091067	0	0	0	0.000	< 0.0001		
comp81382_c0_seq	adenylosuccinate lyase-like	0	0	0	0.042972			Infinity	< 0.0001
comp84637_c0_seq	probable 26s proteasome non-atpa	0	0	0	0.054422			Infinity	< 0.0001
comp85222_c0_seq	vacuolar h	0	0.066467	0	0.182244	Infinity	< 0.0001	Infinity	< 0.0001
comp85580_c0_seq	protein disulfide isomerase	0.044201	0.084022	0	0.211778	1.901	0.053	Infinity	< 0.0001
comp91533_c0_seq	tolloid-like protein 2-like	0.052878	0	0	0	0.000	< 0.0001		
comp82711_c0_seq	serine protease -like	0.033789	0	0	0	0.000	0.002		
comp83900_c0_seq	venom serine carboxypeptidase-like	0	0.0274	0	0.071511	Infinity	0.010	Infinity	0.013
comp88517_c0_seq	protein eva-1-like	0.083367	0.00588	0	0	0.071	< 0.0001		
comp88987_c0_seq	ccr4-not transcription complex subu	0	0	0	0.068311			Infinity	< 0.0001
comp89711_c0_seq	g protein-coupled receptor	0.021141	0	0	0	0.000	0.030		
comp83319_c0_seq	cysteine synthase	0.012656	0	0	0	0.000	0.001		
comp88483_c0_seq	ugp	0.040862	0.117967	0	0.208556	2.887	0.312	Infinity	< 0.0001
comp79679_c0_seq	aminopeptidase -like	0.064359	0.033022	0	0.055378	0.513	0.007	Infinity	< 0.0001
comp88360_c0_seq	protein singed-like	0	0	0	0.015189			Infinity	0.002
comp83266_c0_seq	inosine-5 -monophosphate dehydro	0.038012	0	0	0.024917	0.000	0.098	Infinity	0.002
comp88583_c0_seq	heparanase-like protein	0.028317	0	0	0	0.000	0.003		
comp95705_c0_seq	adenosine deaminase related grow	0.148322	0	0	0	0.000	< 0.0001		
comp88456_c0_seq	3-oxoacid transferase 1	0	0	0	0.045644			Infinity	0.014
comp86614_c0_seq	g-protein coupled receptor mth2-like	0.04831	0	0	0	0.000	< 0.0001		
comp86452_c0_seq	antitrypsin isoform 2	0.013799	0	0	0	0.000	< 0.0001		
comp89102_c0_seq	pyruvate kinase	0.07272	0.109122	0	0.221667	1.501	0.024	Infinity	< 0.0001
comp90682_c0_seq	pancreatic triacylglycerol lipase	0	0.092389	0	0	Infinity	< 0.0001		
comp88924_c0_seq	probable aminopeptidase npepl1-lik	0.018259	0	0	0.031267	0.000	0.001	Infinity	0.001
comp89530_c0_seq	chaperonin subunit 6a zeta	0	0.050367	0	0.219722	Infinity	0.005	Infinity	0.000
comp89440_c0_seq	arylsulfatase b-like	0.07007	0	0	0	0.000	0.000		

comp89962_c1_seq	t-complex protein 1 subunit beta-lik	0.008578	0.027433	0	0.166111	3.198	0.079	Infinity	< 0.0001
comp88457_c0_seq	transmembrane protein c9orf5-like	0.024115	0	0	0	0.000	0.006		
comp88942_c0_seq	t-complex protein 1 subunit gamma	0.026367	0.102356	0	0.339556	3.882	0.086	Infinity	< 0.0001
comp83821_c0_seq	eukaryotic translation initiation facto	0	0	0	0.069911			Infinity	< 0.0001
comp85806_c0_seq	lipase 3	0.059873	0.293556	0	0.208556	4.903	< 0.0001	Infinity	< 0.0001
comp90657_c1_seq	t-complex protein 1 subunit epsilon-	0	0	0	0.104133			Infinity	< 0.0001
comp89020_c0_seq	t-complex protein 1 subunit eta-like	0	0.048678	0	0.248667	Infinity	< 0.0001	Infinity	< 0.0001
comp84567_c0_seq	t-complex protein 1 subunit theta	0	0.039233	0	0.226778	Infinity	0.003	Infinity	< 0.0001
comp1044034_c0_s	bile salt-activated lipase-like	0	0.024011	0	0	Infinity	0.022		
comp89031_c0_seq	protein tweety-like	0.056428	0	0	0	0.000	< 0.0001		
comp87097_c0_seq	division abnormally delayed	0.040178	0	0	0	0.000	< 0.0001		
comp86885_c0_seq	asparagine synthetase	0.003433	0	0	0.007922	0.000	0.116	Infinity	0.028
comp81089_c0_seq	glutamate dehydrogenase	0	0	0	0.037444			Infinity	< 0.0001
comp87963_c0_seq	antennal esterase cxe10	0.039089	0	0	0	0.000	0.012		
comp88016_c0_seq	t-complex protein 1 subunit alpha-lil	0	0	0	0.211244			Infinity	< 0.0001
comp90112_c0_seq	PREDICTED: uncharacterized prote	0	0.010022	0	0	Infinity	0.007		
comp78869_c0_seq	heat shock protein 70	0	0	0	0.298222			Infinity	0.012
comp86227_c0_seq	antennal esterase cxe5	0	0	0	0.036344			Infinity	0.001
comp86227_c0_seq	antennal esterase cxe5	0.046481	0.068089	0	0.033444	1.465	0.082	Infinity	0.009
comp87141_c2_seq	plexin domain-containing protein 2-l	0.013798	0	0	0	0.000	0.016		
comp77165_c0_seq	dynein intermediate chain ciliary-like	0.086667	0	0	0	0.000	0.006		
comp86867_c0_seq	trehalase 1a	0.167842	0.192556	0	0.324889	1.147	0.391	Infinity	< 0.0001
comp82536_c0_seq	heat shock protein 60	0.039366	0	0	0.150778	0.000	< 0.0001	Infinity	< 0.0001
comp84132_c0_seq	glucose dehydrogenase	0.012844	0	0	0	0.000	0.012		
comp87809_c0_seq	glucose dehydrogenase	0.092477	0	0	0	0.000	0.000		
comp80082_c0_seq	fish- isoform c	0.346	0.077522	0	0.048956	0.224	< 0.0001	Infinity	0.001
comp86084_c0_seq	glucose dehydrogenase	0.017413	0	0	0	0.000	0.003		
comp89763_c0_seq	lysyl-trna synthetase	0	0	0	0.013411			Infinity	0.008
comp84798_c0_seq	phosphoglucose isomerase	0.110693	0.106611	0	0.410222	0.963	0.823	Infinity	< 0.0001
comp85560_c0_seq	phenylalanyl-trna synthetase beta s	0.007127	0	0	0.017289	0.000	0.031	Infinity	0.001
comp81742_c0_seq	protein asteroid-like	0	0.044411	0	0	Infinity	< 0.0001		
comp87961_c0_seq	serine threonine-protein phosphatas	0	0.014044	0	0	Infinity	0.000		
comp90259_c0_seq	collagen alpha-2 chain-like	0.238778	0	0	0	0.000	< 0.0001		
comp90051_c0_seq	disulfide oxidoreductase	0.201544	0.062011	0	0.024689	0.308	0.001	Infinity	0.006

comp87654_c0_seq	ecdysone oxidase	0	0	0	0.013189			Infinity	0.008
comp88102_c0_seq	chitinase	0.018311	0	0	0	0.000	0.022		
comp87496_c0_seq	phosphoglucomutase-2-like	0.009118	0	0	0	0.000	0.028		
comp80318_c0_seq	polyadenylate-binding protein 1-like	0.004401	0	0	0.076189	0.000	0.213	Infinity	0.000
comp90705_c0_seq	cell division cycle and apoptosis reg	0	0	0	0.0075			Infinity	0.033
comp89004_c0_seq	lamin dm0-like	0	0	0	0.040067			Infinity	0.008
comp88503_c0_seq	vacuolar atp synthase catalytic subu	0.017773	0.068811	0	0.223444	3.872	< 0.0001	Infinity	< 0.0001
comp89549_c0_seq	glycosyl hydrolase family 31 protein	0.034504	0	0	0	0.000	0.000		
comp88613_c0_seq	glucose dehydrogenase	0	0.022056	0	0.083111	Infinity	0.009	Infinity	0.001
comp88443_c0_seq	thrombospondin type-1 domain-con	0.045722	0	0	0	0.000	0.000		
comp90177_c0_seq	fimbrin plastin	0	0	0	0.012897			Infinity	0.016
comp82481_c0_seq	xanthine dehydrogenase oxidase	0	0	0	0.008756			Infinity	0.008
comp85739_c0_seq	polycomb protein scm-like	0	0.036411	0	0	Infinity	0.049		
comp82105_c0_seq	i	0.049282	0	0	0	0.000	0.025		
comp86348_c0_seq	heat shock cognate 70 protein	0	0.288	0	0.650444	Infinity	< 0.0001	Infinity	< 0.0001
comp89495_c0_seq	isoform c	0.433333	0.391444	0	0.281889	0.903	0.376	Infinity	< 0.0001
comp89852_c0_seq	tartan capricious-like protein	0.062	0	0	0	0.000	0.024		
comp96043_c0_seq	leucine-rich repeats and immunoglo	0.186133	0.175889	0	0.034411	0.945	0.819	Infinity	0.001
comp91233_c0_seq	#NAME?	0	0	0	0.022552			Infinity	0.000
comp88148_c0_seq	neutral ceramidase-like	0.101733	0.046311	0	0.107578	0.455	0.003	Infinity	< 0.0001
comp88280_c0_seq	sphingomyelin phosphodiesterase	0.051244	0.058811	0	0.072722	1.148	0.317	Infinity	< 0.0001
comp84058_c0_seq	eukaryotic translation initiation facto	0	0	0	0.070567			Infinity	< 0.0001
comp88576_c0_seq	hypothetical protein KGM_14136	0	0.093933	0	0.032056	Infinity	0.006	Infinity	0.040
comp79094_c0_seq	tyrosine-protein phosphatase 10d-li	0.028799	0	0	0	0.000	0.000		
comp85065_c0_seq	acylamino-acid-releasing enzyme-lil	0	0	0	0.016653			Infinity	0.018
comp88625_c0_seq	dipeptidyl peptidase iii	0.098867	0.035111	0	0.166333	0.355	< 0.0001	Infinity	< 0.0001
comp93268_c0_seq	procollagen- -oxoglutarate 5-dioxyg	0	0.0281	0	0	Infinity	0.001		
comp91365_c0_seq	fibril-forming collagen alpha chain-li	0.023344	0	0	0	0.000	0.047		
comp90341_c0_seq	semaphorin-1a-like protein	0.013713	0	0	0	0.000	0.009		
comp86751_c0_seq	metal transporter cnnm2-like	0.053811	0	0	0	0.000	< 0.0001		
comp87469_c0_seq	prolyl endopeptidase	0.030883	0	0	0.068951	0.000	0.007	Infinity	0.021
comp91278_c0_seq	transferrin	0.1344	0.236	0	0.110789	1.756	0.091	Infinity	< 0.0001
comp89797_c0_seq	iron-responsive element-binding pro	0.017833	0	0	0	0.000	0.011		
comp89050_c0_seq	amphoterin-induced protein 2	0.037556	0	0	0	0.000	< 0.0001		

comp90648_c0_seq	elongator complex protein 2	0	0.772	0	0	Infinity	0.015		
comp90184_c1_seq	dnaj homolog subfamily c member	0	0.037933	0	0	Infinity	0.013		
comp90475_c0_seq	fasciclin ii transmembrane isoform	0.127933	0.064556	0	0.03045	0.505	0.002	Infinity	< 0.0001
comp87899_c0_seq	transitional endoplasmic reticulum a	0.049867	0.043556	0	0.248889	0.873	0.425	Infinity	< 0.0001
comp88905_c0_seq	heat shock 70 kda protein 4l-like	0.013067	0	0	0.139767	0.000	0.040	Infinity	< 0.0001
comp90211_c0_seq	isoform c	0.089489	0.0563	0	0.100989	0.629	0.093	Infinity	0.036
comp91586_c0_seq	neurogenic locus notch	0.016711	0	0	0	0.000	0.033		
comp88989_c0_seq	colmedin	0.062167	0.051256	0	0.0422	0.824	0.307	Infinity	0.000
comp88100_c0_seq	isoform b	0.017584	0.055078	0	0.005478	3.132	< 0.0001	Infinity	0.027
comp89392_c0_seq	glycogen phosphorylase	0.050018	0.022511	0	0.071467	0.450	0.233	Infinity	< 0.0001
comp78190_c0_seq	nonclathrin coat protein gamma1-co	0	0	0	0.014267			Infinity	0.002
comp88081_c0_seq	multidrug resistance-associated pro	0	0	0	0.026022			Infinity	0.025
comp91604_c0_seq	hmct_bommo ame: full=hemocytin	0.804667	0.115744	0	0.029489	0.144	0.001	Infinity	0.008
comp71873_c0_seq	long form-like	0	0.021933	0	0	Infinity	0.001		
comp80514_c0_seq	prominin-like protein	0.125133	0	0	0.016027	0.000	< 0.0001	Infinity	0.022
comp89202_c0_seq	beta-mannosidase-like	0	0.013567	0	0	Infinity	0.023		
comp89797_c0_seq	iron-responsive element-binding pro	0	0	0	0.020211			Infinity	0.029
comp92164_c0_seq	ebna2 binding protein p100	0.007224	0	0	0.070389	0.000	0.023	Infinity	< 0.0001
comp91192_c0_seq	sticks and isoform a	0.029067	0	0	0	0.000	0.001		
comp86149_c0_seq	isoform a	0	0	0	0.022297			Infinity	0.020
comp91016_c0_seq	10-formyltetrahydrofolate dehydroge	0.093589	0.082878	0	0.291445	0.886	0.544	Infinity	0.001
comp90652_c0_seq	nucleolar protein 14 homolog	0.091041	0.195444	0	0.761444	2.147	0.001	Infinity	< 0.0001
comp89221_c0_seq	alanyl-trna synthetase	0	0	0	0.004573			Infinity	0.028
comp87118_c0_seq	26s proteasome non-atpase regulat	0	0	0	0.039922			Infinity	< 0.0001
comp91349_c0_seq	voltage-dependent calcium channel	0.210333	0	0	0	0.000	< 0.0001		
comp91330_c0_seq	huntingtin interacting protein 1	0	0.081889	0	0	Infinity	0.024		
comp89236_c0_seq	ubiquitin-activating enzyme e1	0	0	0	0.065603			Infinity	0.002
comp89105_c0_seq	150 kda dynein-associated polypept	0.121433	0.214667	0	0.124444	1.768	0.298	Infinity	0.002
comp91526_c0_seq	phosphoribosylamine-glycine ligase	0.012098	0	0	0	0.000	0.022		
comp91279_c0_seq	atp-citrate synthase-like	0.2461	0.053533	0	1.301444	0.218	0.029	Infinity	0.003
comp82649_c0_seq	importin beta-3	0	0	0	0.0087			Infinity	0.029
comp88838_c0_seq	eukaryotic translation initiation facto	0	0	0	0.083589			Infinity	< 0.0001
comp89960_c0_seq	limkain b1	0.084856	0.023822	0	0	0.281	0.017		
comp91173_c0_seq	structural maintenance of chromoso	0	0	0	0.050432			Infinity	0.046

comp83380_c2_seq	collagen alpha-2 chain	1.197111	0	0	0	0.000	< 0.0001		
comp85327_c0_seq	rna-binding protein 12	0.711222	0	0	0	0.000	< 0.0001		
comp90132_c0_seq	aldehyde oxidase	0.007834	0	0	0	0.000	0.033		
comp90809_c0_seq	extracellular domains-containing pro	0	0.027922	0	0	Infinity	< 0.0001		
comp89868_c0_seq	furin-like protease 2-like	0.108789	0	0	0.012838	0.000	0.005	Infinity	0.002
comp89895_c0_seq	phosphoribosylformylglycinamidine	0	0	0	0.020182			Infinity	< 0.0001
comp88886_c0_seq	tripeptidyl-peptidase 2	0	0	0	0.032167			Infinity	< 0.0001
comp91513_c0_seq	xanthine dehydrogenase	0	0	0	0.025609			Infinity	0.006
comp90493_c0_seq	down syndrome cell adhesion mole	0.027311	0	0	0	0.000	0.002		
comp91526_c0_seq	phosphoribosylamine-glycine ligase	0	0	0	0.035478			Infinity	0.026
comp91603_c0_seq	hemicentin-1-like	0.049122	0	0	0	0.000	< 0.0001		
comp90945_c0_seq	fibulin 1 and	0	0.034522	0	0	Infinity	0.010		
comp91427_c0_seq	latrophilin cirl-like isoform 1	0.681222	0	0	0	0.000	< 0.0001		
comp87765_c0_seq	probable atp-dependent rna helicas	0	10.43778	0	0	Infinity	0.050		
comp91484_c0_seq	bifunctional glutamate proline--trna	0	0.032594	0	0.077267	Infinity	0.259	Infinity	< 0.0001
comp86547_c0_seq	clathrin heavy chain	0	0	0	0.086478			Infinity	< 0.0001
comp91493_c0_seq	isoform partial	0.107867	0.01733	0	0.027144	0.161	< 0.0001	Infinity	0.003
comp86570_c0_seq	myosin heavy isoform d	0	0.333111	0	0	Infinity	0.001		
comp90924_c0_seq	low quality protein: myosin heavy no	0	0	0	0.032389			Infinity	< 0.0001
comp91506_c0_seq	translational activator gcn1	0	0	0	0.008698			Infinity	0.036
comp90478_c0_seq	sortilin-related receptor-like	0.015109	0	0	0.029944	0.000	0.127	Infinity	0.002
comp91619_c0_seq	carbamoyl-phosphate synthase larg	0.044718	0	0	0.034241	0.000	0.006	Infinity	0.000
comp90846_c0_seq	spectrin beta chain	0	0	0	0.019622			Infinity	0.000
comp91571_c0_seq	spectrin alpha chain	0	0	0	0.030967			Infinity	< 0.0001
comp91621_c0_seq	odd oz protein	0.161267	0.0178	0	0	0.110	< 0.0001		
comp91648_c0_seq	muscle-specific protein isoform k	0	0.020822	0	0	Infinity	0.002		
comp91624_c0_seq	dynein heavy chain	0	0	0	0.020856			Infinity	< 0.0001
comp79903_c0_seq	amylase partial	0	0.055011	0	0	Infinity	0.043		
comp91678_c0_seq	ribosomal protein s29	0	0	0	0.148111			Infinity	0.001
comp80529_c0_seq	cecropin b	0	0.066812	0	0	Infinity	0.000		
comp8680_c0_seq1	collagen alpha-2 chain-like	0.387444	0.266	0	0.209767	0.687	0.611	Infinity	0.039
comp84056_c0_seq	sorbitol dehydrogenase	0	0.029067	0	0.082278	Infinity	0.001	Infinity	0.001
comp85583_c1_seq	lipopolysaccharide binding protein	0	0.045089	0	0	Infinity	0.001		
comp91365_c0_seq	PREDICTED: uncharacterized prote	0	0	0	0.012311			Infinity	0.008

comp60971_c0_seq	PREDICTED: uncharacterized prote	0.010889	0	0	0	0.000	0.027		
comp90885_c0_seq	ecdysteroid 22-kinase	0	0.172	0	0	Infinity	0.002		
comp73731_c0_seq	odorant-binding protein	0.114978	0.052622	0	0.029456	0.458	0.122	Infinity	0.001
comp83253_c1_seq	short-chain partial	0	0.020522	0	0	Infinity	0.001		
comp79202_c0_seq	isoform b	0.237889	0	0	0	0.000	0.001		
comp62692_c0_seq	macrophage mannose receptor 1-lil	0.1115	0	0	0	0.000	0.007		
comp70291_c0_seq	ribosomal protein s21	0	0	0	0.073822			Infinity	0.000
comp47327_c0_seq	40s ribosomal protein s27	0	0	0	0.324422			Infinity	< 0.0001
comp75843_c0_seq	acyl- binding protein	0	0.137644	0	0.014456	Infinity	0.000	Infinity	0.033
comp77138_c0_seq	calponin transgelin	0	0.031633	0	0	Infinity	0.032		
comp62047_c0_seq	ribosomal protein l37a	0	0	0	0.029478			Infinity	0.024
comp81679_c0_seq	cysteine rich secreted	0.017949	0.036822	0	0.063567	2.052	0.168	Infinity	0.002
comp83971_c0_seq	diazepam binding inhibitor-like prote	0.07953	0.148033	0	0.221778	1.861	0.068	Infinity	< 0.0001
comp27990_c0_seq	ribosomal protein l37	0	0	0	0.103889			Infinity	0.001
comp82669_c1_seq	diverged serine protease	0.018933	0	0	0	0.000	0.006		
comp85595_c0_seq	organic cation transporter	0	0.022267	0	0	Infinity	0.026		
comp62202_c0_seq	small nuclear ribonucleoprotein e	0	0	0	0.034			Infinity	0.001
comp79251_c0_seq	kazal-type proteinase inhibitor	0.256933	0.1456	0	0.225	0.567	0.461	Infinity	< 0.0001
comp70838_c0_seq	acylphosphatase-2 isoform x1	0	0.016533	0	0.019	Infinity	< 0.0001	Infinity	0.001
