# Supplementary material for: Inhibition of melanization by serpin-5 and serpin-9 promotes baculovirus infection in cotton bollworm Helicoverpa armigera
Source: PLoS Pathog. 2017 Sep 27;13(9):e1006645. doi: 10.1371/journal.ppat.1006645 (PMC5633200; doi:10.1371/journal.ppat.1006645)
Supplement: S4 Table — (PDF) [file ppat.1006645.s011.pdf]

Supplementary Data 4. Repertoire of baculovirus-regulated immune protein in hemolymph

Assembly No.	Name	48hM	48hI	72hM	72hI	48hI/48hM	48hI/48hM Ttest	72hI/72hM	72hI/72hM Ttest
comp88108_c0_seq2	SP42	0.2968222	0.3057778	0.5118889	0.1987778	1.030	0.888	0.388	0.005
comp84724_c0_seq1	βGRP1	0.6384444	1.152222	0.8376667	0.2716667	1.805	0.003	0.324	0.000
comp83696_c0_seq1	βGRP2a	0.1350333	0.2971111	0	0.1317889	2.200	0.006	Infinity	< 0.0001
comp86474_c0_seq1	βGRP2b	2.901889	2.376667	10.86111	1.055556	0.819	0.395	0.097	< 0.0001
comp87582_c0_seq1	βGRP4	0.0539	0.0669778	0.905	0.0221	1.243	0.423	0.024	< 0.0001
comp87524_c1_seq1	PGRP-SA	0.4792556	0.545	0.6717778	0.1105556	1.137	0.538	0.165	< 0.0001
comp91604_c0_seq6	Hemocytin	2.226667	0.1741111	3.242556	0.3895555	0.078	0.000	0.120	0.077
comp78334_c0_seq2	CTL2	0.2310333	0.1056111	0.5216666	0	0.457	0.053	0.000	0.014
comp85649_c0_seq1	CTL4	0.1053733	0.3818889	0.5352222	0	3.624	0.039	0.000	< 0.0001
comp85242_c0_seq1	CTL6	1.510733	0.4088889	5.133333	0.339	0.271	0.001	0.066	< 0.0001
comp79487_c0_seq1	CTL13	0.1281222	0.0444667	1.312222	0	0.347	0.008	0.000	< 0.0001
comp82272_c0_seq1	CTL16	0.1892856	0	0.8393889	0	0.000	0.001	0.000	0.001
comp78334_c1_seq1	CTL17	0.3610889	0.1604444	1.862222	0	0.444	0.020	0.000	< 0.0001
comp78310_c0_seq2	CTL19	0	0.0043911	0	0.1027556	Infinity	0.040	Infinity	< 0.0001
comp80377_c0_seq1	CTL26	0.6052678	0.6994445	1.170667	0.2347778	1.156	0.542	0.201	< 0.0001
comp84005_c0_seq1	cSP4	0.1743	0.1673778	0.4063111	0.0238222	0.960	0.845	0.059	0.002
comp86273_c0_seq1	cSP6	0.57	0.397	1.666667	0.1132889	0.696	0.005	0.068	< 0.0001
comp84521_c0_seq1	cSP7	0.2537778	0.7544444	1.279667	0.0735	2.973	< 0.0001	0.057	< 0.0001
comp85451_c0_seq1	cSP29	3.284556	0.8548889	6.036666	0.2679	0.260	< 0.0001	0.044	0.001
comp86780_c0_seq1	cSP8	0.3713333	0.2486667	0.0604111	0	0.670	0.008	0.000	0.018
comp82961_c0_seq1	serpin-1	3.323333	3.196667	8.83	2.905555	0.962	0.742	0.329	0.002
comp86871_c1_seq1	serpin-3	3.385556	2.997778	6.971111	3.108889	0.885	0.153	0.446	0.010
comp90133_c0_seq4	serpin-4	2.595556	1.732222	6.451111	1.107333	0.667	0.014	0.172	< 0.0001
comp86728_c0_seq1	serpin-5	0.0494111	0.0172333	0	0.0365445	0.349	0.001	Infinity	0.003
comp86024_c0_seq1	serpin-6	3.555556	2.176667	9.974444	0.6842222	0.612	0.170	0.069	< 0.0001
comp86270_c0_seq1	serpin-7	0.2315444	0.1537778	0.4515556	0.1237667	0.664	0.169	0.274	0.010
comp86678_c0_seq1	serpin-9	0.3112111	0.9714444	0.8599667	0.3032222	3.121	< 0.0001	0.353	0.032
comp90752_c0_seq1	SP19	0.4232889	0.1994444	0.9683333	0.1690222	0.471	0.006	0.175	0.010
comp84735_c0_seq2	SP34	0.0879571	0.0286889	0	0	0.326	0.047		
comp87695_c1_seq1	SP48	0.3683111	0.3348889	1.094111	0.0423111	0.909	0.839	0.039	< 0.0001
comp89030_c0_seq2	SPH49	0.2593422	0.2834444	2.696889	0.1819333	1.093	0.761	0.067	0.001
comp88680_c0_seq2	SPH50	1.3251	1.857778	9.383333	2.292222	1.402	0.195	0.244	< 0.0001

comp88491_c0_seq2	PPO1	7	0.7371111	7.587778	3.004444	0.105	< 0.0001	0.396	0.002
comp89077_c0_seq1	PPO2	10.43556	0.7391111	9.503333	4.037778	0.071	< 0.0001	0.425	0.001
comp85443_c0_seq1	SOD1	0.2813333	0.3571111	0.2722445	0.7272222	1.269	0.241	2.671	0.001
comp84808_c0_seq1	SOD2	0.6898889	1.004778	3.813333	2.966667	1.456	0.008	0.778	0.142
comp82573_c0_seq1	Prx1	0.1241222	0.4148889	0.3212222	0.7888889	3.343	< 0.0001	2.456	< 0.0001
comp81640_c0_seq1	Prx5a	0.0357444	0.0175	0	0	0.490	0.009		
comp80284_c0_seq1	Lys1	2.868	3.954444	2.290111	0.5697778	1.379	0.518	0.249	0.024
comp82692_c2_seq1	Lys4	0.3344	0.0785778	0	0	0.235	0.001		
comp79271_c0_seq1	GST2	0.0708222	0.1140556	0.1065556	0.4504444	1.610	0.055	4.227	< 0.0001
