# Supplementary material for: Inhibition of melanization by serpin-5 and serpin-9 promotes baculovirus infection in cotton bollworm Helicoverpa armigera
Source: PLoS Pathog. 2017 Sep 27;13(9):e1006645. doi: 10.1371/journal.ppat.1006645 (PMC5633200; doi:10.1371/journal.ppat.1006645)
Supplement: S5 Table — (PDF) [file ppat.1006645.s012.pdf]

Supplementary Data 5. List of antibodies produced in this study

Assembly No.	Protein	Expression vector	Immunogen	Antibody	Dilution	M.W (kDa)
comp84005_c0_seq1	cSP4	pET-28a	Full length	Rabbit polyclonal	3000	36.8
comp86273_c0_seq1	cSP6	pET-28a	Full length	Rabbit polyclonal	3000	45
comp86780_c0_seq1	cSP8	pET-28a	Full length	Rabbit polyclonal	3000	39.5
comp85451_c0_seq1	cSP29	pET-28a	Full length	Rabbit polyclonal	3000	42.2
comp88491_c0_seq2	PPO1	pET-28a	Full length	Rabbit polyclonal	10000	78.6
comp89077_c0_seq1	PPO2	pET-28a	Full length	Rabbit polyclonal	10000	80
comp86728_c0_seq1	serpin-5	pET-28a	Full length	Rabbit polyclonal	5000	42.7
comp86678_c0_seq1	serpin-9	pET-32a	Full length	Rabbit polyclonal	5000	43
