# Supplementary material for: Inhibition of melanization by serpin-5 and serpin-9 promotes baculovirus infection in cotton bollworm Helicoverpa armigera
Source: PLoS Pathog. 2017 Sep 27;13(9):e1006645. doi: 10.1371/journal.ppat.1006645 (PMC5633200; doi:10.1371/journal.ppat.1006645)
Supplement: S6 Table — (PDF) [file ppat.1006645.s013.pdf]

Supplementary Data 6. Primers used for qPCR, protein expression, dsRNA synthesis and yeast two-hybrid assay.

**Primers for qPCR**		
**cSP4**	GGCAAACAGTTCAACCCAG	CCCATCCAGTTATTGTAAGCG
**cSP6**	GCAGAGTGGGCAACAAGAT	CCAGCGGTCAGGACGTATT
**cSP8**	TCCCTTGGATGGCGTTGT	CCACGTCTTGTTGCGTGTC
**cSP29**	CGTGCTGCCCAGATGATAT	CTTGCTGACGAGTGCTTAG
**serpin-5**	CTGTGTACCCAGCTTTCAGAG	CTCGATGTAGGTGTTAGCGAT
**serpin-9**	CGCCTCAGACACCTCGCT	CAGCGTGTCGTCCAAGAAGAT
**rpS3**	ACGGAGTTTTCAAGGCGGAA	GACTGCTCCGGGATGTTGAA
**Primers for protein expression in the *E. coli* expression system**		
**cSP4**	GCATGACTGGTGGACAGGAAGATGTTGGCGAGAGCTGTAT	CTAGTTATTGCTCAGCGG TTAAGCGTTGGGCCACACGAC
**cSP6**	GCATGACTGGTGGACAGCAAAATTCTTGCCAGACGC	CTAGTTATTGCTCAGCGGTTAAGGAACAATAGTGGTCCT
**cSP8**	GCATGACTGGTGGACAGCAAAGCAGCTGCGTCACACC	CTAGTTATTGCTCAGCGGTCAGACATTCGTGTTTTGTAGCG
**cSP29**	GCATGACTGGTGGACAGGCTAGTGTTAGTGAAGATGATT	CTAGTTATTGCTCAGCGGCTAGAAGCGCGCGTTCTG
**serpin-5**	GCATGACTGGTGGACAGCAAGTGGATTTCTATGAACG	CTAGTTATTGCTCAGCGGTCAATATACAGTAGGTTTAG
**serpin-9**	CGAATTCGAGCTCCGTCAGTGCGACTTGAAGACGG	GTGGTGGTGGTGGTGGTTAGAAGTCACTGGGCTTAGAAT
**PPO1**	CTAGTTATTGCTCAGCGGTCGGACGCCAAGAAGAACCTG	CTAGTTATTGCTCAGCGGCCGCCTCTGCTGCCTCGGTC
**PPO2**	CTAGTTATTGCTCAGCGGGCCGACGAAGAACAAAT	CTAGTTATTGCTCAGCGGTTAGTTGGTTCTTGGGTTCC
**Primers for protein expression in the *Drosophila* expression system**		
**cSP4**	CCGGAATTCGAAGATGTTGGCGAGAGCTGTATG	CTAGTCTAGAAGCGTTGGGCCACACG
**cSP6**	CCGGAATTCCAAAATTCTTGCCAGACG	CTAGTCTAGAAGGAACAATAGTGGTCCTAATCCAGTCCAG
**cSP4$_{Xa}$1**	CCGGAATTCTGAAGATGTTGGCGAGAGCTGTATG	GCTGCCTCACCTCCCAAGATCCGGCCTTCTATAGGAGGTATACTTGATTG
**cSP4$_{Xa2}$**	CAATCAAGTATACCTCCTATAGAAGGCCGGATCTTGGGAGGTGAGGCAGC	CTAGTCTAGAAGCGTTGGGCCACACGACGCTCTCGATCCAGTC
**cSP6$_{Xa}$1**	CCGGAATTCTCAAAATTCTTGCCAGACGCCCAGC	CGCTGTACCTCCCACGATCCGGCCTTCTATTCTGCTGTCCAAACCAC
**cSP6$_{Xa}$2**	CTGTGGTTTGGACAGCAGAATAGAAGGCCGGATCGTGGGAGGTACAGCG	CTAGTCTAGAAGGAACAATAGTGGTCCTAATCCAGTCCAGATATTCGTATACTTTG CTG
**Primers for yeast-two hybrid analyses**		
**cSP4**	CGTACCAGATTACGCTCATATG GAAGATGTTGGCGAGAGCTGTAT	GATTCATCTGCAGCTCGAGC TTAAGCGTTGGGCCACACGAC
**cSP6**	CGTACCAGATTACGCTCATATG CAAAATTCTTGCCAGACGC	GATTCATCTGCAGCTCGAGC TTAAGGAACAATAGTGGTCCT
**cSP8**	CGTACCAGATTACGCTCATATG GCTAGTGTTAGTGAAGATGATT	GATTCATCTGCAGCTCGAGC CTAGAAGCGCGCGTTCTG
**serpin-5**	ATCTCAGAGGAGGACCTGCATATG CAAGTGGATTTCTATGAACG	CGCTGCAGGTCGACGGAT TCAATATACAGTAGGTTTAG
**serpin-9**	ATCTCAGAGGAGGACCTGCATATG CAGTGCGACTTGAAGACGG	CGCTGCAGGTCGACGGAT TTAGAAGTCACTGGGCTTAGAAT

Primers for dsRNA synthesis		
Primers used for amplification contains T7 sequence at 5'	(TAATACGACTCACTATAGGG)	
**T7-cSP4**	GATGTTGGCGAGAGCTGTAT	GGTGAACTGTAGAGGTCTCT
**T7-cSP6**	AGCGACAACAGTAGACCAAT	GGCATCTGTCCTGGCTAAC
**T7-cSP8**	CCAATGTGGAGGTGTTCTGAT	CCTTCAGGTCTTCGCTTCAT
**T7-cSP29**	GCCGAGACTAGAGGTTGTG	CCTGACAAGCGTCCTTTC
**T7-serpin-5**	AGCCCGCATCGCTAACAC	TAGAAACGGCAGACGCCAC
**T7-serpin-9**	TCAGACACCTCGCTCCAGG	CCACTGTCCTTGGTCGATG
**T7-GFP**	CACAAGTTCAGCGTGTCCG	GTTCACCTTGATGCCGTTC
